# *Vibrio parahaemolyticus* and *Vibrio vulnificus in vitro* biofilm dispersal from microplastics influenced by simulated human environment

**DOI:** 10.3389/fmicb.2023.1236471

**Published:** 2023-10-03

**Authors:** Ryan E. Leighton, Liyan Xiong, Gracie K. Anderson, Grace M. Astarita, Guoshuai Cai, Robert Sean Norman, Alan W. Decho

**Affiliations:** ^1^Department of Environmental Health Sciences, University of South Carolina, Columbia, SC, United States; ^2^Department of Environmental Health Sciences, NIEHS Center for Oceans and Human Health and Climate Change Interactions, University of South Carolina, Columbia, SC, United States

**Keywords:** *Vibrio vulnificus*, *Vibrio parahaemolyticus*, biofilms, microplastics, biofilm dispersal, human plasma-like medium, simulated gastric fluids

## Abstract

Growing concerns exist regarding human ingestion of contaminated seafood that contains *Vibrio* biofilms on microplastics (MPs). One of the mechanisms enhancing biofilm related infections in humans is due to biofilm dispersion, a process that triggers release of bacteria from biofilms into the surrounding environment, such as the gastrointestinal tract of human hosts. Dispersal of cells from biofilms can occur in response to environmental conditions such as sudden changes in temperature, pH and nutrient conditions, as the bacteria leave the biofilm to find a more stable environment to colonize. This study evaluated how brief exposures to nutrient starvation, elevated temperature, different pH levels and simulated human media affect *Vibrio parahaemolyticus* and *Vibrio vulnificus* biofilm dispersal and processes on and from low-density polyethylene (LDPE), polypropylene (PP), and polystyrene (PS) MPs. Both species were able to adequately disperse from all types of plastics under most exposure conditions. *V. parahaemolyticus* was able to tolerate and survive the low pH that resembles the gastric environment compared to *V. vulnificus.* pH had a significantly (*p* ≤ 0.05) positive effect on overall *V. parahaemolyticus* biofilm biomass in microplates and cell colonization from PP and PS. pH also had a positive effect on *V. vulnificus* cell colonization from LDPE and PP. However, most biofilm biomass, biofilm cell and dispersal cell densities of both species greatly varied after exposure to elevated temperature, pH, and nutrient starvation. It was also found that certain exposures to simulated human media affected both *V. parahaemolyticus* and *V. vulnificus* biofilm biomass and biofilm cell densities on LDPE, PP and PS compared to exposure to traditional media of similar pH. Cyclic-di-GMP was higher in biofilm cells compared to dispersal cells, but exposure to more stressful conditions significantly increased signal concentrations in both biofilm and dispersal states. Taken together, this study suggests that human pathogenic strains of *V. parahaemolyticus* and *V. vulnificus* can rapidly disperse with high cell densities from different plastic types *in vitro*. However, the biofilm dispersal process is highly variable, species specific and dependent on plastic type, especially under different human body related environmental exposures.

## Introduction

*Vibrio parahaemolyticus* and *Vibrio vulnificus* are two known pathogenic species that naturally exist in the marine environment and can infect both marine animals and humans. These gram-negative pathogens commonly infect humans through consumption of contaminated raw seafood (Elmahdi et al., 2018). While human infections are rare, symptoms of both *V. parahaemolyticus* and *V. vulnificus* infections can include cramps, nausea, fever, and bloody diarrhea. *V. vulnificus* foodborne infections can be more severe, and is considered one of the most fatal foodborne pathogens in the United States, with a fatality rate of around 50% in susceptible human hosts (Jones and Oliver, 2009; Oliver, 2015; Centers for Disease Control and Prevention, 2019). *V. vulnificus* can also infect humans and cause deadly skin infections through marine water contact with open wounds (Oliver et al., 2012). *Vibrio*’s habitat range has increased likely due to climate change providing more optimal growth conditions, which has coincided with an increase in plastic production (Froelich and Daines, 2020; Maquart et al., 2022). At the same time, an increase in temperature and carbon and nitrogen pollution has led to increased prevalence of cyanobacterial harmful algal blooms (HABs) (O’Neil et al., 2012; Visser et al., 2016). As HAB outbreaks have also been suggested to enhance *V. parahaemolyticus* and *V. vulnificus* growth, understanding how an increase in temperature and the presence of nutrients may affect *Vibrio* biofilm growth is also of importance (Greenfield et al., 2017). This expansion of *Vibrio* coupled with their inherent propensity to colonize, persist, and disperse from numerous plastic types will increase the future potential infection risks to humans, especially by ingestion of contaminated seafood (Keswani et al., 2016; Bowley et al., 2021; Cverenkárová et al., 2021).

Both *V. parahaemolyticus* and *V. vulnificus* often exist in biofilms, which are bacterial communities enclosed in a protective, self-secreted matrix of extracellular polymeric substances (EPS) (Decho and Gutierrez, 2017). Both species have been found attached to biotic or abiotic surfaces, which can include carapaces, algae, and specifically microplastics (MPs) (De Tender et al., 2015; Dang and Lovell, 2016; Kirstein et al., 2016; Lage and Graça, 2016). A biofilm’s natural lifecycle consists of cell attachment to a surface, followed by growth and biofilm development, and lastly periodic detachment of cells, called passive dispersion (Flemming et al., 2007; Kaplan, 2010). Biofilms have been implicated in over 75% of documented microbial infections (Davies, 2003; Guilhen et al., 2017). Biofilm related infections can be influenced by the surrounding environment, and one of the infection mechanisms is called active environmentally induced biofilm dispersion, which triggers the release of bacteria from biofilms into the hosts’ internal environment (Wang et al., 2011; Marks et al., 2013; Jamal et al., 2018; Rumbaugh and Sauer, 2020; Roy et al., 2021).

Dispersion facilitates the transfer of bacteria to new sites for colonization to repeat their biofilm lifecycle (Purevdorj-Gage et al., 2005). Active, environmentally induced dispersal of cells from biofilms can occur due to sensing of intracellular dispersion cues, which can be induced by sudden changes in temperature, pH, and nutrient conditions. This is accomplished by a membrane-associated protein complex that relays signals via a series of post-transcriptional modifications that can change the concentration of the intracellular signaling molecule cyclic-di-GMP (c-di-GMP) (Rumbaugh and Sauer, 2020). C-di-GMP can control and trigger the motile and sessile forms of the biofilm cell lifecycle (Galperin, 2004; Flemming et al., 2007; Römling et al., 2013; Lami, 2019). Different concentrations of c-di-GMP in the cell lead to different actions, with high concentrations leading to biofilm formation and low concentrations leading to dispersion (Valentini and Filloux, 2016).

Dispersed cells have a phenotype between that of biofilm and planktonic cells, and have been found to display higher motility, virulence, adherence, and altered antibiotic resistance compared to their own biofilm and planktonic cells (Chua et al., 2014; Rumbaugh and Sauer, 2020). Biofilm dispersal can be a major mechanism of ingested foodborne and waterborne bacterial infections in humans (Abdallah et al., 2014). This likely occurs as cells embedded in biofilms survive the sudden changes in temperature and pH in a new environment like the human gastrointestinal tract. When environmental conditions are more favorable, the cells disperse from the consumed abiotic or biotic surface, which can lead to colonization of the gastrointestinal surfaces (Motta et al., 2021; Xiong et al., 2022). One of these abiotic surfaces can be MPs, and since *Vibrio* biofilms have been abundantly found on MPs, one implication is that MPs could act as transport vectors of pathogenic *Vibrio* species to marine animals that coincidently or selectively ingest biofilm-associated MP particles instead of food particles in the marine environment. Consequently, consumption of raw seafood contaminated with *Vibrio* that dispersed from, or living on MPs could lead to higher human exposure to potential pathogenic *Vibrio* species and thus higher infection rates (Goldstein et al., 2014; Reisser et al., 2014; Kirstein et al., 2016; Viršek et al., 2017).

There are several major types of plastic, which include polyethylene (PE), polypropylene (PP) and polystyrene (PS). Due to their high production and usage, there is a high probability that these plastic types can end up in marine environments, usually from direct introduction or runoff (Andrady, 2003; Brien, 2007; Andrady, 2011; Eriksen et al., 2014; Lusher et al., 2017). Weathering or degradation of macroplastics in the environment can lead to MPs which are classified as plastic particles smaller than 5 millimeters in size (Arthur et al., 2009; Barnes et al., 2009; Eriksen et al., 2014; Lambert et al., 2014; Kershaw and Rochman, 2015). MPs have been found in several seafood species in the marine environment including clams and shrimp, with fiber, film and sphere MPs being some of the most abundant types, and concentrations as high as an average of 7,000 particles per shrimp and low as 5 particles per gram of clams (Davidson and Dudas, 2016; Waite et al., 2018; Curren et al., 2020). Recent studies conducted in marine environments have found bacterial communities, including *V. parahaemolyticus* and *V. vulnificus*, associate and live on MP surfaces (Kesy et al., 2021; Xu et al., 2021; Pedrotti et al., 2022). This could have serious implications for both marine life and human health, as MPs could be contributing to higher pathogenic *Vibrio* concentrations in contaminated raw seafood and thus increased exposure to humans by ingestion, or increased exposure by physical contact with biofilm-associated MPs in marine environments (Ward and Kach, 2009; Zettler et al., 2013; Keswani et al., 2016). *Vibrio* biofilms on MPs and their sudden response to conditions likely encountered in the human host by ingestion such as elevated temperature, nutrient starvation and decreased pH is not well understood (Reidl and Klose, 2002; Oberbeckmann et al., 2015; Hernández-Cabanyero et al., 2019). However, to better assess potential changes in bacterial responses to the human environment, the use of simulated human-like media also needs to be implemented in non-invasive studies and models. This mimics components of the digestive system like simulated gastric fluid (SGF) and simulated intestinal fluid (SIF), and simulated components of the circulatory system like Human Plasma-Like Medium (HPLM).

Synthetic human-like media have been recently developed to better model the human environment. Exposing different bacteria to these medias can improve our understanding of how pathogens respond to these simulated human conditions as a proxy for *in vivo* studies. For example, use of simulated gastric fluids (SGF) and simulated intestinal fluids (SIF) can improve our understanding of how pathogenic *Vibrio* may survive aspects of human digestion and then colonize the human host (Wang et al., 2019). While HPLM has been reported in a few studies that examined specific human cell type culture and shows promise in becoming a standard media for human cell culture, using this HPLM for assessing human bacterial pathogens has not yet been reported (Cantor, 2019; Leney-Greene, et al., 2020; Rossiter et al., 2021). Using HPLM to assess pathogenic *Vibrio* species *in vitro* biofilm response to conditions that mimic human plasma and body temperature could lead to better optimization of noninvasive studies and models, especially in clinical settings. Human environmental factors can influence biofilm growth and viability, but there is an underlying knowledge gap regarding how sudden changes that mimic the human environment affect *V. parahaemolyticus* and *V. vulnificus* biofilm biomass and dispersal processes, especially on and from low-density polyethylene, polypropylene, and polystyrene MPs.

In this study, we examined the effect of temperature, pH, nutrient availability, and media composition on *in vitro* biofilm dispersal processes by human isolated *V. parahaemolyticus* and *V. vulnificus*. We evaluated overall biofilm dispersal and viability in microplates, and then dispersal from low-density polyethylene (LDPE), polypropylene (PP), and polystyrene (PS) MPs. We also examined how c-di-GMP production and concentration were impacted by media composition and cell state (biofilm vs. dispersed cells). We hypothesized that both *Vibrio* species would have overall decreased biofilm formation and cell dispersion in microplates when suddenly exposed to lower pHs and higher temperatures, especially in nutrient starved conditions due to stress. Both *Vibrio* species should have greater overall biofilm formation and cell dispersion in microplates when suddenly exposed to higher pH and temperature as conditions become more favorable. Both *Vibrio* species should also disperse from all MP types tested when exposed to these same favorable conditions. Both human isolated strains should have increased cell dispersal in simulated human media as their infection response to simulated human conditions. Exposure to SGF should lead to decreased cell dispersion due to low pH while exposure to SIF should lead to increased cell dispersion due to higher pH. C-di-GMP concentrations should be higher in the biofilm state compared to dispersal state, with higher concentrations in dispersal cells in more stressful conditions.

## Materials and methods

### Bacterial strains and growth conditions

Two clinical strains were obtained from the American Type Culture Collection (Table 1, ATCC, Manassas, VA, United States). *V. parahaemolyticus* strains are commonly classified by their species marker (*tlh*) and capacity to infect humans through production of thermostable direct hemolysin (*tdh*) or thermostable direct hemolysin-related hemolysin (*trh*) virulence factors (Honda and Iida, 1993; Broberg et al., 2011). In our study, human isolated strain ATCC17802 contained *tlh* and *trh* and its human estimated threshold mean infective dose (ID_50_) is listed in Table 1. While *V. vulnificus* strains can also be classified by virulence factors, *V. vulnificus* can also be classified by 16S rRNA typing, which reveals if they are more clinically – (type B, higher possible human infectivity) or more environmentally – (type A, higher possible marine vertebrate infectivity) associated. In our study, the human isolate strain ATCC27562 is type B and its human estimated threshold mean infective dose (ID_50_) is also listed in Table 1.

**Table 1:** *Vibrio* strains used in this study.

Species	Isolation source	Strain ID	Isolate origin	Characteristics	Estimated human^1^ threshold ID_50_
*V. parahaemolyticus*	ATCC	ATCC17802	Human (enteric)	*tlh/trh*	10^5^ – 10^8^ CFUs^2,3,4^
*V. vulnificus*	ATCC	ATCC27562	Human (blood)	16S Type B	10^3^ CFUs^4,5^

^1^Susceptible, usually with pre-existing, underlying health conditions. ^2^Marx et al. (2013). ^3^Food and Drug Administration (2010). ^4^Food and Drug Administration (2012). ^5^World Health Organization (2005).

The two clinical strains were tested for biofilm formation and dispersal after exposure to different temperatures, pH, nutrients, and media compositions. All strains were maintained in 25% (vol/vol) glycerol at −80°C to be used in further experiments. A single colony of each bacteria was inoculated in 5 mL unadjusted modified seawater with yeast extract (MSYE, ATCC medium 804, Oliver and Colwell, 1973) broth supplemented with calcium chloride (1.8 g/L), as calcium chloride contributes to biofilm formation (Tischler et al., 2018), and incubated overnight at 30°C with shaking at 180 revolutions/min (rpm). After incubation, the broth culture was adjusted to 10^7^ colony forming units (CFU)/mL (OD_600_) using a SpectraMax M3 plate reader after calibrating the instrument’s absorbance values to cell counts from spread plating (Molecular Devices, San Jose, CA, United States), and adjusted culture was used for subsequent experiments. The recipe of MSYE was adjusted to remove nutrients (peptone and yeast extract), and for the purpose of this study was called modified seawater (MS) for nutrient starvation experiments. The pH of MSYE and MS was adjusted using 1 M hydrochloric acid and 1 M sodium hydroxide using a SevenExcellence^™^ pH probe (Mettler-Toledo, Columbus, OH, United States) after pH standard calibration. HPLM was obtained from Thermo Fisher (Waltham, United States) while SGF (supplemented with pepsin) and SIF (supplemented with trypsin and pancreatin) were obtained from Biochemazone^™^ (Ontario, Canada).

### Biofilm formation in microplates

Biofilm formation experiments in microplates were adapted from O'Toole (2011). In summary, adjusted cell densities (10^7^ CFU/mL) were diluted 1:100 in fresh MSYE supplemented with calcium chloride media to reach a final density of 10^5^ CFU/mL. Then 150 μL of the diluted culture was pipetted into clear tissue-culture treated 96-well polystyrene microplates (Costar®, Corning, NY, United States) for OD_600_ and OD_570_ readings or black walled, clear bottom tissue-culture treated 96-well polystyrene microplates for fluorescence readings. Then, the 96-well plates were incubated at 25°C with low shaking (125 rpm) for 24 h to form biofilms. Wells containing MSYE supplemented with calcium chloride without inoculation were used as blank and group controls. Low shaking conditions, instead of static, were chosen to introduce shear stress to the biofilms, to better resemble the marine and human environments in all microplate biofilm formation and dispersal experiments. All plates in biofilm formation and dispersal experiments were sealed with Parafilm^™^ (Bemis, Neenah, WI, United States) to prevent evaporation of media.

### Biofilm dispersal microplate screening assay

Biofilms of both *V. parahaemolyticus* and *V. vulnificus* were dispersed according to Gjermansen et al. (2005) and Barraud et al. (2014) with some modifications. Briefly, after 24-h spent media and planktonic cells were removed from the 96-well microplates and the biofilms gently washed three times with 175 μL 1X phosphate buffer saline (PBS, Molecular Biologicals International, Irvine, CA, United States). Wells were then refilled (150 μL) with the specific media types and incubated under the environmental conditions outlined in Table 2. Wells containing media without inoculation were used as blank and group controls. The optical density of each well was measured at λ = 600 nm using a SpectraMax M3 plate reader (Molecular Devices, San Jose, CA, United States) every 15 min for two hours at 25 or 37°C to initially screen for the media and environmental conditions that led to cell dispersal for later microplastic dispersal experiments.

**Table 2:** Environmental conditions used in this study.

Media type	pH	Temperature (°C)	Exposure time (h)	Shaking speed (rpm)
Modified seawater yeast extract (MSYE)	3, 4, 5, 6, 7, 7.4, 8.1	25, 37	2	125
Modified seawater (MS)	3, 4, 5, 6, 7, 7.4, 8.1	25, 37	2	125
Simulated gastric fluid w/pepsin (SGF)	1.5	25, 37	2	125
Simulated intestinal fluid w/trypsin and pancreatin (SIF)	6.8	25, 37	2	125
Human plasma-like media (HPLM)	7.4	25, 37	2	125

### Crystal violet staining microplate screening assay

Biofilms of both *V. parahaemolyticus* and *V. vulnificus* in microplates were quantified by crystal violet staining according to O'Toole (2011) and Valquier-Flynn et al. (2017) with some modifications. Following two-hour exposures, planktonic cells were removed and then each well containing biofilms was gently washed three times with 175 μL 1X PBS. 175 μL of 100% methanol (Sigma-Aldrich, St. Louis, MO, United States) was then added to the plates to fix the biofilms to the plates at room temperature for 20 min. Then, the methanol was removed, and residual methanol allowed to evaporate from plates in fume hood. Biofilms were stained with 150 μL of 0.1% (wt/vol) crystal violet (Sigma-Aldrich, St. Louis, MO, United States) for 15 min at room temperature. Staining solution was removed via pipette, and then 175 μL 1X PBS was used three times to remove the non-bound dye. The stained and washed biofilms were air dried overnight in a fume hood, then 150 μL of 30% acetic acid (Fisher Scientific, Hampton, NH, United States) was added to dissolve the bound crystal violet for 15 min. 125 μL of the solubilized crystal violet acetic acid solution was then transferred to a new 96-well clear polystyrene microplate, and optical densities of each well were measured by absorbance (570 nm) using a SpectraMax M3 plate reader to initially screen for conditions that led to changes in biofilm biomass for later microplastic dispersal experiments.

### Resazurin biofilm viability screening assay

The viability of both *V. parahaemolyticus* and *V. vulnificus* biofilms were examined after being exposed to different conditions (Table 2) by a resazurin metabolic assay according to Riss et al. (2004) with some modifications. After 24 h, spent media and planktonic cells were removed from the black walled, clear bottom 96-well microplates before gently washing three times with 175 μL 1X PBS. Then, wells were refilled (150 μL) with media and incubated in conditions in Table 2 at 37°C. After incubation, the planktonic cells were removed and then each well was gently washed with 175 μL 1X PBS three times. Then, each well was refilled with 150 μL of unadjusted MSYE supplemented with calcium chloride and 30 μL of filter sterilized resazurin (0.15 mg/mL in 1X PBS) was also added to each well. Fluorescence was read (excit/emiss = 560/590 nm) every 15 min for four hours at 37°C to screen for conditions that potentially led to changes in biofilm viability. Final RFU values were then normalized against their corresponding OD_600_ values after exposure. Wells containing media without inoculation were used as blank and group controls.

### Biofilm formation on MPs

Biofilm formation experiments on MPs were adapted from O'Toole (2011), Hamanaka et al. (2012), Valquier-Flynn et al. (2017), and Leighton et al. (2023). In summary, MPs were generated from slide coupons of low-density polyethylene (LDPE), polypropylene (PP), and polystyrene (PS) (Table 3, Biosurface Technologies, Boseman, MT, United States) by cutting the coupons to dimensions of 4 mm x 1 mm. Plastics were chemically sterilized (70% ethanol for PP, 70% isopropanol for LDPE and PS) for 24 h and were then placed in sterile petri dishes in a biosafety cabinet until residual alcohol evaporated. Chemically sterilized MPs were then placed in 96-well sterile non-treated microplates (Costar^®^, Corning, NY, United States) and then adjusted cell densities (10^7^ CFU/mL) were diluted 1:100 in fresh MSYE with calcium chloride media to reach a final density of 10^5^ CFU/mL. Microplates containing MPs were filled with 150 μL of inoculum, then the microplates were incubated at 25°C with low shaking (125 rpm) to form biofilms in 24 h. Wells containing media without inoculation and with MPs were used as blank and group controls.

### Biofilm dispersal MP assay

Biofilm dispersal experiments were adapted from Hamanaka et al. (2012) and Valquier-Flynn et al. (2017) with slight modifications. Following 24 h incubation, spent media and planktonic cells were removed from the microplate wells and MPs containing biofilms were gently washed three times with 175 μL 1X PBS, and then MPs were transferred to new wells containing 150 μL of the specific media types and incubated under environmental conditions listed in Table 2. Wells containing media without inoculation were used as blank and group controls.

**Table 3:** Coupon types and characteristics used in this study.

Coupon type	Chemical formula	Density	Length/thickness	Surface area	Usage
Low-density polyethylene	(C₂H₄)ₙ	0.96 g/cm^3^	4 mm/1.6 mm	24 mm^2^	Plastic bags, six-pack rings, packaging film, bottles, and netting
Polypropylene	(C_3_H_6_)_n_	0.93 g/cm^3^	4 mm/1.6 mm	24 mm^2^	Bottle caps, packaging film, and netting
Polystyrene	(C_8_H_8_)_n_	1.6 g/cm^3^	4 mm/0.6 mm	14 mm^2^	Plastic utensils and food containers

### Crystal violet staining MP assay

Biofilms of both *V. parahaemolyticus* and *V. vulnificus* on MPs were quantified by crystal violet staining according to Leighton et al. (2023) with some modifications. After the remainder of the planktonic cells were removed, the MPs were gently washed three times in 175 μL 1X PBS, and then 175 μL of 100% methanol was added per well to fix the biofilms and incubated at room temperature for 20 min. Then, the methanol was removed, and residual methanol allowed to evaporate off MP surfaces in fume hood. The biofilms were stained with 150 μL of 0.1% (wt/vol) crystal violet for 15 min at room temperature. The staining solution was removed, and then 1X PBS was used to remove the non-bound dye three times. The MPs were then transferred to a new 96-well non-treated microplate and the stained and washed biofilms on both MPs and their respective microplates were air dried overnight in fume hood. Lastly, 150 μL of 30% acetic acid was added to dissolve the bound crystal violet and incubated at room temperature for 15 min. The optical density of each well was measured at a wavelength of 570 nm using a SpectraMax M3 plate reader. Mean OD_570_ values were then divided by the surface area (24mm^2^ – PP & LDPE, 14mm^2^ – PS) of the plastics tested to obtain final biofilm biomass values/mm^2^ of the surface type.

### Determination of dispersed biofilm cell densities from MPs

Total dispersed biofilm cell densities were determined after biofilms on MPs were exposed to different pHs, temperatures, nutrient availability, and media compositions. After incubation, 100 μL of each well was either taken and serially diluted (10^−2^ to 10^−7^ CFU/mL) in 900 μL 1X PBS in microcentrifuge tubes, or directly spread plated (10^−1^ CFU/mL) onto prewarmed MSYE supplemented with calcium chloride agar plates for determination of number of dispersed cells. Plates were incubated at 37°C for 20–24 h. Biofilm dispersed cells were determined in terms of CFU/mm^2^ and then log transformed to obtain final values.

### Biofilm removal and determination of colony counts

Total colony counts were determined from biofilm suspensions according to Bjerkan et al. (2009), Portillo et al. (2013), and Leighton et al. (2023) with some modifications. Following 24 h incubation, planktonic cells were removed from the 96-well non-treated microplate wells before gently washing MPs three times with 175 μL of 1X PBS. Then, plastics were placed individually in 1 mL of 1X PBS in sterile borosilicate culture tubes (VWR International, Radnor, PA, United States) with a rubber cap and vortexed using a Vortex Genie 2^®^ (Fisher Sci.) at the highest setting for one minute. The borosilicate glass tubes containing the plastics and 1X PBS solution were then placed in a Branson M2800 ultrasonication water bath (Branson Ultrasonics, Brookfield, CT, United States) and sonicated for five minutes at 40 kHZ. The glass tubes were vortexed again for one minute. Lastly, the biofilm suspension in 1X PBS was serially diluted in 1X PBS in microcentrifuge tubes and 10^−1^ to 10^−7^ serial dilutions were spread onto prewarmed MSYE supplemented with calcium chloride agar plates. Plates were incubated at 37°C for 20–24 h. The viability of cells was determined as CFU/mm^2^. The biofilm cell densities of each plastic group had biological triplicates and each experiment was conducted three times independently. Mean CFU values were then divided by the surface area (24 mm^2^ – PP & LDPE, 14 mm^2^ – PS) of the plastics tested to obtain CFU/mm^2^ of the surface type. CFU values were then log-transformed to obtain final values.

### Cyclic-di-GMP assay

Estimation of c-di-GMP levels of both *V. parahaemolyticus* and *V. vulnificus* were examined after being exposed to MSYE, SIF and HPLM at 37°C for two hours by a cyclic-di-GMP assay kit (Lucerna, Brooklyn, NY, United States) according to manufacturer’s instructions with some modifications. Briefly, 100 μL of dispersed cells were diluted 1:10 in RNase-free water after environmental exposure. Sterilized swabs were used to collect biofilms, then swabs were submerged and vortexed in 150 μL of RNase-free water, and 100 μL of biofilm cells were diluted 1:10 in RNase-free water. Then, 50 μL of diluted culture along with assay reagents and serially diluted c-di-GMP standards were set up for the assay, and then the c-di-GMP concentration was calculated according to the standard calibration curve. Appropriate sample dilution factors were multiplied to get the final c-di-GMP concentrations (in picograms per microliter).

### Statistical analyses

The experimental data for biofilm biomass, biofilm CFUs, cell colonization, dispersal CFUs and c-di-GMP concentrations were expressed as the mean ± standard deviation. Experimental data for biofilm viability were expressed as the mean and then normalized against corresponding mean OD_600_ values. Two-way analysis of variance (ANOVA) models were calculated using Rstudio software to compare value differences (α = 0.05) in biofilm biomass, biofilm CFUs, cell colonization and dispersal CFUs. For the first set of models, temperature, pH and nutrient content were the variables in examining value differences between nutrient rich and nutrient starved conditions at different temperature and pH intraspecies. 25°C was selected as the reference temperature, MSYE as the reference media and pH as a continuous variable for all analyses. For the second set of models, temperature and media composition were the variables in examining value differences between simulated human medias and MSYE intraspecies, and *Vibrio* species was a variable for examining value differences interspecies. 25°C was selected as the reference temperature, MSYE with similar pH to simulated human medias as the reference medias, and *V. parahaemolyticus* as the reference species for all analyses. One-way ANOVAs (α = 0.05) and t-tests were calculated for comparison between c-di-GMP levels within and between biofilm and dispersal cells after exposure to different medias using Excel’s data analysis toolpak. Bonferroni corrections were calculated and applied to all *p*-values to control for type 1 errors.

## Results

Experiments and statistical analyses were conducted to test the effect and differences in temperature (25, 37°C), pH (3, 4, 5, 6, 7, 7.4, 8.1), nutrient availability (MS) and simulated human media composition (HPLM, SGF, SIF) after two-hour exposure on overall biofilm biomass, cell viability, dispersal and c-di-GMP concentrations in microplates and biofilm biomass, cell viability, dispersal and colonization on/from LDPE, PP and PS MPs by human isolated strains of *V. parahaemolyticus* and *V. vulnificus*. All raw means and statistical data are presented in Supplementary Tables S1–S61.

The crystal violet staining assays reflected total bacterial biomass (expressed as OD_570_ values) on microplates, MP surface types, and subsequent microplate colonization from dispersal of these MP surfaces. Cell concentration assays reflected cell dispersal in microplates (expressed as OD_600_ values), and biofilm cell and biofilm dispersal cell densities (expressed as colony forming units, CFUs) on/from the substate surface types. Resazurin metabolic assays reflected biofilm cell viability (expressed as RFUs). C-di-GMP assays reflected biofilm and cell dispersal state signaling molecule concentrations (expressed as pg./μL).

### Temperature, pH and nutrients affect *Vibrio parahaemolyticus* in microplates

**Figure 1:**
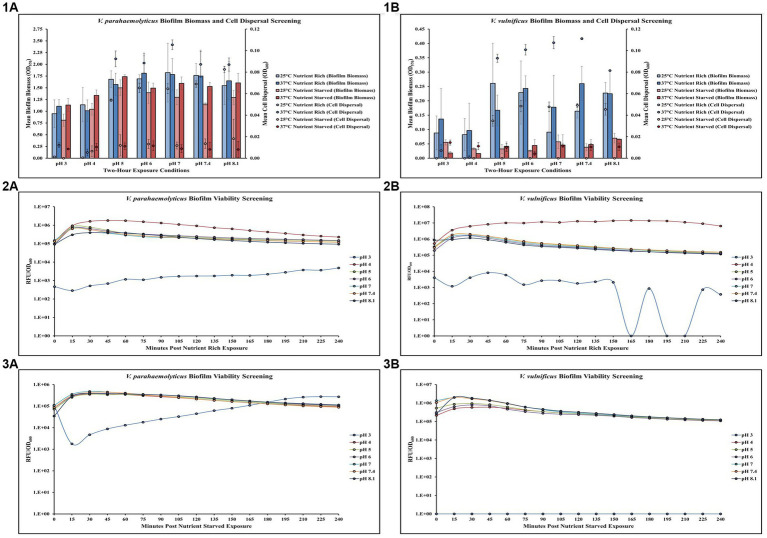
Exposure to elevated temperature, changes in pH, and nutrient starvation influence *V. parahaemolyticus* and *V. vulnificus* biofilm biomass, cell dispersal and viability in microplates. Comparison of overall biofilm biomass and cell dispersal (values represent *X* ± SD, *n* = 3 replicate samples from three independent experiments) of *V. parahaemolyticus* **(1A)** and *V. vulnificus* **(1B)** between different pH, temperature, and nutrient availability (means of all biological triplicates and three independent experiments after 2-hour exposure). Comparison of biofilm viability (RFU/OD600) of *V. parahaemolyticus* after 2-hour exposure to nutrient rich **(2A)** and nutrient starved **(3A)** conditions and *V. vulnificus* after 2-hour exposure to nutrient rich **(2B)** and nutrient starved **(3B)** conditions at different pH at 37°C.

**Figure 2:**
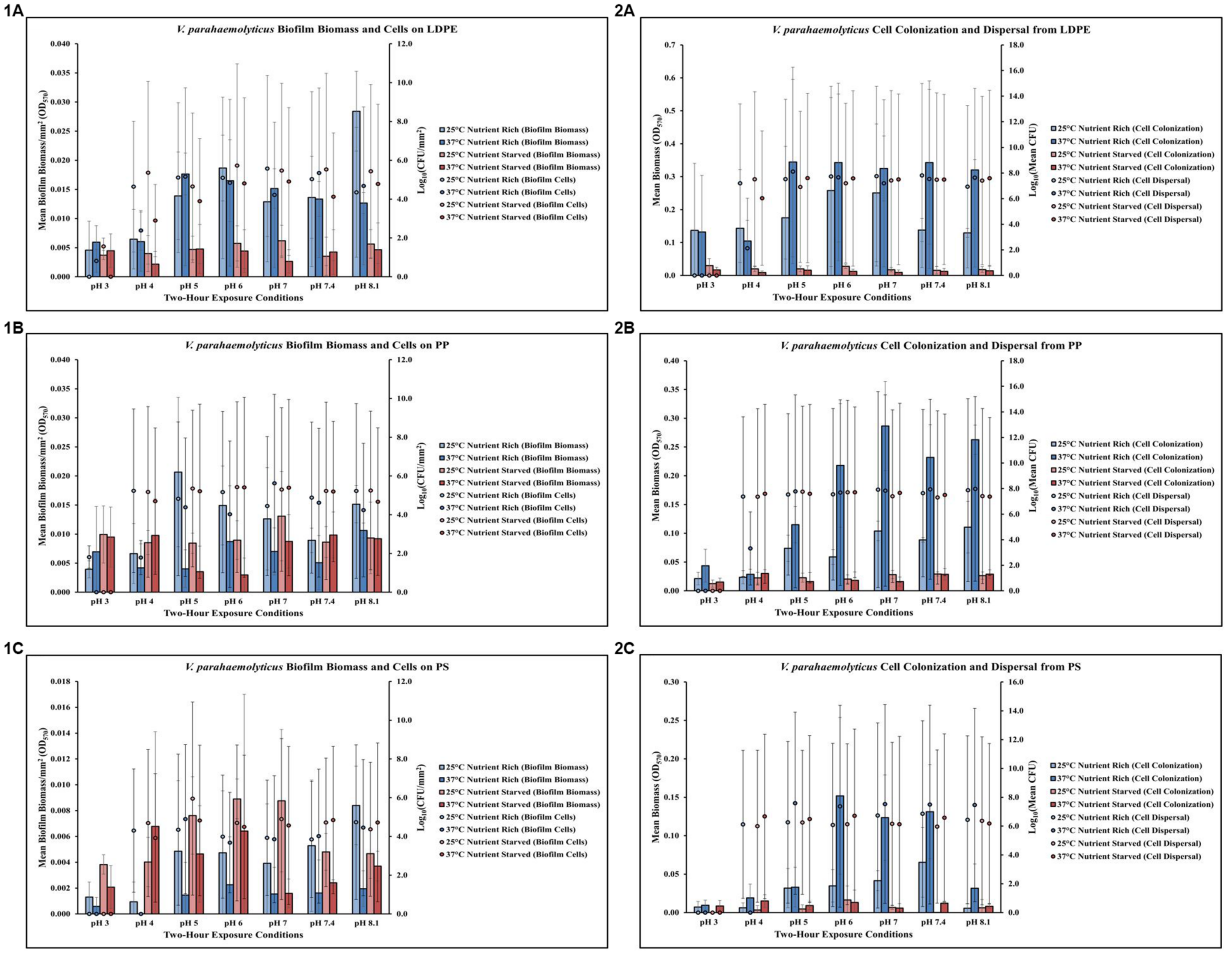
Exposure to elevated temperature, changes in pH, and nutrient starvation influence *V. parahaemolyticus* biofilm processes on and from LDPE, PP, and PS MPs. Comparison of biofilm biomass and CFUs (values represent *X* ± SD, *n* = 3 replicate samples from three independent experiments) on LDPE **(1A)**, PP **(1B)**, and PS **(1C)** and subsequent biofilm cell dispersal CFUs and microplate colonization (values represent *X* ± SD, *n* = 3 replicate samples from three independent experiments) from LDPE **(2A)**, PP **(2B)**, and PS **(2C)** after 2 h exposure to different pH, temperature and nutrient availability.

**Figure 3:**
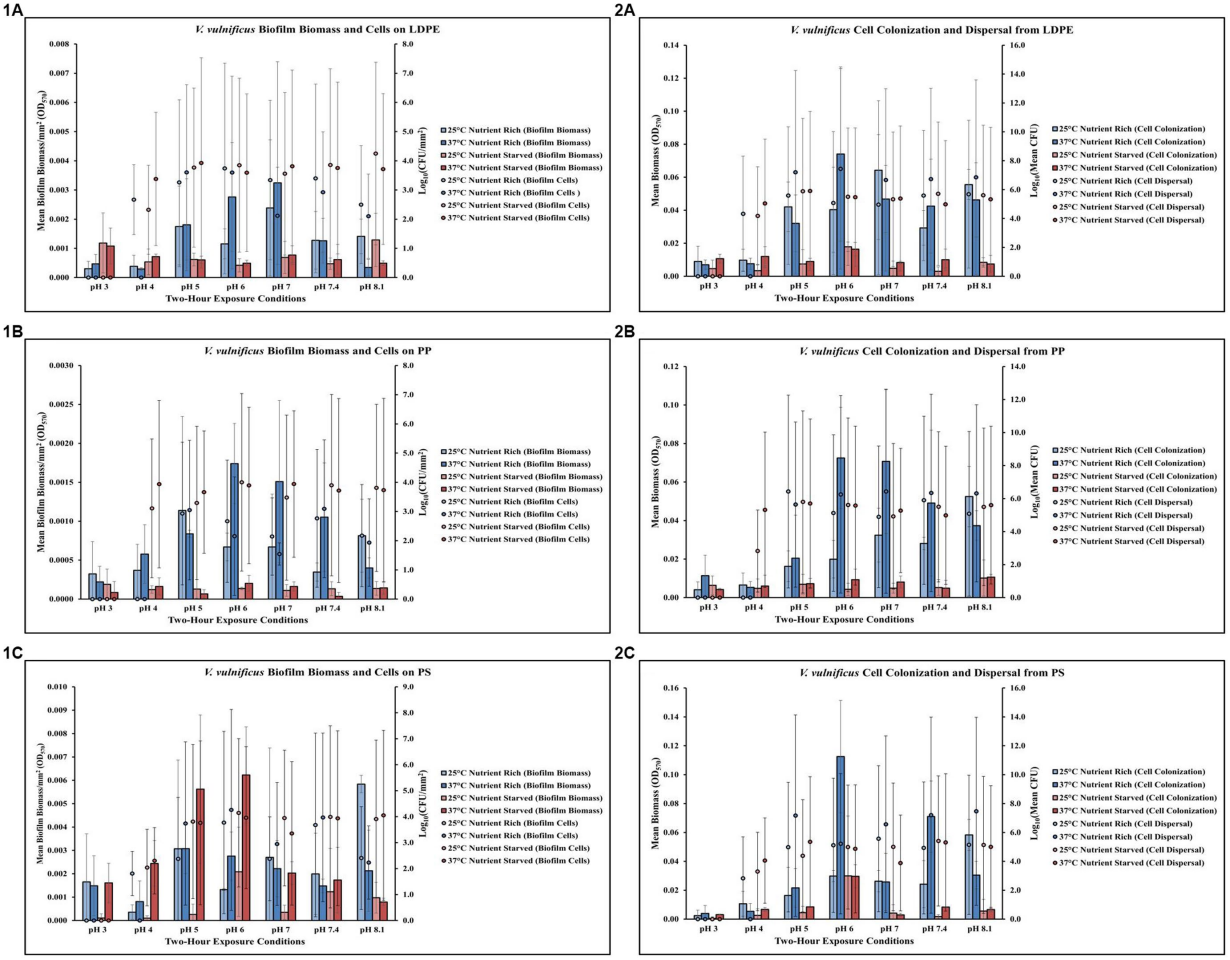
Exposure to elevated temperature, changes in pH and nutrient starvation influence *V. vulnificus* biofilm processes on and from LDPE, PP, and PS MPs. Comparison of biofilm biomass and CFUs (values represent *X* ± SD, *n* = 3 replicate samples from three independent experiments) on LDPE **(1A)**, PP **(1B)**, and PS **(1C)** and subsequent biofilm cell dispersal CFUs and microplate colonization (values represent *X* ± SD, *n* = 3 replicate samples from three independent experiments) from LDPE **(2A)**, PP **(2B)**, and PS **(2C)** after 2 h exposure to different pH, temperature and nutrient availability.

Screening of different temperature, pH, and nutrient exposures revealed changes in *V. parahaemolyticus* biofilm biomass, cell dispersal concentrations and biofilm viability (Figures 1,1A–3A; Supplementary Tables S1, S2, S10, S11). Exposure to elevated temperature (37°C) had variable effects on *V. parahaemolyticus* biofilm biomass, but increased cell dispersal concentrations in nutrient rich media (MSYE). However, in nutrient starved media (MS), exposure to elevated temperature appeared to increase biofilm biomass but decrease cell dispersal concentrations, except at lower pH levels of 3 and 4 where cell dispersal concentrations increased. Exposure to lower pHs (3, 4) led to a decrease in biofilm biomass in both nutrient rich and nutrient starved conditions, with a greater negative effect on cell dispersal concentrations in nutrient rich conditions (Figure 1,1A). ANOVA revealed certain significant differences (*p* ≤ 0.05) in the amount of *V. parahaemolyticus* biofilm biomass and cell dispersal concentrations in microplates. There was a significant positive effect of pH on *V. parahaemolyticus* overall biofilm biomass (*p* ≤ 0.05) and cell dispersal concentrations (*p* ≤ 0.001) in microplates (Supplementary Table S5). Meaning, when pH increased there was a significant increase in both *V. parahaemolyticus* biofilm biomass and cell dispersal concentrations in a short time (2 h). However, temperature and nutrient starvation were not significant factors on either biofilm biomass or cell dispersal concentrations (Supplementary Tables S3, S4). Biofilms of *V. parahaemolyticus* were able to tolerate and survive in both nutrient rich and starved conditions at pH 4–8.1 for 2 h, with similar metabolism characteristics across these conditions (Figures 1,2A,3A; Supplementary Tables S10, S11). Biofilm viability was impacted in pH of 3 and noticeably decreased, however *V. parahaemolyticus* biofilms still tolerated and survived a pH of 3 in both nutrient rich and starved conditions and biofilms recovered in growth once exposed to non-stressed conditions.

### Temperature, pH and nutrients affect *Vibrio vulnificus* in microplates

Screening different temperature, pH, and nutrient exposures revealed changes in *V. vulnificus* biofilm biomass, cell dispersal concentrations and biofilm viability (Figures 1,1B–3B; Supplementary Tables S1, S2, S10, S11). Exposure to elevated temperature (37°C) had variable effects on *V. vulnificus* biofilm biomass, but increased cell dispersal concentrations in nutrient rich media (MSYE). This same phenomenon was observed in nutrient starved media (MS), with variable effects on biofilm biomass, but increased cell dispersal. However, *V. vulnificus* was greatly affected in nutrient starved conditions, as this led to a decrease in biofilm biomass across all pHs at both 25 and 37°C compared to nutrient rich conditions. Exposure to lower pHs (3, 4) led to a greater decrease in biofilm biomass in nutrient rich conditions compared to nutrient starved (Figure 1,1B). However, elevated temperature, pH, and nutrient starvation were not significant factors in either biofilm biomass or cell dispersal concentrations in microplates (Supplementary Tables S3–S5). Biofilms of *V. vulnificus* were able to tolerate and survive in both nutrient rich and starved conditions at pH 4–8.1 for 2 h, with similar metabolism characteristics across these conditions (Figures 1,2B,3B; Supplementary Tables S10, S11). Biofilm viability was impacted in pH of 3 and noticeably decreased. *V. vulnificus* was able to survive pH of 3 in nutrient rich conditions but did not recover as well and did not survive pH of 3 in nutrient starved conditions.

### Temperature, pH and nutrients affect *Vibrio parahaemolyticus* on LDPE

Exposure to elevated temperature (37°C) had variable effects on *V. parahaemolyticus* biofilm biomass and biofilm cell concentrations on LDPE in nutrient rich media (MSYE) (Figure 2,1A; Supplementary Tables S12, S13). However, biofilm biomass on LDPE was greater in nutrient rich compared to nutrient starved media (MS) across all pH levels and elevated temperature. In nutrient starved conditions, exposure to elevated temperature appeared to have variable effects on LDPE biofilm biomass, but elevated temperature decreased biofilm cell concentrations across all pH levels. Cell dispersal concentrations from LDPE were roughly the same at medium to higher pH (5–8.1) both in nutrient rich and nutrient starved conditions (Figure 2,2A; Supplementary Tables S14, S15). While small concentrations of biofilm cells survived at the lowest pH of 3 in both nutrient rich and starved conditions, no dispersal cells were detected at pH 3. Cell colonization was noticeably higher in nutrient rich conditions compared to nutrient starved. Elevated temperature contributed to greater cell colonization in nutrient rich conditions but decreased cell colonization in nutrient starved. ANOVA revealed certain significant differences (*p* ≤ 0.05) in the amount of biofilm biomass on LDPE. There was a significant positive effect of pH (*p* ≤ 0.01) on *V. parahaemolyticus* biofilm biomass on LDPE, meaning when pH increased, biofilm biomass on LDPE significantly increased (Supplementary Table S26).

### Temperature, pH and nutrients affect *Vibrio parahaemolyticus* on PP

Elevated temperature largely decreased *V. parahaemolyticus* biofilm biomass (except at pH 3) and biofilm cell concentrations (except at pH 7) on PP in nutrient rich media (MSYE) (Figure 2,1B; Supplementary Tables S16, S17). In nutrient starved media (MS), exposure to elevated temperature appeared to have variable effects on PP biofilm biomass, with little changes in biofilm cell concentrations except slight decreases in cell concentrations at pH of 4 and 8.1. Cell dispersal concentrations from PP were roughly the same at higher pH (7–8.1), in that cell dispersal in nutrient rich conditions was slightly higher at both 25°C and 37°C compared to nutrient starved (Figure 2, 2B; Supplementary Tables S18, S19). Cell colonization was noticeably higher in nutrient rich conditions compared to nutrient starved, especially at elevated temperature (37°C) and at pH of 6–8.1. While small concentrations of biofilm cells survived at the lowest pH of 3 in nutrient rich conditions, no dispersal cells were detected at pH 3. ANOVA revealed certain significant differences (*p* ≤ 0.05) in the amount of cell dispersal and colonization from PP. There was a significant positive effect of pH (*p* ≤ 0.05) on *V. parahaemolyticus* cell dispersal from PP, meaning as pH increased, cell dispersal also significantly increased (Supplementary Table S26). There was also a significant positive effect of pH alone (*p* ≤ 0.01) and a significant synergistic positive effect of pH and elevated temperature (*p* ≤ 0.001) on *V. parahaemolyticus* cell colonization from dispersal of PP (Supplementary Tables S26, S28). This means when just pH and a combination of pH and temperature increased, cell colonization also significantly increased. However, a combination of pH, elevated temperature and nutrient starvation led to a significant synergistic negative effect (*p* ≤ 0.01) on *V. parahaemolyticus* cell colonization from dispersal of PP (Supplementary Table S30).

### Temperature, pH and nutrients affect *Vibrio parahaemolyticus* on PS

Elevated temperature decreased *V. parahaemolyticus* biofilm biomass but had variable effects on biofilm cell concentrations across all pH levels on PS in nutrient rich media (MSYE) (Figure 2,1C; Supplementary Tables S20, S21). In nutrient starved media (MS), exposure to elevated temperatures decreased biofilm biomass across all pH levels (except pH of 4), and decreased biofilm cell concentrations at pH 4–7. An increase in temperature increased cell dispersal concentrations from PS at pH 5–8.1 (Figure 2,2C; Supplementary Tables S22, S23). Cell colonization was noticeably higher in nutrient rich conditions compared to nutrient starved, especially at elevated temperature (37°C) and at pH 6–8.1. No concentrations of biofilm cells or dispersal cells were detected at pH 3. ANOVA revealed certain significant differences (*p* ≤ 0.05) in the amount of cell colonization from PS. There was a significant positive effect of pH alone (*p* ≤ 0.05) and a significant synergistic positive effect of pH and elevated temperature (*p* ≤ 0.01) on *V. parahaemolyticus* cell colonization from dispersal of PS (Supplementary Tables S26, S28). This means when just pH and a combination of pH and temperature increased, cell colonization also significantly increased. However, a combination of pH, elevated temperature and nutrient starvation led to a significant synergistic negative effect (*p* ≤ 0.05) on *V. parahaemolyticus* cell colonization from dispersal of PS (Supplementary Table S30).

### Temperature, pH and nutrients affect *Vibrio vulnificus* on LDPE

Exposure to elevated temperature (37°C) had variable effects on *V. vulnificus* biofilm biomass across all pH levels but decreased biofilm cell concentrations on LDPE in nutrient rich media (MSYE) at pH 6–8.1 (Figure 3,1A; Supplementary Tables S12, S13). In nutrient starved media (MS), exposure to elevated temperature increased biofilm biomass at pH 6–7.4 but had variable effects on biofilm cell concentrations on LDPE. An increase in temperature increased cell dispersal concentrations from LDPE at pH 5–8.1 in nutrient rich media (Figure 3,2A; Supplementary Tables S14, S15). While elevated temperature had variable effects on cell colonization from LDPE in nutrient rich media, colonization was noticeably higher compared to nutrient starved conditions. No concentrations of biofilm cells or dispersal cells were detected at pH 3. ANOVA revealed certain significant differences (*p* ≤ 0.05) in the amount of cell colonization from LDPE. There was a significant positive effect of pH on *V. vulnificus* cell colonization from LDPE (*p* ≤ 0.05) (Supplementary Table S26). This means as pH increased, there was a significant increase in the amount of cell colonization from LDPE.

### Temperature, pH and nutrients affect *Vibrio vulnificus* on PP

Exposure to elevated temperature (37°C) increased *V. vulnificus* biofilm biomass at pH 6–7.4 but had variable effects on biofilm cell concentrations on PP in nutrient rich media (MSYE) (Figure 3,1B; Supplementary Tables S16, S17). Biofilm biomass on PP was greater in nutrient rich compared to nutrient starved media (MS) across all pH levels. In nutrient starved conditions, exposure to elevated temperature had variable effects on both biofilm biomass and cell concentrations. However, at pH of 4 biofilm cell concentrations were noticeably higher at 25°C and especially 37°C in nutrient starved conditions compared to nutrient rich. An increase in temperature increased cell dispersal concentrations from PP at pH 6–8.1 and increased cell colonization from PP at pH 5–7.4 in nutrient rich media (Figure 3,2B; Supplementary Tables S18, S19). Cell colonization was noticeably higher in nutrient rich conditions compared to nutrient starved, especially at elevated temperature (37°C) and at pH 6–7.4. However, at pH of 4 cell dispersal were noticeably higher at 25°C and especially 37°C in nutrient starved conditions compared to nutrient rich. No concentrations of biofilm cells or dispersal cells were detected at pH 3. ANOVA revealed certain significant differences (*p* ≤ 0.05) in the amount of cell colonization from PP. There was a significant positive effect of pH on *V. vulnificus* cell colonization from PP (*p* ≤ 0.05) (Supplementary Table S26). This means as pH increased, there was a significant increase in the amount of cell colonization from PP.

### Temperature, pH and nutrients affect *Vibrio vulnificus* on PS

Exposure to elevated temperature (37°C) had variable effects on *V. vulnificus* biofilm biomass across all pH levels, but increased cell biofilm concentrations on PS in nutrient rich media (MSYE) at pH 5–7.4 (Figure 3,1C; Supplementary Tables S20, S21). In nutrient starved media (MS), exposure to elevated temperature increased biofilm biomass at pH 3–7.4, especially at pH 5 and 6 as biofilm biomass was greater at these pH levels in nutrient starved conditions compared to nutrient rich. However, elevated temperature decreased biofilm cell concentrations at pH 5–7.4 in nutrient starved conditions. An increase in temperature increased cell dispersal concentrations from PS at pH 5–8.1, with noticeable increases in cell colonization at pH 6 and 7.4 in nutrient rich media (Figure 3,2C; Supplementary Tables S22, S23). Cell colonization was noticeably higher in most pH levels (except pH of 4 and 6) in nutrient rich conditions compared to nutrient starved. An increase in temperature decreased cell dispersal at pH 6–8.1 in nutrient starved media. No concentrations of biofilm cells or dispersal cells were detected at pH 3.

### Temperature and human media affect *Vibrio parahaemolyticus* in microplates

**Figure 4:**
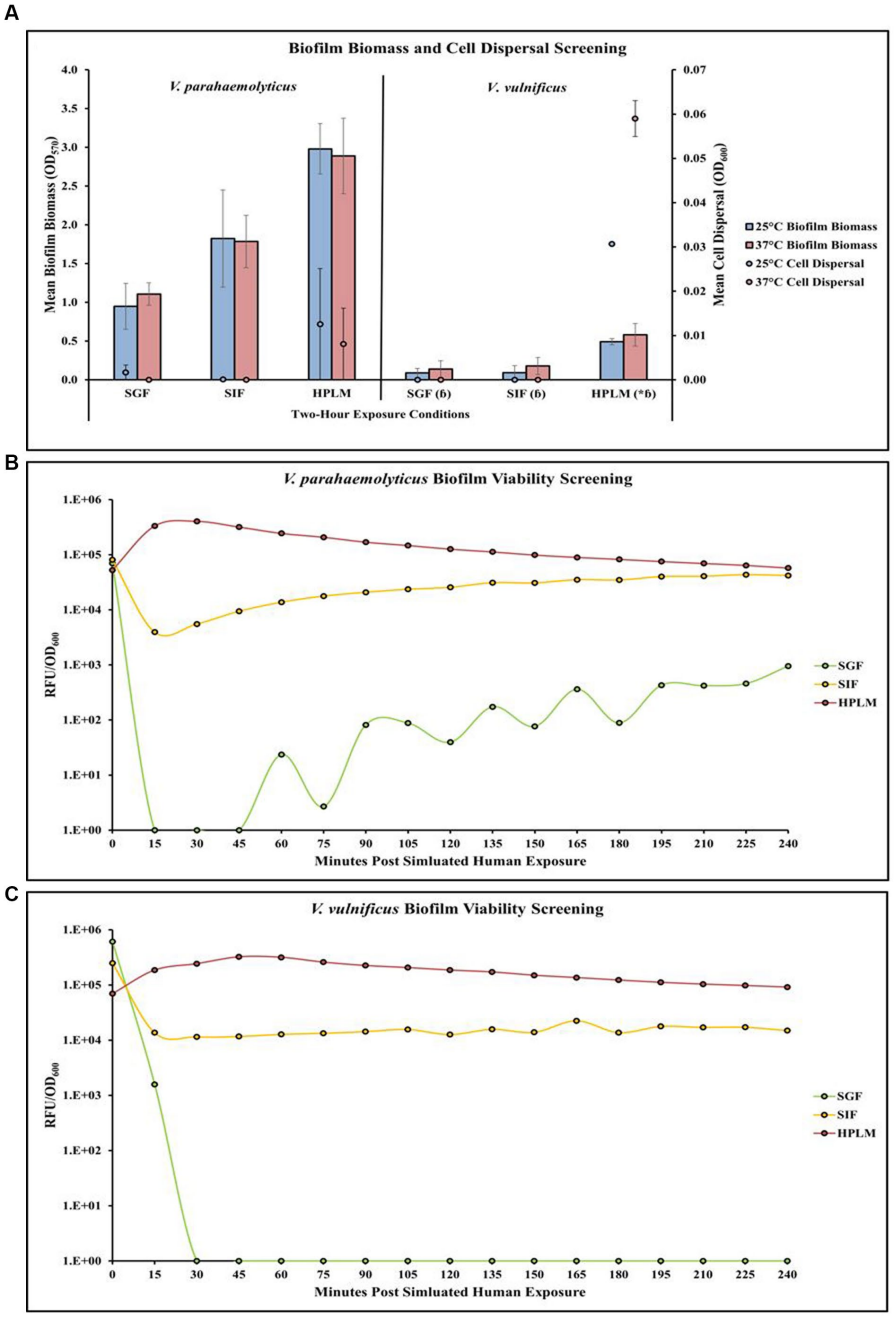
Exposure to different simulated human medias influences *V. parahaemolyticus* and *V. vulnificus* biofilm biomass, cell dispersal and viability in microplates. Comparison of overall biofilm biomass and cell dispersal **(A)** (values represent *X* ± SD, *n* = 3 replicate samples from three independent experiments) between different simulated human medias at different temperatures after 2 h exposure. Comparison of biofilm viability (RFU/OD_600_) of *V. parahaemolyticus* **(B)** and *V. vulnificus* **(C)** after 2 h exposure to simulated human medias at 37°C. * = significantly greater biofilm biomass compared to similar pH MSYE, ɓ = significantly less biofilm biomass compared to *V. parahaemolyticus* in same type of media.

Screening of different temperature and simulated human media exposures revealed changes in *V. parahaemolyticus* biofilm biomass, cell dispersal concentrations and biofilm viability (Figures 4A,B; Supplementary Tables S31, S32, S40). Exposure to elevated temperature (37°C) decreased *V. parahaemolyticus* biofilm biomass in SIF and HPLM, but increased biofilm biomass in SGF. Elevated temperature also decreased cell dispersal concentrations in HPLM. *V. parahaemolyticus* biofilm biomass and cell dispersal concentrations were greatest in HPLM across all three simulated human medias. Biofilm biomass was lowest after exposure to SGF, with cell dispersal concentrations being mostly undetected in SGF and SIF (Figure 4A). Results of ANOVAs revealed certain significant differences (*p* ≤ 0.05) in the amount of biofilm biomass concentrations in microplates. There were no significant effects of temperature on *V. parahaemolyticus* biofilm biomass or cell dispersal concentrations in SGF, SIF or HPLM (Supplementary Table S33). There were also no significant effects on media composition on *V. parahaemolyticus* biofilm biomass or cell dispersal compared to similar pH MSYE (Supplementary Table S34). Biofilms of *V. parahaemolyticus* were able to tolerate and survive in both HPLM and SIF for 2 h (Figure 4B; Supplementary Table S40). *V. parahaemolyticus* had similar metabolic characteristics after exposure to HPLM and SIF (Figure 4B). Biofilm viability was impacted in SGF and noticeably decreased. However, *V. parahaemolyticus* biofilms still tolerated and survived exposure to SGF and biofilms recovered in growth once exposed to non-stressed conditions.

### Temperature and human media affect *Vibrio vulnificus* in microplates

Screening of different temperature and simulated human media exposures revealed changes in *V. vulnificus* biofilm biomass, cell dispersal concentrations and biofilm viability (Figures 4A,C; Supplementary Tables S31, S32, S40). Exposure to elevated temperature (37°C) increased *V. vulnificus* biofilm biomass in SGF, SIF, and HPLM. Elevated temperature also increased cell dispersal concentrations in HPLM. *V. vulnificus* biofilm biomass and cell dispersal concentrations were greatest in HPLM across all three simulated human medias. Biofilm biomass was comparable after exposure to SGF and SIF, with cell dispersal concentrations being mostly undetected (Figure 4A). There were no significant effects of temperature on *V. vulnificus* biofilm biomass or cell dispersal concentrations in SGF, SIF or HPLM (Supplementary Table S33). However, HPLM significantly enhanced *V. vulnificus* biofilm biomass (*p* ≤ 0.05) compared to similar pH MSYE (Supplementary Table S34). There were no significant effects of SGF or SIF on biofilm biomass or cell dispersal concentrations compared to similar pH MSYE (Supplementary Table S34). *V. vulnificus* had significantly less biofilm biomass after exposure to SGF (*p* ≤ 0.01), SIF (*p* ≤ 0.01), and HPLM (*p* ≤ 0.01), compared to *V. parahaemolyticus* (Supplementary Table S36). Biofilms of *V. vulnificus* were able to tolerate and survive in both HPLM and SIF for 2 h (Figure 4C; Supplementary Table S40). *V. vulnificus* biofilm cell metabolism was higher after exposure to HPLM compared to SIF (Figure 4C). *V. vulnificus* was not able to survive exposure to SGF.

### Temperature and human media affect *Vibrio parahaemolyticus* on LDPE

**Figure 5:**
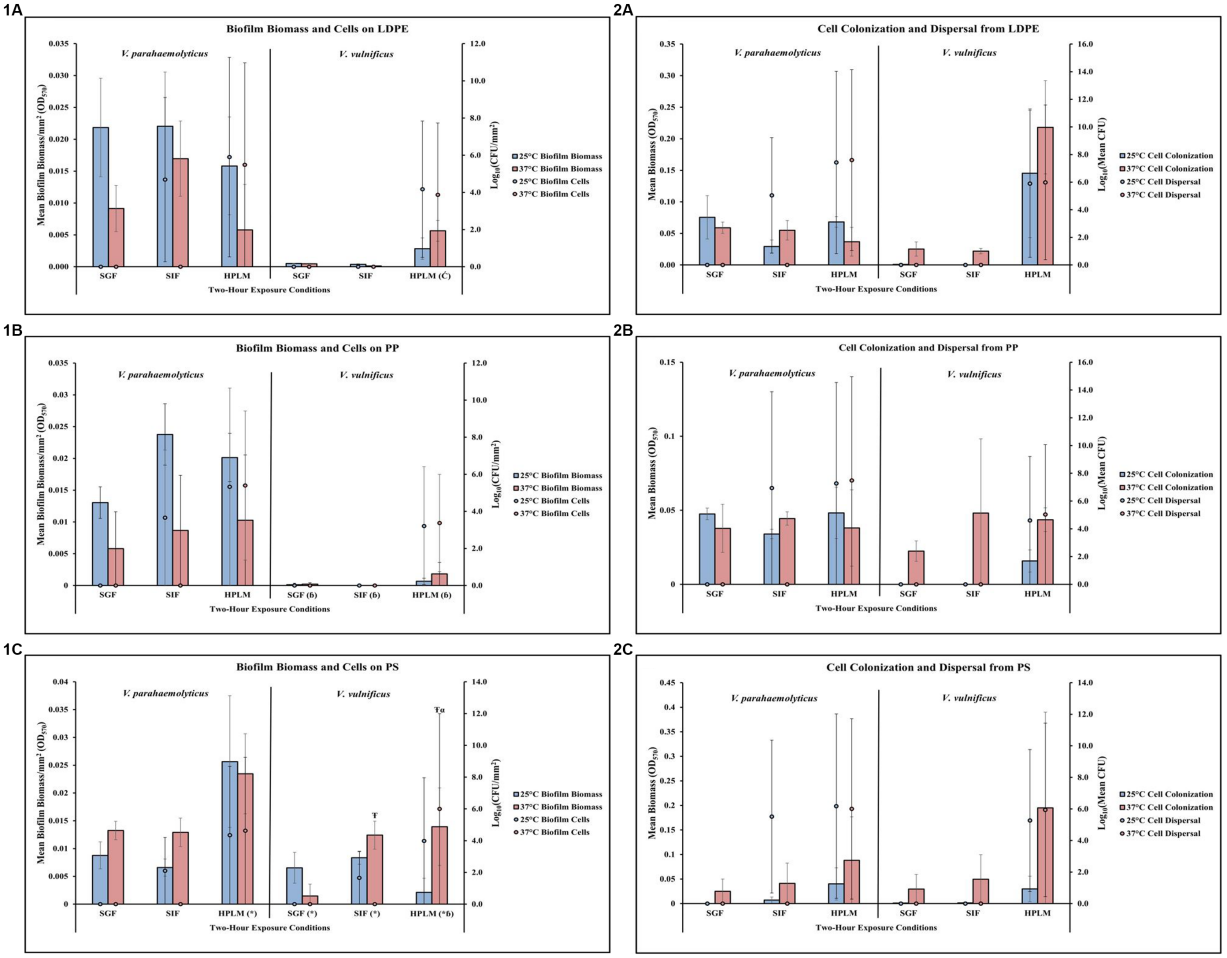
Exposure to different simulated human media influences *V. parahaemolyticus* and *V. vulnificus* biofilm processes on and from LDPE, PP, and PS MPs. Comparison of biofilm biomass and CFUs (values represent X ± SD, *n* = 3 replicate samples from three independent experiments) on LDPE **(1A)**, PP **(1B)**, and PS **(1C)** and subsequent biofilm cell dispersal CFUs and microplate colonization (values represent X ± SD, *n* = 3 replicate samples from three independent experiments) from LDPE **(2A)**, PP **(2B)**, and PS **(2C)** after 2-h exposure to different simulated human medias at different temperatures. * = significantly greater biofilm biomass compared to similar pH MSYE, Ć = significantly greater biofilm cell density compared to similar pH MSYE, ɓ = significantly less biofilm biomass compared to *V. parahaemolyticus* in same type of media, Ŧ = significantly greater biofilm biomass compared to 25°C, α = significantly greater biofilm cell density compared to 25°C.

Exposure to elevated temperature decreased *V. parahaemolyticus* biofilm biomass and biofilm cell concentrations on LDPE in SGF, SIF, and HPLM (Figure 5,1A; Supplementary Tables S41, S42). *V. parahaemolyticus* biofilm biomass on LDPE was greatest at both 25°C and 37°C in SIF compared to SGF and HPLM, but biofilm cell concentrations were greatest at both 25°C and 37°C in HPLM compared to SIF and SGF. Elevated temperature decreased *V. parahaemolyticus* cell dispersal concentrations from LDPE in SIF, but increased cell dispersal in HPLM (Figure 5,2A; Supplementary Tables S43, S44). This trend was opposite for cell colonization, as elevated temperature increased cell colonization from LDPE in SIF but decreased cell colonization in HPLM. While no concentrations of biofilm cells or dispersal cells were detected after exposure to SGF, biofilm biomass and cell colonization was higher at both 25°C and 37°C compared to HPLM.

### Temperature and human media affect *Vibrio vulnificus* on LDPE

Exposure to elevated temperature did not have noticeable effects on *V. vulnificus* biofilm biomass or biofilm cell concentrations on LDPE in SGF or SIF (Figure 5,1A; Supplementary Tables S41, S42). However, elevated temperature did increase biofilm biomass but slightly decreased biofilm cell concentrations in HPLM. Both biofilm biomass and biofilm cell concentrations were greatest in HPLM compared to SGF and SIF. Again, exposure to elevated temperature did not have detectable effects on *V. vulnificus* cell dispersal from LDPE in SGF or SIF (Figure 5,2A; Supplementary Tables S43, S44). However, elevated temperature did increase cell colonization from LDPE in SGF, SIF and HPLM, with slightly higher cell dispersal concentrations in HPLM. Both cell colonization and cell dispersal concentrations were greatest in HPLM compared to SGF and SIF. No detectable concentrations of *V. vulnificus* biofilm cells or dispersal cells were observed after exposures to SGF or SIF. ANOVA revealed certain significant differences (*p* ≤ 0.05) in the amount of *V. vulnificus* biofilm cell concentrations on LDPE. HPLM significantly enhanced *V. vulnificus* biofilm cell concentrations on LDPE (*p* ≤ 0.05) when compared to MSYE at a similar pH (Supplementary Table S53).

### Temperature and human media affect *Vibrio parahaemolyticus* on PP

Exposure to elevated temperature decreased *V. parahaemolyticus* biofilm biomass on PP in SGF, SIF, and HPLM (Figure 5,1B; Supplementary Tables S45, S46). *V. parahaemolyticus* biofilm biomass on PP was greatest at 25°C in SIF and 37°C in HPLM, but biofilm cell concentrations were greatest at both 25°C and 37°C in HPLM compared to SIF and SGF. An increase in temperature increased *V. parahaemolyticus* cell dispersal concentrations from PP in HPLM, and SIF but decreased cell dispersal in SIF (Figure 5,2B; Supplementary Tables S47, S48). This trend was opposite for cell colonization, as elevated temperature increased cell colonization from PP in SIF but decreased cell colonization in HPLM. While no concentrations of *V. parahaemolyticus* biofilm cells or dispersal cells were detected after exposure to SGF, cell colonization was about the same at both 25°C and 37°C compared to HPLM.

### Temperature and human media affect *Vibrio vulnificus* on PP

Exposure to elevated temperature did not have noticeable effects on *V. vulnificus* biofilm biomass or biofilm cell concentrations on PP in SGF or SIF (Figure 5,1B; Supplementary Tables S45, S46). However, elevated temperature did increase biofilm biomass and slightly increased biofilm cell concentrations in HPLM. Both biofilm biomass and biofilm cell concentrations were greatest in HPLM compared to SGF and SIF. Exposure to elevated temperature did not have noticeable effects on *V. vulnificus* cell dispersal from PP in SGF or SIF (Figure 5,2B; Supplementary Tables S47, S48). However, elevated temperature did increase cell colonization from PP in SGF, SIF and HPLM, with slightly greater cell dispersal concentrations in HPLM. Both cell colonization and cell dispersal concentrations were greatest in HPLM compared to SGF and SIF. No concentrations of *V. vulnificus* biofilm cells or dispersal cells were detected after exposure to SGF or SIF. ANOVA revealed certain significant differences (*p* ≤ 0.05) in the amount of *V. vulnificus* biofilm biomass on PP. *V. vulnificus* had significantly less biofilm biomass on PP after exposure to SGF (*p* ≤ 0.05), SIF (*p* ≤ 0.05), and HPLM (*p* ≤ 0.05), compared to *V. parahaemolyticus* (Supplementary Table S56).

### Temperature and human media affect *Vibrio parahaemolyticus* on PS

Exposure to elevated temperature increased *V. parahaemolyticus* biofilm biomass on PS in SGF and SIF, but decreased biofilm biomass in HPLM (Figure 5,1C; Supplementary Tables S49, S50). *V. parahaemolyticus* biofilm biomass and biofilm cell concentrations on PS were greatest at both 25°C and 37°C in HPLM compared to SIF and SGF. An increase in temperature decreased *V. parahaemolyticus* cell dispersal concentrations from PS in HPLM and SIF (Figure 5,2C; Supplementary Tables S51, S52). This trend was opposite for cell colonization, as elevated temperature increased cell colonization from PS in SIF and HPLM. While no concentrations of *V. parahaemolyticus* biofilm cells or dispersal cells were detected after exposure to SGF, cell colonization was about the same at both 25°C and 37°C compared to SIF. ANOVA revealed certain significant differences (*p* ≤ 0.05) in the amount of *V. parahaemolyticus* biofilm biomass on PS. HPLM significantly enhanced *V. parahaemolyticus* biofilm biomass on PS (≤0.01) compared to MSYE with similar pH (Supplementary Table S53).

### Temperature and human media affect *Vibrio vulnificus* on PS

Exposure to elevated temperature increased *V. vulnificus* biofilm biomass on PS in SIF and HPLM, but decreased biofilm biomass in SGF (Figure 5,1C; Supplementary Tables S49, S50). An increase in temperature increased biofilm cell concentrations in HPLM but decreased biofilm cell concentrations in SIF. Both biofilm biomass and biofilm cell concentrations were greatest in HPLM at elevated temperature compared to SGF and SIF. Exposure to elevated temperature did not have noticeable effects on *V. vulnificus* cell dispersal from PS in SGF or SIF (Figure 5,2C; Supplementary Tables S51, S52). However, elevated temperature did increase cell colonization from PS in SGF, SIF and HPLM, with slightly greater cell dispersal concentrations in HPLM. Both cell colonization and cell dispersal concentrations were greatest in HPLM compared to SGF and SIF. No concentrations of *V. vulnificus* biofilm cells or dispersal cells were detected after exposure to SGF or SIF. ANOVA revealed certain significant differences (*p* ≤ 0.05) in the amount of *V. vulnificus* biofilm biomass and biofilm cells on PS. HPLM (*p* ≤ 0.05), SGF (*p* ≤ 0.01) and SIF (≤0.01) significantly enhanced *V. vulnificus* biofilm biomass on PS compared to MSYE with similar pHs (Supplementary Table S53). Exposure to elevated temperature also significantly enhanced *V. vulnificus* biofilm biomass (*p* ≤ 0.001) and biofilm cells (≤0.01) on PS in HPLM and significantly enhanced biofilm biomass on PS in SIF (*p* ≤ 0.05) compared to MSYE of similar pHs (Supplementary Table S55). *V. vulnificus* had significantly less biofilm biomass on PS after exposure to HPLM (*p* ≤ 0.001) compared to *V. parahaemolyticus* (Supplementary Table S58). There was also a significant synergistic effect of HPLM and elevated temperature on *V. vulnificus* biofilm biomass and cell concentrations (*p* ≤ 0.05) on PS compared to *V. parahaemolyticus* (Supplementary Table S59).

### Media type affects *Vibrio parahaemolyticus* c-di-GMP production

**Figure 6:**
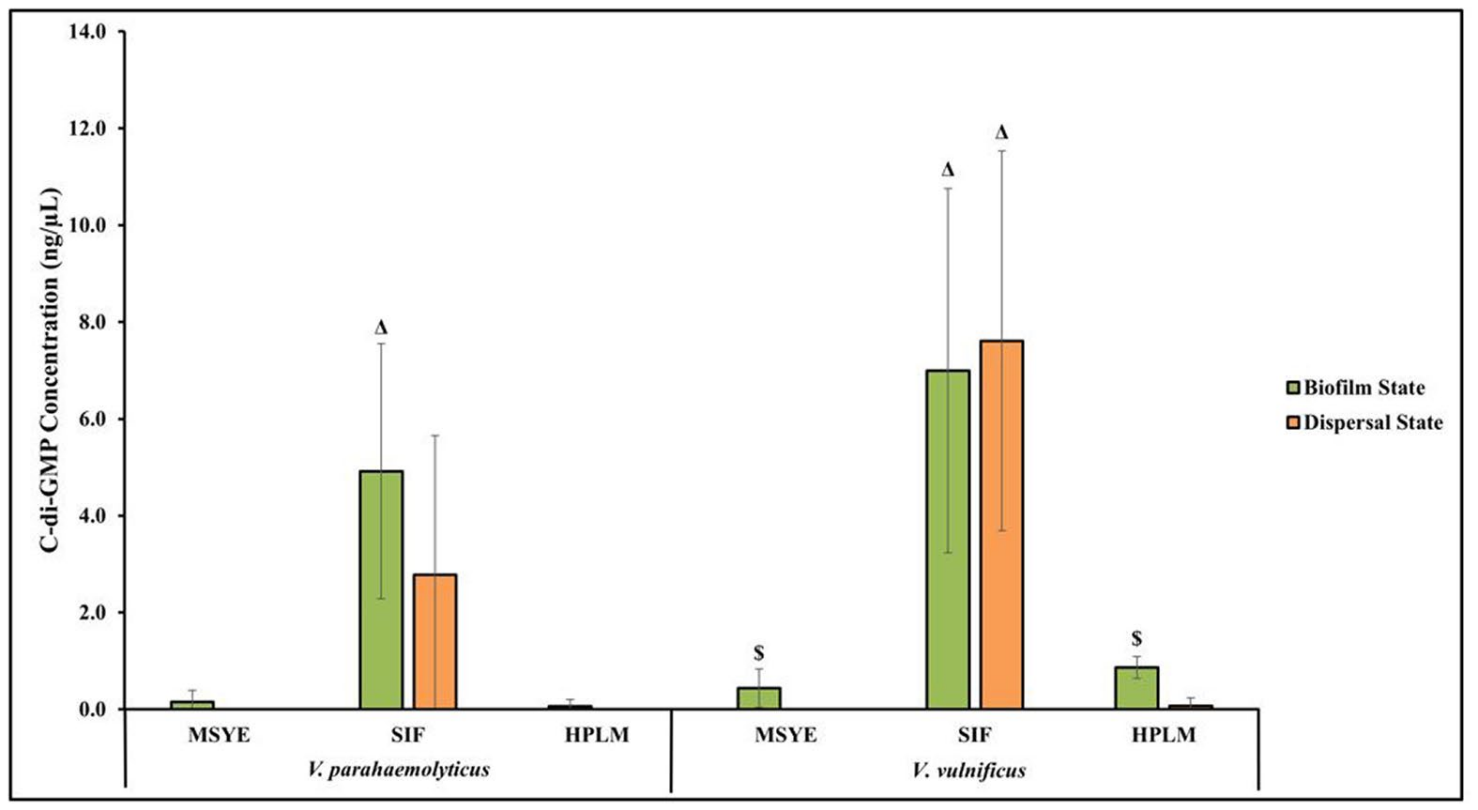
Exposure to different media composition and cell state influences *V. parahaemolyticus* and *V. vulnificus* c-di-GMP concentrations. Comparison of estimated c-di-GMP concentrations (values represent *X* ± SD, *n* = 3 replicate samples from three independent experiments) producd by *V. parahaemolyticus* and *V. vulnificus* biofilm and dispersed cells after 2 h exposure to MSYE, SIF, and HPLM at 37°C. Δ = significantly greater c-di-GMP concentration compared to MSYE and HPLM, $ = significantly greater c-di-GMP concentration compared to dispersal state in same media.

*V. parahaemolyticus* appeared to have differences in c-di-GMP concentrations in both its biofilm and dispersal states after being exposed to MSYE, SIF and HPLM (Figure 6; Supplementary Table S60). There were greater c-di-GMP concentrations in *V. parahaemolyticus* biofilm state in MSYE, SIF and HPLM compared to dispersal state. Exposure to SIF led to greater c-di-GMP concentrations in both biofilm and dispersal states compared to exposure to MSYE and HPLM. ANOVA revealed certain significant differences (*p* ≤ 0.05) in the concentrations of *V. parahaemolyticus* c-di-GMP concentrations between different media exposures. *V. parahaemolyticus* biofilm cells exposed to SIF had significantly enhanced c-di-GMP concentrations compared to biofilms exposed to MSYE (*p* ≤ 0.01) and HPLM *p* (≤ 0.01) (Supplementary Table S61). There were no significant differences in *V. parahaemolyticus* dispersal cell c-di-GMP concentrations in different medias (Supplementary Table S61). There were also no significant differences between the biofilm and dispersal state of *V. parahaemolyticus* cells after exposure to MSYE, SIF, and HPLM (Supplementary Table S62).

### Media type affects *Vibrio vulnificus* c-di-GMP production

*V. vulnificus* appeared to have differences in c-di-GMP concentrations in both its biofilm and dispersal states after being exposed to MSYE, SIF, and HPLM (Figure 6; Supplementary Table S60). There were greater c-di-GMP concentrations in biofilm cells exposed to MSYE and HPLM. However, exposure to SIF led to greater concentrations of c-di-GMP in dispersal cells compared to biofilm cells. Exposure to SIF led to greater c-di-GMP concentrations in both biofilm and dispersal states compared to exposure to MSYE and HPLM. ANOVA revealed certain significant differences (*p* ≤ 0.05) in the concentrations of *V. vulnificus* c-di-GMP concentrations between different media exposures and between biofilm and dispersal cell states. *V. vulnificus* biofilm and dispersal cells exposed to SIF had significantly enhanced c-di-GMP concentrations compared to their counterparts exposed to MSYE (*p* ≤ 0.01) and HPLM (*p* ≤ 0.01) (Supplementary Table S61). *V. vulnificus* biofilm cells also had significantly greater c-di-GMP concentrations compared to dispersal cells in MSYE (*p* ≤ 0.05) and HPLM (*p* ≤ 0.001), but there was no significant difference in biofilm and dispersal cell states when exposed to SIF (Supplementary Table S62).

## Discussion

Biofilm formation facilitates the ability of pathogenic bacteria like *Vibrio* to colonize most environmental niches like MPs. While *Vibrio* have been found to be a major community member on marine MPs, it is important to note that *Vibrio* concentrations are lower on these surfaces compared to natural marine particles (Bryant et al., 2016; Curren and Leong, 2019; Amaral-Zettler et al., 2020). However, MPs can still enable the bacteria embedded in the biofilm to be translocated to marine animals, and then vectored to humans due to biofilm dispersal from MPs to animal tissues or the biofilms on MPs themselves by ingestion (Austin, 2010; Kaplan, 2010; Yang et al., 2020; Bowley et al., 2021; Fabra et al., 2021). MPs’ role as substrates for microbial habitats and subsequently vectors of pathogenic *Vibrio* to humans must be further evaluated, especially during the human ingestion process (Zettler et al., 2013; Kirstein et al., 2016). To better understand the emerging public health risks associated with bacterial dispersal from plastic particles, studies are needed to determine how this process on different substrate types is affected by human environmental conditions, such as simulated human body pH, temperature, nutrient availability, and fluid composition. This study focused on how both *V. parahaemolyticus* and *V. vulnificus* disperse from common marine plastics, such as low-density polyethylene, polypropylene, and polystyrene, under different simulated human body conditions, especially those related to digestion.

Conditions likely to be encountered by *V. parahaemolyticus* and *V. vulnificus* biofilms on MPs as they transition to the human intestinal environment by ingestion include increases in temperature, decreases in pH and nutrient availability, and changes in fluid composition. *Vibrio* must detect and respond to changes in their environment to successfully survive and colonize their host (Gode-Potratz et al., 2011; Billaud et al., 2022). Our study supports this as both species rapidly responded to changes in temperature, pH, nutrient availability, and fluid composition as evident by changes in their biofilm biomass and cell dispersal from MPs and subsequent microplate surface colonization. Several studies have shown that sudden changes in nutrient availability can induce biofilm dispersal (Hunt et al., 2004; Sauer et al., 2004; Petrova and Sauer, 2016). Our study further suggests this as adding or removing the carbon and nitrogen sources (here, peptone and yeast extract) resulted in specific biofilm dispersal responses, which is also in accordance with Singh et al. (2017) who found a similar response in *V. cholerae*. In our study, *V. vulnificus* was more sensitive to nutrient starvation when compared to *V. parahaemolyticus*. A strong dispersal response occurred in *V. vulnificus* as determined by changes in its overall biofilm biomass, and specific biofilm biomasses on LDPE and PP when exposed to nutrient starved conditions. Only *V. parahaemolyticus* biofilms showed a strong dispersal response from LDPE surfaces following nutrient starvation conditions. These dispersal responses likely occurred as cells embedded in the biofilm detached to find other suitable surfaces to colonize to repeat their lifecycle, or to escape stressful nutrient scarce conditions (Kaplan, 2010; Barraud et al., 2015). Biofilm biomass, biofilm cell viability and cell dispersal of both *V. parahaemolyticus* and *V. vulnificus* decreased at lower pHs (pH 3, 4) on MPs. This is in accordance with Koo et al. (2000) and Wang et al. (2019) as they also found pH levels <4 led to a decrease in cell viability in both *V. parahaemolyticus* and *V. vulnificus*. While *V. parahaemolyticus* did have decreased biofilm cell viability and cell dispersal at acidic (pH = 4) conditions compared to higher pHs, it was still able to tolerate this acidic pH for two hours, which was consistent with results of Wang et al. (2019, 2020). There was also a decrease in biofilm cell viability and cell dispersal of both species after exposure to SGF, which more closely resembles the gastric liquid composition in the stomach’s environment. This suggests that acidic pH that closely resembles the stomach environment is effective in eradicating viable and pathogenic *Vibrio* on MPs. While the acidic conditions that resemble the human digestive system, especially those found in the stomach, seem to do an effective job in killing viable *Vibrio* cells, it is important to note that *Vibrio* are likely co-ingested with contaminated seafood, which alters the environmental conditions during digestion.

*Vibrio* biofilms on MPs are likely to be co-ingested with seafood, and since seafood consumption temporarily and significantly increases the pH level of gastric fluid in the stomach, this consequently can provide a protective effect and enable *V. parahaemolyticus* and *V. vulnificus* to survive exposure to these harsh digestion conditions (Conway et al., 1987; Koo et al., 2000; Wang et al., 2019; Çam and Brinkmeyer, 2020). As conditions become more favorable and optimal in the small intestine for both species, *Vibrio* may potentially leave their protective biofilm bunker on MPs and disperse and subsequently colonize the human intestinal tract (Tang et al., 2015). Our study supports this as higher pHs like those found in the small intestine led to higher cell dispersal from MPs and subsequent microplate colonization of both species, signifying that potentially ingested *Vibrio* on MPs can adequately disperse and potentially colonize the surfaces of the intestinal tract. This is further supported by an increase in biofilm cell viability and cell dispersal of both species after exposure to SIF, which closely resembles the intestinal fluid composition found in the intestinal environment.

The *Vibrio* species and strains’ planktonic cells in this study have been found to have hydrophobic properties in a previous study (Leighton et al., 2023). The hydrophobic nature of the bacterial cell surface in its planktonic lifestyle can greatly affect its later biofilm lifestyle on abiotic, hydrophobic surfaces like plastics (Rosenberg, 1984; Reifsteck et al., 1987). *Vibrio*’s development of specific adaptive mechanisms to the toxicity and low bioavailability of these specific plastic substrates might lead to changes in cell surface hydrophobicity (Krasowska and Sigler, 2014). Cell surface changes caused by plastic colonization may lead to potential host colonization priming, as it has been reported that adequate hydrophobic/hydrophilic properties of bacteria can contribute to colonization of the mucosal membranes in the small intestine and lead to human infection (Qin et al., 2008; Krasowska and Sigler, 2014; de Wouters et al., 2015). These changes in hydrophobicity due to initial MP colonization may explain the rapid colonization of microplates from dispersed *Vibrio* cells from MPs, especially at elevated temperature that resembled the internal human environment. This rapid microplate colonization may also be due to dispersal cells having short term increased motility and adherence phenotypes compared to their biofilm and planktonic counterparts (Chua et al., 2014; Uppuluri et al., 2018; Rumbaugh and Sauer, 2020). Cell motility must be considered in future *Vibrio* dispersal studies, as it is also a key factor in dispersion processes due to flagellar-mediated motility enabling cells that have dispersed from the biofilm to swim and colonize new surfaces (Satish et al., 2017; Teschler et al., 2022).

In our study, *V. parahaemolyticus* was previously isolated from the human enteric system, so this strain has likely adapted to survive and colonize the intestinal tract. Our study supports this as it did show resistance to conditions that resemble the human gastrointestinal environment such as in SGF and SIF at 37°C. Interestingly, it also showed greater resistance to these conditions compared to *V. vulnificus*, which was isolated from blood. This is further supported by the resazurin metabolic assay which revealed that *V. parahaemolyticus* biofilm cells were able to survive exposure to media with low pH (3) and in SGF which had a pH of 1.5 for 2 h, but *V. vulnificus* did not recover as well or survive. However, this survival could be due to the presence of persister cells, as these cells were able to survive the stressors, and once the stressor was removed, were able to become metabolically active again and the biofilms recover in growth and development (Xiong et al., 2022). *V. parahaemolyticus*, while not being isolated from blood, still had high amounts of biofilm biomass, biofilm cell concentrations, cell colonization and dispersal in HPLM at 25°C and 37°C. HPLM contains all the amino acids, vitamins, salts, sugars, and acids found in actual human blood, but without the immune response cells. Our study confirms HPLM might be a better alternative in human exposure studies and models compared to traditionally used media.

Also to be noted is that in our study, one of our *V. vulnificus* isolates, that was from human blood, has likely adapted to colonize wounds and blood serum (Çam and Brinkmeyer, 2020). Our study supports this as exposure to HPLM increased biofilm biomass and cell viability, especially at human body temperature (37°C). This further confirms that HPLM represents a favorable media for growth and exposure studies with human bloodborne pathogens compared to conventional media currently used to resemble pathogen responses in human systems. *V. vulnificus* at 37°C produced more biofilm, had greater concentrations of biofilm cells, greater colonization, and cell dispersal concentrations on and from every plastic type in HPLM in almost every exposure scenario compared to *V. parahaemolyticus*, which was isolated from the human enteric system. In some cases, rapid changes in temperature from 25°C to 37°C also contributed to increases in biofilm biomass and cell colonization by *V. vulnificus* on LDPE, PP and PS in simulated human media. This suggests that *V. vulnificus* may produce greater amounts of biofilm on MPs as a survival mechanism in response to higher temperatures in the human environment.

C-di-GMP signaling and concentration levels within cells can fluctuate in response to environmental cues, specifically those related to human host stressors (Koestler and Waters, 2014; Chua et al., 2015; Khan et al., 2016). C-di-GMP concentration levels dictate different mechanistic actions, as high concentrations lead to biofilm formation and low concentrations lead to dispersion (Valentini and Filloux, 2016; Andersen et al., 2021). Our study supports both the environment and cell state having impacts on c-di-GMP production, as both *V. parahaemolyticus* and *V. vulnificus* had higher levels of c-di-GMP in the biofilm state compared to dispersal state after MSYE and HPLM exposure at 37°C. Both species had high viability and biofilm biomass and cell concentrations in these medias, and coincidently lower c-di-GMP levels compared to SIF. Both species produced significantly more c-di-GMP in SIF in the biofilm state compared to HPLM and MSYE. Both species also had high levels of c-di-GMP in the dispersal state after exposure to SIF, potentially signifying a stress response in both biofilm and dispersal states. Compared to MSYE and HPLM, SIF contains bile salts, which have been found to induce stress and increase c-di-GMP concentrations in *V. cholerae*, another intestinal pathogen of the same genus (Koestler and Waters, 2014). Our study supports this notion that stressors related to digestion (i.e., bile salts) may impact and increase intracellular c-di-GMP concentrations in *Vibrio*. Intestinal pathogens like *V. parahaemolyticus* and *V. vulnificus* may also experience changes in temperature and nutrient starvation in the digestive system as they transition environments by ingestion (Hooper et al., 2002; Purcell et al., 2017). In our study, only conditions resembling the internal human environment (37°C, SIF, HPLM) were chosen to compare c-di-GMP levels, especially to conventional media (MSYE). However, as temperature and nutrient starvation have been found and speculated to impact intracellular c-di-GMP levels concentrations in these species, further studies are needed to confirm this after exposure to elevated temperature under these same types of conditions (Gjermansen et al., 2010; Bharati et al., 2012; Townsley and Yildiz, 2015; Song et al., 2017; Chodur Daniel et al., 2018).

Strain isolation source matters and is crucial when examining specific human exposure responses like in this study, but it is also important to note that these are two different *Vibrio* species, and these specific strains have different biofilm forming behaviors. While environmental exposure time was capped after two hours to better reflect human digestion processes and to target dispersed rather than planktonic cells, the exposure time to these environmental factors likely had major impact on biofilm dispersal, and longer or shorter exposure times to the same or different environmental conditions tested may lead to different biofilm responses (Marks et al., 2013; Barraud et al., 2014; Singh et al., 2017; Nair et al., 2021). Temperature was found to not be a major factor in influencing biofilm biomass in comparing MSYE and MS. This likely is due to the short exposure time of two hours and the media was not prewarmed at 37°C prior to exposure. There were also little significant differences in cell concentrations, cell colonization and cell dispersal on and from plastics when exposed to SGF, SIF, and HPLM compared to MSYE with similar pHs. This could mean exposure to pH, rather than the media composition, is more of a major determining factor of these biofilm processes on these surfaces.

These results indicate that human pathogenic strains of *V. parahaemolyticus* and *V. vulnificus* can rapidly respond to different marine and simulated human environmental conditions by dispersing high viable cell concentrations from biofilms on different MP material types *in vitro*. However, this dispersal process is highly variable, and while natural variability exists in nature from species to species, strain to strain, and even cell to cell, the variability observed in this study also depended on plastic type and environmental exposures like pH, temperature, nutrient availability, and media composition. Further studies are needed to examine these species and strains’ cell motility under simulated human conditions, as cell motility can also be a key factor in dispersion, subsequent surface attachment and thus host colonization (Satish et al., 2017). Further studies are also needed to compare these human isolated *Vibrio* strains biofilm dispersal processes to marine animal and environmentally isolated strains. Comparison of these *Vibrio in vitro* plastic dispersal processes under simulated human conditions to those found more closely in the natural human environment, which a continuous model system would provide with consecutive exposures to different elements of the human digestive environment are also needed (Wang et al., 2019). Further studies are needed to examine bacterial response on MPs in consumed seafood to better predict exposure risk. Also, as *Vibrio* are in close proximity to each other on MPs, there is a high chance of horizontal gene transfer, which could transfer antibiotic resistance and virulence genes, so examining transcriptomic profiles of these bacteria attached to MPs after exposure to human environmental cues will provide more information on potential virulence and antibiotic resistance gene regulation (Hu et al., 2022). Finally, further studies are needed to examine the interactions of *Vibrio* on MPs with the human intestinal environment and surface, as it has been shown that the presence of MPs and pathogens both separately and together in marine animal intestinal systems can change and damage gut microbiota and intestinal mucosa (Kamada et al., 2013; Yan et al., 2021; Hu et al., 2022). The interactions of *Vibrio* species with plastic surfaces have been shown to be quite strong but also complex in nature. This complexity likely enhances survival across many microenvironmental niches, and accounts for their growing persistence in response to climate change in coastal ocean systems. The emerging risks associated with pathogenic bacteria living on MPs must be further evaluated to protect One Health, especially in a changing climate.

## Data availability statement

The original contributions presented in the study are included in the article/Supplementary material, further inquiries can be directed to the corresponding author.

## Author contributions

RL, AD, and RN conceived and designed the study, analyzed the data, corrected the draft, built the final version of the manuscript, and read and approved the submitted version. RL, GKA, and GMA performed the lab experiments. LX and GC performed statistical analyses. RL wrote the first draft of the manuscript. All authors contributed to the article and approved the submitted version.
