# Supplementary material for: Vibrio parahaemolyticus and Vibrio vulnificus in vitro biofilm dispersal from microplastics influenced by simulated human environment
Source: Front Microbiol. 2023 Oct 3;14:1236471. doi: 10.3389/fmicb.2023.1236471 (PMC10579612; doi:10.3389/fmicb.2023.1236471)
Supplement: Supplementary file 1 [file Data_Sheet_1.docx]

Supplementary Material

***Vibrio parahaemolyticus* and *Vibrio vulnificus* *In Vitro* Biofilm Dispersal from Microplastics Influenced by Simulated Human Environmental Factors**

Ryan E. Leighton^1,2^, Liyan Xiong^1^, Gracie K. Anderson^1^, Grace Astarita^1^, Guoshuai Cai^1^, R. Sean Norman, Alan W. Decho^1,2*^

^1^Department of Environmental Health Sciences, University of South Carolina, Columbia, SC, United States

^2^Department of Environmental Health Sciences, NIEHS Center for Oceans and Human Health and Climate Change Interactions, University of South Carolina, Columbia, SC, United States

*** Correspondence: Alan Decho**Corresponding Author
Awdecho@mailbox.sc.edu

# Supplementary Data

# Table S1. Effect of pH, temperature, and nutrient availability on mean overall biofilm biomass^1^ in microplates by *V. parahaemolyticus* and *V. vulnificus*.

| \| **Species** \| **pH** \| **Nutrient Rich (25°C)**  **(± SD)** \| **Nutrient Rich (37°C)**  **(± SD)** \| **Nutrient Starved (25°C) (± SD)** \| **Nutrient Starved (37°C) (± SD)** \| \| --- \| --- \| --- \| --- \| --- \| --- \| \| *V. parahaemolyticus** \| 3 \| 0.95  0.29 \| 1.11  0.14 \| 0.81  0.13 \| 1.13  0.13 \| \|  \| 4 \| 1.14  0.36 \| 1.01  0.23 \| 1.04  0.12 \| 1.34  0.11 \| \|  \| 5 \| 1.68  0.17 \| 1.57  0.23 \| 1.50  0.17 \| 1.74  0.04 \| \|  \| 6 \| 1.69  0.08 \| 1.81  0.37 \| 1.40  0.22 \| 1.49  0.10 \| \|  \| 7 \| 1.82  0.62 \| 1.78  0.33 \| 1.30  0.15 \| 1.60  0.13 \| \|  \| 7.4 \| 1.76  0.25 \| 1.75  0.55 \| 1.15  0.02 \| 1.53  0.08 \| \|  \| 8.1 \| 1.55  0.40 \| 1.65  0.40 \| 1.29  0.15 \| 1.60  0.20 \| \| *V. vulnificus* \| 3 \| 0.09  0.05 \| 0.14  0.10 \| 0.06  0.00 \| 0.02  0.00 \| \|  \| 4 \| 0.08  0.05 \| 0.10  0.09 \| 0.03  0.00 \| 0.02  0.00 \| \|  \| 5 \| 0.26  0.13 \| 0.17  0.053 \| 0.03  0.01 \| 0.04  0.01 \| \|  \| 6 \| 0.23  0.110 \| 0.24  0.044 \| 0.03  0.002 \| 0.04  0.02 \| \|  \| 7 \| 0.09  0.09 \| 0.18  0.11 \| 0.06  0.02 \| 0.05  0.03 \| \|  \| 7.4 \| 0.16  0.02 \| 0.26  0.06 \| 0.04  0.01 \| 0.05  0.01 \| \|  \| 8.1 \| 0.23  0.03 \| 0.22  0.08 \| 0.07  0.01 \| 0.07  0.00 \| \|  \|  \|  \|  \|  \|  \| |
| --- | --- | --- | --- | --- | --- | --- | --- | --- | --- | --- | --- | --- | --- | --- | --- | --- | --- | --- | --- | --- | --- | --- | --- | --- | --- | --- | --- | --- | --- | --- | --- | --- | --- | --- | --- | --- | --- | --- | --- | --- | --- | --- | --- | --- | --- | --- | --- | --- | --- | --- | --- | --- | --- | --- | --- | --- | --- | --- | --- | --- | --- | --- | --- | --- | --- | --- | --- | --- | --- | --- | --- | --- | --- | --- | --- | --- | --- | --- | --- | --- | --- | --- | --- | --- | --- | --- | --- | --- | --- | --- | --- | --- | --- | --- | --- | --- |

^1^Optical density at 570 nm

(± SD) Standard deviation

(*) Significant (+) effect of pH on biofilm biomass

Table S2. Effect of pH, temperature, and nutrient availability on mean overall final^1^ *V. parahaemolyticus* and *V. vulnificus* dispersed cells^2^.

| \| **Species** \| **pH** \| **Nutrient Rich (25°C)**  **(± SD)** \| **Nutrient Rich (37°C)**  **(± SD)** \| **Nutrient Starved (25°C) (± SD)** \| **Nutrient Starved (37°C) (± SD)** \| \| --- \| --- \| --- \| --- \| --- \| --- \| \| *V. parahaemolyticus** \| 3 \| 0.001  0.001 \| 0.012  0.002 \| 0.000  0.000 \| 0.009  0.001 \| \|  \| 4 \| 0.000  0.001 \| 0.005  0.003 \| 0.007  0.005 \| 0.010  0.003 \| \|  \| 5 \| 0.054  0.001 \| 0.092  0.007 \| 0.012  0.010 \| 0.011  0.003 \| \|  \| 6 \| 0.065  0.004 \| 0.088  0.009 \| 0.013  0.004 \| 0.012  0.005 \| \|  \| 7 \| 0.065  0.005 \| 0.105  0.005 \| 0.012  0.003 \| 0.009  0.004 \| \|  \| 7.4 \| 0.069  0.003 \| 0.087  0.012 \| 0.014  0.004 \| 0.008  0.007 \| \|  \| 8.1 \| 0.083  0.003 \| 0.087  0.006 \| 0.018  0.018 \| 0.008  0.008 \| \| *V. vulnificus* \| 3 \| 0.000  0.000 \| 0.007  0.005 \| 0.000  0.000 \| 0.015  0.002 \| \|  \| 4 \| 0.000  0.000 \| 0.001  0.002 \| 0.000  0.000 \| 0.011  0.003 \| \|  \| 5 \| 0.035  0.005 \| 0.093  0.004 \| 0.000  0.000 \| 0.010  0.004 \| \|  \| 6 \| 0.048  0.005 \| 0.101  0.005 \| 0.000  0.000 \| 0.004  0.002 \| \|  \| 7 \| 0.048  0.004 \| 0.107  0.006 \| 0.000  0.000 \| 0.012  0.002 \| \|  \| 7.4 \| 0.049  0.002 \| 0.111  0.001 \| 0.000  0.000 \| 0.010  0.003 \| \|  \| 8.1 \| 0.045  0.005 \| 0.081  0.001 \| 0.000  0.000 \| 0.011  0.004 \| |
| --- | --- | --- | --- | --- | --- | --- | --- | --- | --- | --- | --- | --- | --- | --- | --- | --- | --- | --- | --- | --- | --- | --- | --- | --- | --- | --- | --- | --- | --- | --- | --- | --- | --- | --- | --- | --- | --- | --- | --- | --- | --- | --- | --- | --- | --- | --- | --- | --- | --- | --- | --- | --- | --- | --- | --- | --- | --- | --- | --- | --- | --- | --- | --- | --- | --- | --- | --- | --- | --- | --- | --- | --- | --- | --- | --- | --- | --- | --- | --- | --- | --- | --- | --- | --- | --- | --- | --- | --- | --- | --- |

^1^Original starting values were subtracted from final values after exposure.

^2^Optical density at 600 nm

(± SD) Standard deviation

(*) Significant (+) effect of pH on cell dispersal

Table S3. Statistical comparison of *V. parahaemolyticus* and *V. vulnificus* overall biofilm biomass^1^ and cell dispersal^2^ values in nutrient rich media compared to nutrient starved.

|  |  | **Coefficient** | ***p*** |
| --- | --- | --- | --- |
| *V. parahaemolyticus* | **Biofilm Biomass** | 0.13 | 1.00 |
|  | **Cell Dispersal** | 0.04 | 1.00 |
| *V. vulnificus* | **Biofilm Biomass** | -0.03 | 1.00 |
|  | **Cell Dispersal** | 0.029 | 1.00 |

^1^Optical density at 570 nm

^2^Optical density at 600 nm

Table S4. Statistical comparison of *V. parahaemolyticus* and *V. vulnificus* biofilm biomass^1^ and cell dispersal^2^ values at 37°C compared to 25°C.

|  |  | **Coefficient** | ***p*** |
| --- | --- | --- | --- |
| *V. parahaemolyticus* | **Biofilm Biomass** | -0.01 | 1.00 |
|  | **Cell Dispersal** | 0.01 | 1.00 |
| *V. vulnificus* | **Biofilm Biomass** | -0.02 | 1.00 |
|  | **Cell Dispersal** | -0.02 | 1.00 |

^1^Optical density at 570 nm

^2^Optical density at 600 nm

Table S5. Statistical comparison of effect of pH on *V. parahaemolyticus* and *V. vulnificus* overall biofilm biomass^1^ and cell dispersal^2^.

|  |  | **Coefficient** | ***p*** |
| --- | --- | --- | --- |
| *V. parahaemolyticus* | **Biofilm Biomass** | 0.14* | <0.05 |
|  | **Cell Dispersal** | 0.02* | <0.001 |
| *V. vulnificus* | **Biofilm Biomass** | 0.02 | 0.78 |
|  | **Cell Dispersal** | 0.01 | 0.06 |

^1^Optical density at 570 nm

^2^Optical density at 600 nm

*Significant increase (+) in biofilm biomass or cell dispersal

Table S6. Statistical comparison of pH on *V. parahaemolyticus* and *V. vulnificus* overall biofilm biomass^1^ and cell dispersal^2^ values in nutrient rich media compared to nutrient starved.

|  |  | **Coefficient** | ***p*** |
| --- | --- | --- | --- |
| *V. parahaemolyticus* | **Biofilm Biomass** | -0.07 | 1.00 |
|  | **Cell Dispersal** | -0.01 | 0.051 |
| *V. vulnificus* | **Biofilm Biomass** | -0.01 | 1.00 |
|  | **Cell Dispersal** | -0.01 | 1.00 |

^1^Optical density at 570 nm

^2^Optical density at 600 nm

Table S7. Statistical comparison of pH on *V. parahaemolyticus* and *V. vulnificus* overall biofilm biomass^1^ and cell dispersal^2^ values at 37°C compared to 25°C.

|  |  | **Coefficient** | ***p*** |
| --- | --- | --- | --- |
| *V. parahaemolyticus* | **Biofilm Biomass** | 0.01 | 1.00 |
|  | **Cell Dispersal** | 0.001 | 1.00 |
| *V. vulnificus* | **Biofilm Biomass** | 0.003 | 1.00 |
|  | **Cell Dispersal** | 0.01 | 1.00 |

^1^Optical density at 570 nm

^2^Optical density at 600 nm

Table S8. Statistical comparison of *V. parahaemolyticus* and *V. vulnificus* biofilm biomass^1^ and cell dispersal^2^ values at 37°C and nutrient starved media compared to 25°C and nutrient rich media.

|  |  | **Coefficient** | ***p*** |
| --- | --- | --- | --- |
| *V. parahaemolyticus* | **Biofilm Biomass** | 0.23 | 1.00 |
|  | **Cell Dispersal** | 0.003 | 1.00 |
| *V. vulnificus* | **Biofilm Biomass** | -0.01 | 1.00 |
|  | **Cell Dispersal** | 0.03 | 1.00 |

^1^Optical density at 570 nm

^2^Optical density at 600 nm

Table S9. Statistical comparison of pH on *V. parahaemolyticus* and *V. vulnificus* overall biofilm biomass and cell dispersal values at 37°C and nutrient starved media compared to 25°C and nutrient rich media.

|  |  | **Coefficient** | ***p*** |
| --- | --- | --- | --- |
| *V. parahaemolyticus* | **Biofilm Biomass** | 0.001 | 1.00 |
|  | **Cell Dispersal** | -0.004 | 1.00 |
| *V. vulnificus* | **Biofilm Biomass** | -0.002 | 1.00 |
|  | **Cell Dispersal** | -0.01 | 1.00 |

^1^Optical density at 570 nm

^2^Optical density at 600 nm

Table S10. Effect of pH on *V. parahaemolyticus* and *V. vulnificus* overall biofilm metabolic activity^1^ in nutrient rich media.

| **Species** | **Time (mins)** | **pH 3** | **pH 4** | **pH 5** | **pH 6** | **pH 7** | **pH 7.4** | **pH 8.1** |
| --- | --- | --- | --- | --- | --- | --- | --- | --- |
| *V. parahaemolyticus* | 0 | 465 | 133352 | 97380 | 92458 | 148801 | 129109 | 92084 |
|  | 15 | 286 | 934148 | 790664 | 679942 | 681085 | 640597 | 302071 |
|  | 30 | 513 | 1625459 | 773090 | 652453 | 581516 | 567817 | 393652 |
|  | 45 | 683 | 1797693 | 528793 | 476061 | 382226 | 415707 | 380048 |
|  | 60 | 1160 | 1758139 | 366432 | 372790 | 288373 | 318948 | 385735 |
|  | 75 | 1102 | 1532370 | 318165 | 333364 | 239621 | 267150 | 320372 |
|  | 90 | 1486 | 1314069 | 282378 | 298002 | 218032 | 233587 | 270520 |
|  | 105 | 1697 | 1094049 | 248619 | 270474 | 210938 | 212931 | 224025 |
|  | 120 | 1757 | 916987 | 222512 | 244677 | 198093 | 193906 | 190045 |
|  | 135 | 1778 | 731128 | 201113 | 222818 | 183718 | 178245 | 164855 |
|  | 150 | 1937 | 633560 | 185672 | 204646 | 170719 | 164692 | 147410 |
|  | 165 | 1909 | 514445 | 175203 | 192433 | 160585 | 153515 | 132661 |
|  | 180 | 2203 | 426426 | 165771 | 181720 | 149078 | 144249 | 120382 |
|  | 195 | 2789 | 358051 | 158575 | 172595 | 140166 | 136787 | 111248 |
|  | 210 | 3735 | 295963 | 151483 | 164874 | 132528 | 131289 | 103847 |
|  | 225 | 3664 | 255637 | 144267 | 156076 | 125214 | 124564 | 97966 |
|  | 240 | 4814 | 227748 | 138078 | 148832 | 118265 | 118960 | 93398 |
| *V. vulnificus* | 0 | 4039 | 525594 | 336034 | 195092 | 318675 | 293732 | 851461 |
|  | 15 | 1193 | 3561368 | 1818971 | 1151454 | 1441191 | 1821863 | 944692 |
|  | 30 | 4071 | 6194012 | 1996966 | 1593665 | 1714198 | 1987753 | 1175000 |
|  | 45 | 8179 | 8175410 | 1525409 | 1141948 | 1356433 | 1515855 | 915481 |
|  | 60 | 5884 | 10061465 | 1016654 | 742752 | 860864 | 1046205 | 628052 |
|  | 75 | 1498 | 9865254 | 687005 | 510502 | 594934 | 725676 | 430376 |
|  | 90 | 2665 | 11538569 | 537804 | 397441 | 466838 | 553671 | 351663 |
|  | 105 | 2679 | 10780104 | 440741 | 346739 | 388345 | 470112 | 311756 |
|  | 120 | 1798 | 12739924 | 372688 | 300674 | 335688 | 399467 | 271323 |
|  | 135 | 2272 | 12045019 | 307411 | 257531 | 292776 | 327634 | 230356 |
|  | 150 | 2084 | 13629684 | 256959 | 219224 | 249749 | 275453 | 201278 |
|  | 165 | 1 | 14269065 | 223923 | 190595 | 224930 | 240716 | 179840 |
|  | 180 | 864 | 13645904 | 194065 | 165031 | 204985 | 214506 | 165493 |
|  | 195 | 1 | 13131283 | 173206 | 145816 | 187554 | 194298 | 151565 |
|  | 210 | 1 | 11008931 | 157851 | 133663 | 174563 | 178435 | 141835 |
|  | 225 | 737 | 8998519 | 145951 | 125470 | 164181 | 165515 | 134865 |
|  | 240 | 374 | 6402889 | 134861 | 117178 | 153837 | 154681 | 128917 |
|  |  |  |  |  |  |  |  |  |

^1^RFU values were divided by corresponding optical density at 600 nm values for normalization.

Table S11. Effect of pH on *V. parahaemolyticus* and *V. vulnificus* overall biofilm metabolic activity^1^ in nutrient starved media.

| **Species** | **Time (mins)** | | **pH 3** | **pH 4** | **pH 5** | **pH 6** | **pH 7** | **pH 7.4** | **pH 8.1** |
| --- | --- | --- | --- | --- | --- | --- | --- | --- | --- |
| *V. parahaemolyticus* | 0 | 111042 | | 72249 | 71617 | 82995 | 106526 | 85659 | 34317 |
|  | 15 | 1781 | | 259117 | 254908 | 280957 | 352627 | 309581 | 286182 |
|  | 30 | 4694 | | 373421 | 350576 | 385467 | 480384 | 434813 | 390175 |
|  | 45 | 8779 | | 369668 | 337151 | 366928 | 449412 | 439934 | 385149 |
|  | 60 | 12974 | | 345855 | 349603 | 392238 | 398267 | 403008 | 371770 |
|  | 75 | 17936 | | 304757 | 320943 | 352108 | 334645 | 335753 | 345270 |
|  | 90 | 24938 | | 272767 | 295077 | 332519 | 293595 | 294101 | 328365 |
|  | 105 | 32711 | | 244896 | 265320 | 303065 | 252472 | 252592 | 297475 |
|  | 120 | 44415 | | 211799 | 237287 | 267792 | 215067 | 212959 | 261436 |
|  | 135 | 61652 | | 183651 | 207952 | 228523 | 187186 | 183341 | 225476 |
|  | 150 | 79779 | | 163006 | 181258 | 195992 | 165219 | 161065 | 198448 |
|  | 165 | 108830 | | 144724 | 158382 | 172457 | 145163 | 141955 | 174033 |
|  | 180 | 151690 | | 130402 | 140683 | 151215 | 128924 | 126542 | 153125 |
|  | 195 | 213151 | | 118726 | 127421 | 135710 | 115922 | 112751 | 140541 |
|  | 210 | 259983 | | 108407 | 116913 | 122839 | 103100 | 101359 | 131375 |
|  | 225 | 274535 | | 99866 | 108102 | 115085 | 94890 | 94120 | 122166 |
|  | 240 | 270501 | | 94115 | 101915 | 108195 | 88477 | 88119 | 113569 |
| *V. vulnificus* | 0 | 1 | | 213789 | 531168 | 307942 | 1347714 | 1055601 | 262153 |
|  | 15 | 1 | | 487114 | 856258 | 630045 | 1991611 | 2003535 | 2007300 |
|  | 30 | 1 | | 576798 | 941143 | 792108 | 1794634 | 1696657 | 1829650 |
|  | 45 | 1 | | 594632 | 818451 | 706894 | 1346586 | 1339767 | 1405968 |
|  | 60 | 1 | | 522266 | 615717 | 460869 | 945798 | 913211 | 952219 |
|  | 75 | 1 | | 410758 | 434206 | 342002 | 612217 | 601857 | 604524 |
|  | 90 | 1 | | 344479 | 342958 | 278666 | 435925 | 457582 | 457580 |
|  | 105 | 1 | | 290315 | 285935 | 249795 | 330647 | 365094 | 364821 |
|  | 120 | 1 | | 259447 | 258910 | 235744 | 283586 | 317220 | 317516 |
|  | 135 | 1 | | 230908 | 229131 | 216499 | 247836 | 272544 | 276344 |
|  | 150 | 1 | | 204545 | 202038 | 193509 | 218007 | 234392 | 237808 |
|  | 165 | 1 | | 177602 | 176419 | 164553 | 189141 | 199555 | 203905 |
|  | 180 | 1 | | 156711 | 157408 | 148408 | 169433 | 174699 | 177719 |
|  | 195 | 1 | | 141464 | 141739 | 133096 | 154635 | 156296 | 159311 |
|  | 210 | 1 | | 129729 | 132140 | 122313 | 141701 | 143491 | 145510 |
|  | 225 | 1 | | 119836 | 121967 | 113286 | 130174 | 130883 | 133245 |
|  | 240 | 1 | | 113545 | 115870 | 107821 | 121816 | 121755 | 124107 |
|  | 0 | 1 | | 213789 | 531168 | 307942 | 1347714 | 1055601 | 262153 |

^1^RFU values were divided by corresponding optical density at 600 nm values for normalization.

# Table S12. Effect of pH, temperature, and nutrient availability on mean biofilm biomass^1^ on LDPE^2^ by *V. parahaemolyticus* and *V. vulnificus*.

| \| **Species** \| **pH** \| **Nutrient Rich (25°C)**  **(± SD)** \| **Nutrient Rich (37°C)**  **(± SD)** \| **Nutrient Starved (25°C) (± SD)** \| **Nutrient Starved (37°C) (± SD)** \| \| --- \| --- \| --- \| --- \| --- \| --- \| \| *V. parahaemolyticus** \| 3 \| 0.0046  0.0050 \| 0.0059  0.0028 \| 0.0037  0.0008 \| 0.0045  0.0029 \| \|  \| 4 \| 0.0065  0.0051 \| 0.0060  0.0051 \| 0.0040  0.0031 \| 0.0022  0.0022 \| \|  \| 5 \| 0.0139  0.0075 \| 0.0177  0.0036 \| 0.0047  0.0023 \| 0.0048  0.0042 \| \|  \| 6 \| 0.0187  0.0056 \| 0.0165  0.0070 \| 0.0057  0.0030 \| 0.0044  0.0037 \| \|  \| 7 \| 0.0129  0.0060 \| 0.0152  0.0034 \| 0.0062  0.0027 \| 0.0026  0.0020 \| \|  \| 7.4 \| 0.0136  0.0070 \| 0.0134  0.0030 \| 0.0035  0.0033 \| 0.0043  0.0038 \| \|  \| 8.1 \| 0.0284  0.0068 \| 0.0127  0.0018 \| 0.0056  0.0025 \| 0.0046  0.0046 \| \| *V. vulnificus* \| 3 \| 0.0003  0.0003 \| 0.0005  0.0003 \| 0.0012  0.0010 \| 0.0011  0.0006 \| \|  \| 4 \| 0.0004  0.0004 \| 0.0003  0.0000 \| 0.0005  0.0004 \| 0.0007  0.0001 \| \|  \| 5 \| 0.0017  0.0014 \| 0.0018  0.0016 \| 0.0006  0.0002 \| 0.0006  0.0001 \| \|  \| 6 \| 0.0012  0.0005 \| 0.0028  0.0019 \| 0.0004  0.0002 \| 0.0005  0.0001 \| \|  \| 7 \| 0.0024  0.0023 \| 0.0032  0.0041 \| 0.0007  0.0005 \| 0.0008  0.0003 \| \|  \| 7.4 \| 0.0013  0.0010 \| 0.0013  0.0008 \| 0.0005  0.0002 \| 0.0006  0.0005 \| \|  \| 8.1 \| 0.0014  0.0006 \| 0.0003  0.0003 \| 0.0013  0.0009 \| 0.0005  0.0001 \| |
| --- | --- | --- | --- | --- | --- | --- | --- | --- | --- | --- | --- | --- | --- | --- | --- | --- | --- | --- | --- | --- | --- | --- | --- | --- | --- | --- | --- | --- | --- | --- | --- | --- | --- | --- | --- | --- | --- | --- | --- | --- | --- | --- | --- | --- | --- | --- | --- | --- | --- | --- | --- | --- | --- | --- | --- | --- | --- | --- | --- | --- | --- | --- | --- | --- | --- | --- | --- | --- | --- | --- | --- | --- | --- | --- | --- | --- | --- | --- | --- | --- | --- | --- | --- | --- | --- | --- | --- | --- | --- | --- |

^1^Optical density at 570 nm

^2^Biomass values divided by total microplastic surface area (24 mm^2^)

(± SD) Standard deviation

(*) Significant (+) effect of pH on biofilm biomass

Table S13. Effect of pH, temperature, and nutrient availability on *V. parahaemolyticus* and *V. vulnificus* CFUs^1^ on LDPE^2^.

| \| **Species** \| **pH** \| **Nutrient Rich (25°C)**  **(± SD)** \| **Nutrient Rich (37°C)**  **(± SD)** \| **Nutrient Starved (25°C) (± SD)** \| **Nutrient Starved (37°C) (± SD)** \| \| --- \| --- \| --- \| --- \| --- \| --- \| \| *V. parahaemolyticus* \| 3 \| 0.0000  0.0000 \| 0.8148  0.5879 \| 1.5659  0.4331 \| 0.0000  0.0000 \| \|  \| 4 \| 4.6451  3.3655 \| 2.3882  1.0206 \| 5.3602  4.7049 \| 2.8985  1.8583 \| \|  \| 5 \| 5.1112  3.8583 \| 5.1555  4.5776 \| 4.6519  3.7792 \| 3.8985  3.2218 \| \|  \| 6 \| 5.0969  4.1594 \| 4.8416  4.2975 \| 5.7315  5.2358 \| 4.8148  4.3999 \| \|  \| 7 \| 5.5708  4.7962 \| 4.2145  3.7442 \| 5.4731  4.4952 \| 4.9061  3.8038 \| \|  \| 7.4 \| 5.0292  4.5027 \| 5.3495  4.3746 \| 5.5283  4.9526 \| 4.1204  3.2843 \| \|  \| 8.1 \| 4.3522  3.3434 \| 4.6805  4.0644 \| 5.4327  4.4649 \| 4.7861  4.1048 \| \| *V. vulnificus* \| 3 \| 0.0000  0.0000 \| 0.0000  0.0000 \| 0.0000  0.0000 \| 0.0000  0.0000 \| \|  \| 4 \| 2.6703  1.1980 \| 0.0000  0.0000 \| 2.3216  1.5240 \| 3.3782  2.2843 \| \|  \| 5 \| 3.2599  2.8284 \| 3.6051  3.0005 \| 3.7669  2.7223 \| 3.9280  3.5977 \| \|  \| 6 \| 3.7382  3.6091 \| 3.5960  3.3029 \| 3.8485  2.9796 \| 3.5945  2.6964 \| \|  \| 7 \| 3.3413  2.7303 \| 2.1158  1.6618 \| 3.5576  2.7833 \| 3.8111  3.2974 \| \|  \| 7.4 \| 3.3955  3.2345 \| 2.9251  2.0636 \| 3.8587  3.2943 \| 3.7523  2.9427 \| \|  \| 8.1 \| 2.4987  2.0178 \| 2.1017  1.4432 \| 4.2465  3.1269 \| 3.7167  2.5819 \| \|  \|  \|  \|  \|  \|  \| |
| --- | --- | --- | --- | --- | --- | --- | --- | --- | --- | --- | --- | --- | --- | --- | --- | --- | --- | --- | --- | --- | --- | --- | --- | --- | --- | --- | --- | --- | --- | --- | --- | --- | --- | --- | --- | --- | --- | --- | --- | --- | --- | --- | --- | --- | --- | --- | --- | --- | --- | --- | --- | --- | --- | --- | --- | --- | --- | --- | --- | --- | --- | --- | --- | --- | --- | --- | --- | --- | --- | --- | --- | --- | --- | --- | --- | --- | --- | --- | --- | --- | --- | --- | --- | --- | --- | --- | --- | --- | --- | --- | --- | --- | --- | --- | --- | --- |

^1^CFU values were log transformed

^2^CFU values divided by total coupon surface area (24 mm^2^)

(± SD) Standard deviation

# Table S14. Effect of pH on mean cell colonization^1^ on microplates from LDPE by *V. parahaemolyticus* and *V. vulnificus*.

| \| **Species** \| **pH** \| **Nutrient Rich (25°C)**  **(± SD)** \| **Nutrient Rich (37°C)**  **(± SD)** \| **Nutrient Starved (25°C) (± SD)** \| **Nutrient Starved (37°C) (± SD)** \| \| --- \| --- \| --- \| --- \| --- \| --- \| \| *V. parahaemolyticus* \| 3 \| 0.137  0.203 \| 0.132  0.172 \| 0.030  0.021 \| 0.017  0.008 \| \|  \| 4 \| 0.143  0.178 \| 0.105  0.130 \| 0.020  0.008 \| 0.009  0.005 \| \|  \| 5 \| 0.175  0.217 \| 0.345  0.288 \| 0.020  0.009 \| 0.016  0.014 \| \|  \| 6 \| 0.258  0.282 \| 0.343  0.241 \| 0.028  0.007 \| 0.012  0.007 \| \|  \| 7 \| 0.251  0.209 \| 0.325  0.098 \| 0.018  0.007 \| 0.009  0.007 \| \|  \| 7.4 \| 0.138  0.035 \| 0.343  0.248 \| 0.016  0.011 \| 0.013  0.009 \| \|  \| 8.1 \| 0.129  0.014 \| 0.320  0.031 \| 0.018  0.009 \| 0.014  0.016 \| \| *V. vulnificus** \| 3 \| 0.009  0.009 \| 0.007  0.003 \| 0.005  0.005 \| 0.011  0.003 \| \|  \| 4 \| 0.010  0.007 \| 0.008  0.003 \| 0.003  0.004 \| 0.012  0.006 \| \|  \| 5 \| 0.042  0.015 \| 0.032  0.017 \| 0.007  0.009 \| 0.009  0.002 \| \|  \| 6 \| 0.040  0.026 \| 0.074  0.053 \| 0.018  0.003 \| 0.016  0.004 \| \|  \| 7 \| 0.064  0.042 \| 0.047  0.020 \| 0.005  0.004 \| 0.008  0.001 \| \|  \| 7.4 \| 0.029  0.011 \| 0.043  0.028 \| 0.003  0.003 \| 0.010  0.006 \| \|  \| 8.1 \| 0.056  0.009 \| 0.046  0.023 \| 0.008  0.003 \| 0.007  0.005 \| |
| --- | --- | --- | --- | --- | --- | --- | --- | --- | --- | --- | --- | --- | --- | --- | --- | --- | --- | --- | --- | --- | --- | --- | --- | --- | --- | --- | --- | --- | --- | --- | --- | --- | --- | --- | --- | --- | --- | --- | --- | --- | --- | --- | --- | --- | --- | --- | --- | --- | --- | --- | --- | --- | --- | --- | --- | --- | --- | --- | --- | --- | --- | --- | --- | --- | --- | --- | --- | --- | --- | --- | --- | --- | --- | --- | --- | --- | --- | --- | --- | --- | --- | --- | --- | --- | --- | --- | --- | --- | --- | --- |

^1^Optical density at 570 nm

(± SD) Standard deviation

(*) Significant (+) effect of pH on biofilm biomass

Table S15. Effect of pH, temperature and nutrient availability on *V. parahaemolyticus* and *V. vulnificus* CFUs^1^ dispersed from LDPE.

| \| **Species** \| **pH** \| **Nutrient Rich (25°C)**  **(± SD)** \| **Nutrient Rich (37°C)**  **(± SD)** \| **Nutrient Starved (25°C) (± SD)** \| **Nutrient Starved (37°C) (± SD)** \| \| --- \| --- \| --- \| --- \| --- \| --- \| \| *V. parahaemolyticus* \| 3 \| 0.0000  0.0000 \| 0.0000  0.0000 \| 0.0000  0.0000 \| 0.0000  0.0000 \| \|  \| 4 \| 7.2050  6.1868 \| 2.1249  2.1840 \| 7.5141  6.8234 \| 6.0374  5.2436 \| \|  \| 5 \| 7.5185  6.2386 \| 8.1173  7.1963 \| 6.9138  5.8997 \| 7.6057  6.6065 \| \|  \| 6 \| 7.7297  7.0402 \| 7.6659  6.5071 \| 7.2041  6.2386 \| 7.5760  6.8466 \| \|  \| 7 \| 7.7533  7.0113 \| 7.1996  6.5129 \| 7.4150  7.0187 \| 7.5051  6.6611 \| \|  \| 7.4 \| 7.8084  7.1868 \| 7.5315  6.9934 \| 7.4771  6.7782 \| 7.4914  6.6394 \| \|  \| 8.1 \| 6.9294  6.3356 \| 7.6368  6.9755 \| 7.4094  6.5782 \| 7.5873  6.8675 \| \| *V. vulnificus* \| 3 \| 0.0000  0.0000 \| 0.0000  0.0000 \| 0.0000  0.0000 \| 0.0000  0.0000 \| \|  \| 4 \| 4.3243  3.9952 \| 0.0000  0.0000 \| 4.1856  3.4008 \| 5.0427  4.4519 \| \|  \| 5 \| 5.5873  4.7614 \| 7.1996  7.0451 \| 5.8865  5.0467 \| 5.9085  5.5024 \| \|  \| 6 \| 5.0731  4.9454 \| 7.4430  6.9397 \| 5.5097  4.7548 \| 5.4771  4.7955 \| \|  \| 7 \| 4.9638  4.8443 \| 6.6690  6.3118 \| 5.3358  4.6541 \| 5.3802  5.0246 \| \|  \| 7.4 \| 5.5873  4.5071 \| 6.7243  6.2944 \| 5.7132  4.9578 \| 4.9853  4.4008 \| \|  \| 8.1 \| 5.6812  5.1139 \| 6.8573  6.7300 \| 5.6092  4.8466 \| 5.3358  4.9755 \| \|  \|  \|  \|  \|  \|  \| |
| --- | --- | --- | --- | --- | --- | --- | --- | --- | --- | --- | --- | --- | --- | --- | --- | --- | --- | --- | --- | --- | --- | --- | --- | --- | --- | --- | --- | --- | --- | --- | --- | --- | --- | --- | --- | --- | --- | --- | --- | --- | --- | --- | --- | --- | --- | --- | --- | --- | --- | --- | --- | --- | --- | --- | --- | --- | --- | --- | --- | --- | --- | --- | --- | --- | --- | --- | --- | --- | --- | --- | --- | --- | --- | --- | --- | --- | --- | --- | --- | --- | --- | --- | --- | --- | --- | --- | --- | --- | --- | --- | --- | --- | --- | --- | --- | --- |

^1^CFU values were log transformed

(± SD) Standard deviation

# Table S16. Effect of pH on mean biofilm biomass^1^ on PP^2^ by *V. parahaemolyticus* and *V. vulnificus*.

| \| **Species** \| **pH** \| **Nutrient Rich (25°C)**  **(± SD)** \| **Nutrient Rich (37°C)**  **(± SD)** \| **Nutrient Starved (25°C) (± SD)** \| **Nutrient Starved (37°C) (± SD)** \| \| --- \| --- \| --- \| --- \| --- \| --- \| \| *V. parahaemolyticus* \| 3 \| 0.0039  0.0015 \| 0.0070  0.0078 \| 0.0099  0.0049 \| 0.0095  0.0052 \| \|  \| 4 \| 0.0067  0.0052 \| 0.0042  0.0041 \| 0.0085  0.0021 \| 0.0098  0.0063 \| \|  \| 5 \| 0.0207  0.0129 \| 0.0040  0.0032 \| 0.0084  0.0017 \| 0.0036  0.0044 \| \|  \| 6 \| 0.0149  0.0068 \| 0.0087  0.0096 \| 0.0090  0.0033 \| 0.0030  0.0029 \| \|  \| 7 \| 0.0126  0.0088 \| 0.0070  0.0041 \| 0.0131  0.0077 \| 0.0087  0.0046 \| \|  \| 7.4 \| 0.0089  0.0021 \| 0.0051  0.0047 \| 0.0086  0.0027 \| 0.0098  0.0040 \| \|  \| 8.1 \| 0.0151  0.0032 \| 0.0106  0.0013 \| 0.0093  0.0062 \| 0.0092  0.0001 \| \| *V. vulnificus* \| 3 \| 0.0003  0.0004 \| 0.0002  0.0002 \| 0.0002  0.0002 \| 0.0001  0.0001 \| \|  \| 4 \| 0.0004  0.0003 \| 0.0006  0.0004 \| 0.0001  0.0000 \| 0.0002  0.0001 \| \|  \| 5 \| 0.0011  0.0012 \| 0.0008  0.0000 \| 0.0001  0.0001 \| 0.0001  0.0001 \| \|  \| 6 \| 0.0007  0.0002 \| 0.0017  0.0005 \| 0.0001  0.0000 \| 0.0002  0.0001 \| \|  \| 7 \| 0.0007  0.0007 \| 0.0015  0.0010 \| 0.0001  0.0001 \| 0.0002  0.0001 \| \|  \| 7.4 \| 0.0003  0.0001 \| 0.0011  0.0007 \| 0.0001  0.0001 \| 0.0000  0.0001 \| \|  \| 8.1 \| 0.0008  0.0005 \| 0.0004  0.0001 \| 0.0001  0.0001 \| 0.0001  0.0001 \| |
| --- | --- | --- | --- | --- | --- | --- | --- | --- | --- | --- | --- | --- | --- | --- | --- | --- | --- | --- | --- | --- | --- | --- | --- | --- | --- | --- | --- | --- | --- | --- | --- | --- | --- | --- | --- | --- | --- | --- | --- | --- | --- | --- | --- | --- | --- | --- | --- | --- | --- | --- | --- | --- | --- | --- | --- | --- | --- | --- | --- | --- | --- | --- | --- | --- | --- | --- | --- | --- | --- | --- | --- | --- | --- | --- | --- | --- | --- | --- | --- | --- | --- | --- | --- | --- | --- | --- | --- | --- | --- | --- |

^1^Optical density at 570 nm

^2^Biomass values divided by total microplastic surface area (24 mm^2^)

(± SD) Standard deviation

Table S17. Effect of pH, temperature, and nutrient availability on *V. parahaemolyticus* and *V. vulnificus* CFUs^1^ on PP^2^.

| \| **Species** \| **pH** \| **Nutrient Rich (25°C)**  **(± SD)** \| **Nutrient Rich (37°C)**  **(± SD)** \| **Nutrient Starved (25°C) (± SD)** \| **Nutrient Starved (37°C) (± SD)** \| \| --- \| --- \| --- \| --- \| --- \| --- \| \| *V. parahaemolyticus* \| 3 \| 1.8157  0.5740 \| 0.0000  0.0000 \| 0.0000  0.0000 \| 0.0000  0.0000 \| \|  \| 4 \| 5.2326  4.2218 \| 1.7901  0.8810 \| 5.1761  4.4039 \| 4.7002  3.7728 \| \|  \| 5 \| 4.8248  3.9694 \| 4.3882  3.5749 \| 5.3522  4.0423 \| 5.2145  4.4952 \| \|  \| 6 \| 5.1680  4.1653 \| 4.0235  3.7833 \| 5.4237  4.3999 \| 5.4145  4.6411 \| \|  \| 7 \| 4.4480  3.5879 \| 5.6255  4.5879 \| 5.3010  4.2218 \| 5.4003  4.5495 \| \|  \| 7.4 \| 4.8822  3.8985 \| 4.6212  3.8413 \| 5.2290  4.5776 \| 5.1996  3.6198 \| \|  \| 8.1 \| 5.2255  4.5101 \| 4.2396  3.4432 \| 5.2533  4.0969 \| 4.6741  3.8102 \| \| *V. vulnificus* \| 3 \| 0.0000  0.0000 \| 0.0000  0.0000 \| 0.0000  0.0000 \| 0.0000  0.0000 \| \|  \| 4 \| 0.0000  0.0000 \| 0.0000  0.0000 \| 3.1065  2.3812 \| 3.9351  2.8657 \| \|  \| 5 \| 2.9280  2.4432 \| 3.0474  2.3878 \| 3.2919  2.6262 \| 3.6612  2.0969 \| \|  \| 6 \| 2.6638  2.0936 \| 2.1513  2.0286 \| 4.0000  3.0423 \| 3.8909  2.6823 \| \|  \| 7 \| 2.1427  1.3216 \| 1.5406  0.3812 \| 3.4751  2.8259 \| 3.9406  2.5101 \| \|  \| 7.4 \| 2.7638  2.3620 \| 3.0921  2.3607 \| 3.9001  3.1064 \| 3.7179  3.1523 \| \|  \| 8.1 \| 2.1721  1.7478 \| 1.9351  1.4990 \| 3.8073  2.8591 \| 3.7304  3.1438 \| \|  \|  \|  \|  \|  \|  \|   ^1^CFU values were log transformed  ^2^CFU values divided by total coupon surface area (24 mm^2^)  (± SD) Standard deviation |
| --- | --- | --- | --- | --- | --- | --- | --- | --- | --- | --- | --- | --- | --- | --- | --- | --- | --- | --- | --- | --- | --- | --- | --- | --- | --- | --- | --- | --- | --- | --- | --- | --- | --- | --- | --- | --- | --- | --- | --- | --- | --- | --- | --- | --- | --- | --- | --- | --- | --- | --- | --- | --- | --- | --- | --- | --- | --- | --- | --- | --- | --- | --- | --- | --- | --- | --- | --- | --- | --- | --- | --- | --- | --- | --- | --- | --- | --- | --- | --- | --- | --- | --- | --- | --- | --- | --- | --- | --- | --- | --- | --- | --- | --- | --- | --- | --- |

# Table S18. Effect of pH on mean cell colonization^1^ on microplates from PP by *V. parahaemolyticus* and *V. vulnificus*.

| \| **Species** \| **pH** \| **Nutrient Rich (25°C)**  **(± SD)** \| **Nutrient Rich (37°C)**  **(± SD)** \| **Nutrient Starved (25°C) (± SD)** \| **Nutrient Starved (37°C) (± SD)** \| \| --- \| --- \| --- \| --- \| --- \| --- \| \| *V. parahaemolyticus*# \| 3 \| 0.021  0.011 \| 0.044  0.029 \| 0.012  0.006 \| 0.015  0.007 \| \|  \| 4 \| 0.024  0.011 \| 0.029  0.009 \| 0.023  0.010 \| 0.030  0.006 \| \|  \| 5 \| 0.074  0.023 \| 0.115  0.031 \| 0.023  0.008 \| 0.016  0.016 \| \|  \| 6 \| 0.059  0.013 \| 0.218  0.107 \| 0.021  0.007 \| 0.018  0.015 \| \|  \| 7 \| 0.104  0.017 \| 0.287  0.077 \| 0.028  0.007 \| 0.016  0.008 \| \|  \| 7.4 \| 0.089  0.004 \| 0.232  0.057 \| 0.029  0.004 \| 0.029  0.010 \| \|  \| 8.1 \| 0.111  0.045 \| 0.263  0.026 \| 0.026  0.006 \| 0.029  0.008 \| \| *V. vulnificus** \| 3 \| 0.004  0.004 \| 0.011  0.011 \| 0.006  0.005 \| 0.004  0.001 \| \|  \| 4 \| 0.007  0.006 \| 0.005  0.003 \| 0.005  0.005 \| 0.006  0.006 \| \|  \| 5 \| 0.016  0.008 \| 0.020  0.022 \| 0.007  0.005 \| 0.007  0.003 \| \|  \| 6 \| 0.020  0.010 \| 0.073  0.026 \| 0.004  0.003 \| 0.009  0.005 \| \|  \| 7 \| 0.032  0.014 \| 0.071  0.038 \| 0.005  0.003 \| 0.008  0.003 \| \|  \| 7.4 \| 0.028  0.003 \| 0.049  0.038 \| 0.005  0.004 \| 0.005  0.004 \| \|  \| 8.1 \| 0.052  0.016 \| 0.037  0.008 \| 0.010  0.009 \| 0.011  0.001 \| |
| --- | --- | --- | --- | --- | --- | --- | --- | --- | --- | --- | --- | --- | --- | --- | --- | --- | --- | --- | --- | --- | --- | --- | --- | --- | --- | --- | --- | --- | --- | --- | --- | --- | --- | --- | --- | --- | --- | --- | --- | --- | --- | --- | --- | --- | --- | --- | --- | --- | --- | --- | --- | --- | --- | --- | --- | --- | --- | --- | --- | --- | --- | --- | --- | --- | --- | --- | --- | --- | --- | --- | --- | --- | --- | --- | --- | --- | --- | --- | --- | --- | --- | --- | --- | --- | --- | --- | --- | --- | --- | --- |

^1^Optical density at 570 nm

(± SD) Standard deviation

(*) Significant effect of pH on biofilm biomass

(#) Synergistic significant effect of pH, temperature, and nutrient content on biofilm biomass

Table S19. Effect of pH, temperature, and nutrient availability on *V. parahaemolyticus* and *V. vulnificus* CFUs^1^ dispersed from PP.

| \| **Species** \| **pH** \| **Nutrient Rich (25°C)**  **(± SD)** \| **Nutrient Rich (37°C)**  **(± SD)** \| **Nutrient Starved (25°C) (± SD)** \| **Nutrient Starved (37°C) (± SD)** \| \| --- \| --- \| --- \| --- \| --- \| --- \| \| *V. parahaemolyticus* \| 3 \| 0.0000  0.000 \| 0.0000  0.000 \| 0.0000  0.000 \| 0.0000  0.000 \| \|  \| 4 \| 7.3754  6.243 \| 3.3153  2.859 \| 7.3617  6.893 \| 7.5911  7.000 \| \|  \| 5 \| 7.5399  6.318 \| 7.7782  7.551 \| 7.7508  6.702 \| 7.5948  6.983 \| \|  \| 6 \| 7.5523  6.710 \| 7.6812  7.268 \| 7.7104  7.185 \| 7.7104  6.665 \| \|  \| 7 \| 7.9226  7.663 \| 7.8409  7.486 \| 7.4037  6.755 \| 7.6690  7.001 \| \|  \| 7.4 \| 7.6402  6.546 \| 7.9412  7.040 \| 7.3010  6.778 \| 7.5006  6.318 \| \|  \| 8.1 \| 7.8751  7.149 \| 7.9777  7.221 \| 7.4150  6.845 \| 7.3741  6.184 \| \| *V. vulnificus* \| 3 \| 0.0000  0.000 \| 0.0000  0.000 \| 0.0000  0.000 \| 0.0000  0.000 \| \|  \| 4 \| 0.0000  0.000 \| 0.0000  0.000 \| 2.8239  2.485 \| 5.3188  4.703 \| \|  \| 5 \| 6.4276  5.838 \| 5.6368  5.001 \| 5.7993  5.516 \| 5.6990  5.114 \| \|  \| 6 \| 5.1249  4.741 \| 6.2529  5.980 \| 5.6163  5.259 \| 5.5721  4.813 \| \|  \| 7 \| 4.8939  4.288 \| 6.4314  6.168 \| 4.9226  4.411 \| 5.2711  3.761 \| \|  \| 7.4 \| 5.8958  5.091 \| 6.3424  5.980 \| 5.5006  4.546 \| 4.9853  4.184 \| \|  \| 8.1 \| 5.0792  4.984 \| 6.3153  5.363 \| 5.4960  4.768 \| 5.6021  4.784 \| \|  \|  \|  \|  \|  \|  \| |
| --- | --- | --- | --- | --- | --- | --- | --- | --- | --- | --- | --- | --- | --- | --- | --- | --- | --- | --- | --- | --- | --- | --- | --- | --- | --- | --- | --- | --- | --- | --- | --- | --- | --- | --- | --- | --- | --- | --- | --- | --- | --- | --- | --- | --- | --- | --- | --- | --- | --- | --- | --- | --- | --- | --- | --- | --- | --- | --- | --- | --- | --- | --- | --- | --- | --- | --- | --- | --- | --- | --- | --- | --- | --- | --- | --- | --- | --- | --- | --- | --- | --- | --- | --- | --- | --- | --- | --- | --- | --- | --- | --- | --- | --- | --- | --- | --- |

^1^CFU values were log transformed

(± SD) Standard deviation

# Table S20. Effect of pH on mean biofilm biomass^1^ on PS^2^ by *V. parahaemolyticus* and *V. vulnificus*.

| \| **Species** \| **pH** \| **Nutrient Rich (25°C)**  **(± SD)** \| **Nutrient Rich (37°C)**  **(± SD)** \| **Nutrient Starved (25°C) (± SD)** \| **Nutrient Starved (37°C) (± SD)** \| \| --- \| --- \| --- \| --- \| --- \| --- \| \| *V. parahaemolyticus* \| 3 \| 0.0013  0.0012 \| 0.0006  0.0007 \| 0.0038  0.0008 \| 0.0021  0.0017 \| \|  \| 4 \| 0.0009  0.0015 \| 0.0000  0.0000 \| 0.0040  0.0019 \| 0.0068  0.0073 \| \|  \| 5 \| 0.0049  0.0055 \| 0.0015  0.0025 \| 0.0076  0.0030 \| 0.0046  0.0037 \| \|  \| 6 \| 0.0047  0.0048 \| 0.0023  0.0037 \| 0.0089  0.0015 \| 0.0064  0.0106 \| \|  \| 7 \| 0.0039  0.0046 \| 0.0016  0.0021 \| 0.0088  0.0055 \| 0.0016  0.0011 \| \|  \| 7.4 \| 0.0053  0.0051 \| 0.0016  0.0026 \| 0.0048  0.0014 \| 0.0024  0.0002 \| \|  \| 8.1 \| 0.0084  0.0030 \| 0.0020  0.0014 \| 0.0047  0.0019 \| 0.0037  0.0011 \| \| *V. vulnificus* \| 3 \| 0.0017  0.0021 \| 0.0015  0.0013 \| 0.0001  0.0002 \| 0.0016  0.0008 \| \|  \| 4 \| 0.0004  0.0003 \| 0.0008  0.0009 \| 0.0001  0.0001 \| 0.0024  0.0010 \| \|  \| 5 \| 0.0031  0.0038 \| 0.0031  0.0011 \| 0.0003  0.0004 \| 0.0056  0.0032 \| \|  \| 6 \| 0.0013  0.0000 \| 0.0028  0.0010 \| 0.0021  0.0019 \| 0.0062  0.0021 \| \|  \| 7 \| 0.0027  0.0047 \| 0.0022  0.0012 \| 0.0004  0.0003 \| 0.0020  0.0005 \| \|  \| 7.4 \| 0.0020  0.0017 \| 0.0015  0.0003 \| 0.0012  0.0019 \| 0.0017  0.0014 \| \|  \| 8.1 \| 0.0058  0.0004 \| 0.0021  0.0017 \| 0.0010  0.0007 \| 0.0008  0.0002 \| |
| --- | --- | --- | --- | --- | --- | --- | --- | --- | --- | --- | --- | --- | --- | --- | --- | --- | --- | --- | --- | --- | --- | --- | --- | --- | --- | --- | --- | --- | --- | --- | --- | --- | --- | --- | --- | --- | --- | --- | --- | --- | --- | --- | --- | --- | --- | --- | --- | --- | --- | --- | --- | --- | --- | --- | --- | --- | --- | --- | --- | --- | --- | --- | --- | --- | --- | --- | --- | --- | --- | --- | --- | --- | --- | --- | --- | --- | --- | --- | --- | --- | --- | --- | --- | --- | --- | --- | --- | --- | --- | --- |

^1^Optical density at 570 nm

^2^Biomass values divided by total microplastic surface area (14 mm^2^)

(± SD) Standard deviation

Table S21. Effect of pH, temperature, and nutrient availability on *V. parahaemolyticus* and *V. vulnificus* CFUs^1^ on PS^2^.

| \| **Species** \| **pH** \| **Nutrient Rich (25°C)**  **(± SD)** \| **Nutrient Rich (37°C)**  **(± SD)** \| **Nutrient Starved (25°C) (± SD)** \| **Nutrient Starved (37°C) (± SD)** \| \| --- \| --- \| --- \| --- \| --- \| --- \| \| *V. parahaemolyticus* \| 3 \| 0.0000  0.0000 \| 0.0000  0.0000 \| 0.0000  0.0000 \| 0.0000  0.0000 \| \|  \| 4 \| 4.3021  3.1802 \| 0.0000  0.0000 \| 4.6927  3.7943 \| 3.9208  3.3143 \| \|  \| 5 \| 4.3499  3.9012 \| 4.8953  3.8539 \| 5.9531  4.9878 \| 4.8255  3.8830 \| \|  \| 6 \| 3.9874  3.1721 \| 3.6842  2.5948 \| 4.6969  4.0254 \| 4.4907  3.7048 \| \|  \| 7 \| 3.9306  2.9783 \| 3.8539  3.2764 \| 4.8953  4.1549 \| 4.5643  4.0754 \| \|  \| 7.4 \| 3.8495  2.9967 \| 4.0202  3.4604 \| 4.7251  3.3143 \| 4.8466  3.8063 \| \|  \| 8.1 \| 4.7385  3.9936 \| 4.4559  3.5150 \| 4.3680  3.4604 \| 4.7251  4.1039 \| \| *V. vulnificus* \| 3 \| 0.0000  0.0000 \| 0.0000  0.0000 \| 0.0000  0.0000 \| 0.0000  0.0000 \| \|  \| 4 \| 1.8081  0.8539 \| 0.0000  0.0000 \| 2.0395  1.4733 \| 2.3010  1.2764 \| \|  \| 5 \| 2.3768  2.3610 \| 3.7385  3.1451 \| 3.8081  2.9678 \| 3.7606  3.1551 \| \|  \| 6 \| 3.7712  3.5106 \| 4.2593  3.8719 \| 4.1469  2.8546 \| 3.9599  2.7331 \| \|  \| 7 \| 2.3768  1.6153 \| 2.9485  2.3686 \| 3.9450  2.6153 \| 3.3545  2.7614 \| \|  \| 7.4 \| 3.6799  3.5404 \| 3.9711  3.2440 \| 3.9895  3.5080 \| 3.9379  3.3674 \| \|  \| 8.1 \| 2.4061  1.9819 \| 2.2341  1.4108 \| 3.9082  3.0379 \| 4.0516  3.2702 \| \|  \|  \|  \|  \|  \|  \| |
| --- | --- | --- | --- | --- | --- | --- | --- | --- | --- | --- | --- | --- | --- | --- | --- | --- | --- | --- | --- | --- | --- | --- | --- | --- | --- | --- | --- | --- | --- | --- | --- | --- | --- | --- | --- | --- | --- | --- | --- | --- | --- | --- | --- | --- | --- | --- | --- | --- | --- | --- | --- | --- | --- | --- | --- | --- | --- | --- | --- | --- | --- | --- | --- | --- | --- | --- | --- | --- | --- | --- | --- | --- | --- | --- | --- | --- | --- | --- | --- | --- | --- | --- | --- | --- | --- | --- | --- | --- | --- | --- | --- | --- | --- | --- | --- | --- |

^1^CFU values were log transformed

^2^CFU values divided by total coupon surface area (14 mm^2^)

(± SD) Standard deviation

# Table S22. Effect of pH on mean cell colonization^1^ on microplates from PS by *V. parahaemolyticus* and *V. vulnificus*.

| \| **Species** \| **pH** \| **Nutrient Rich (25°C)**  **(± SD)** \| **Nutrient Rich (37°C)**  **(± SD)** \| **Nutrient Starved (25°C) (± SD)** \| **Nutrient Starved (37°C) (± SD)** \| \| --- \| --- \| --- \| --- \| --- \| --- \| \| *V. parahaemolyticus*# \| 3 \| 0.002  0.007 \| 0.004  0.007 \| 0.001  0.000 \| 0.003  0.007 \| \|  \| 4 \| 0.011  0.006 \| 0.005  0.018 \| 0.003  0.006 \| 0.007  0.008 \| \|  \| 5 \| 0.016  0.026 \| 0.022  0.026 \| 0.004  0.005 \| 0.008  0.006 \| \|  \| 6 \| 0.030  0.021 \| 0.113  0.102 \| 0.030  0.018 \| 0.030  0.016 \| \|  \| 7 \| 0.026  0.013 \| 0.026  0.056 \| 0.004  0.002 \| 0.003  0.006 \| \|  \| 7.4 \| 0.024  0.045 \| 0.071  0.061 \| 0.002  0.000 \| 0.008  0.001 \| \|  \| 8.1 \| 0.058  0.006 \| 0.030  0.032 \| 0.006  0.011 \| 0.007  0.003 \| \| *V. vulnificus* \| 3 \| 0.007  0.004 \| 0.010  0.005 \| 0.000  0.001 \| 0.009  0.000 \| \|  \| 4 \| 0.006  0.009 \| 0.019  0.005 \| 0.003  0.005 \| 0.015  0.001 \| \|  \| 5 \| 0.032  0.019 \| 0.033  0.013 \| 0.005  0.004 \| 0.009  0.000 \| \|  \| 6 \| 0.035  0.004 \| 0.152  0.039 \| 0.016  0.041 \| 0.013  0.008 \| \|  \| 7 \| 0.041  0.007 \| 0.123  0.020 \| 0.007  0.006 \| 0.006  0.001 \| \|  \| 7.4 \| 0.065  0.016 \| 0.131  0.025 \| 0.000  0.001 \| 0.012  0.000 \| \|  \| 8.1 \| 0.006  0.011 \| 0.032  0.010 \| 0.006  0.008 \| 0.008  0.002 \| |
| --- | --- | --- | --- | --- | --- | --- | --- | --- | --- | --- | --- | --- | --- | --- | --- | --- | --- | --- | --- | --- | --- | --- | --- | --- | --- | --- | --- | --- | --- | --- | --- | --- | --- | --- | --- | --- | --- | --- | --- | --- | --- | --- | --- | --- | --- | --- | --- | --- | --- | --- | --- | --- | --- | --- | --- | --- | --- | --- | --- | --- | --- | --- | --- | --- | --- | --- | --- | --- | --- | --- | --- | --- | --- | --- | --- | --- | --- | --- | --- | --- | --- | --- | --- | --- | --- | --- | --- | --- | --- | --- |

^1^Optical density at 570 nm

(± SD) Standard deviation

(#) Synergistic significant effect of pH, temperature, and nutrient content on biofilm biomass

Table S23. Effect of pH, temperature, and nutrient availability on *V. parahaemolyticus* and *V. vulnificus* CFUs^1^ dispersed from PS.

| \| **Species** \| **pH** \| **Nutrient Rich (25°C)**  **(± SD)** \| **Nutrient Rich (37°C)**  **(± SD)** \| **Nutrient Starved (25°C) (± SD)** \| **Nutrient Starved (37°C) (± SD)** \| \| --- \| --- \| --- \| --- \| --- \| --- \| \| *V. parahaemolyticus* \| 3 \| 0.0000  0.000 \| 0.0000  0.0000 \| 0.0000  0.0000 \| 0.0000  0.0000 \| \|  \| 4 \| 6.1184  5.1338 \| 0.0000  0.0000 \| 5.9927  5.2765 \| 6.6690  5.7103 \| \|  \| 5 \| 6.2625  5.6069 \| 7.5836  6.3184 \| 6.2553  5.0000 \| 6.4914  5.7955 \| \|  \| 6 \| 6.0768  5.6568 \| 7.3741  7.0050 \| 6.1249  5.5782 \| 6.7188  6.0174 \| \|  \| 7 \| 6.7351  6.4167 \| 7.5315  6.8997 \| 6.1563  5.6541 \| 6.1249  6.0914 \| \|  \| 7.4 \| 6.8633  6.4496 \| 7.4914  6.8997 \| 5.9700  5.3184 \| 6.5836  5.8082 \| \|  \| 8.1 \| 6.4367  5.8234 \| 7.4674  6.7103 \| 6.3680  5.8234 \| 6.1761  5.5570 \| \| *V. vulnificus* \| 3 \| 0.0000  0.0000 \| 0.0000  0.0000 \| 0.0000  0.0000 \| 0.0000  0.0000 \| \|  \| 4 \| 2.8282  2.8648 \| 0.0000  0.0000 \| 3.2937  2.7103 \| 4.0531  2.9542 \| \|  \| 5 \| 4.9853  4.4834 \| 7.1663  6.9687 \| 4.3862  3.8830 \| 5.3490  4.5071 \| \|  \| 6 \| 5.1139  4.6484 \| 5.2131  4.8509 \| 4.9868  4.2893 \| 4.8653  4.4329 \| \|  \| 7 \| 5.5643  5.0559 \| 6.5682  6.1178 \| 5.0000  4.4225 \| 3.8846  3.3184 \| \|  \| 7.4 \| 4.9260  4.5695 \| 7.2005  6.7873 \| 5.4094  4.5071 \| 5.3082  4.7548 \| \|  \| 8.1 \| 5.1461  4.8167 \| 7.4634  6.5086 \| 5.1357  4.7410 \| 5.0000  4.2386 \| \|  \|  \|  \|  \|  \|  \| |
| --- | --- | --- | --- | --- | --- | --- | --- | --- | --- | --- | --- | --- | --- | --- | --- | --- | --- | --- | --- | --- | --- | --- | --- | --- | --- | --- | --- | --- | --- | --- | --- | --- | --- | --- | --- | --- | --- | --- | --- | --- | --- | --- | --- | --- | --- | --- | --- | --- | --- | --- | --- | --- | --- | --- | --- | --- | --- | --- | --- | --- | --- | --- | --- | --- | --- | --- | --- | --- | --- | --- | --- | --- | --- | --- | --- | --- | --- | --- | --- | --- | --- | --- | --- | --- | --- | --- | --- | --- | --- | --- | --- | --- | --- | --- | --- | --- |

^1^CFU values were log transformed

(± SD) Standard deviation

Table S24. Statistical comparison of *V. parahaemolyticus* and *V. vulnificus* biofilm biomass^1^, biofilm cell^2^, cell colonization^1^ and cell dispersal^2^ values on/from LDPE^3^, PP^3^ and PS^4^ in nutrient rich media compared to nutrient starved.

|  | **Surface Type** |  | **Coefficient** | ***p*** |
| --- | --- | --- | --- | --- |
| *V. parahaemolyticus* | **LDPE** | **Biofilm Biomass** | 0.21 | 1.00 |
|  |  | **Biofilm Cells** | -3.5E04 | 1.00 |
|  |  | **Cell Colonization** | -0.11 | 1.00 |
|  |  | **Cell Dispersal** | 6.2E06 | 1.00 |
|  | **PP** | **Biofilm Biomass** | 0.11 | 1.00 |
|  |  | **Biofilm Cells** | -1.3E03 | 1.00 |
|  |  | **Cell Colonization** | 0.04 | 1.00 |
|  |  | **Cell Dispersal** | 5.1E07 | 0.98 |
|  | **PS** | **Biofilm Biomass** | 0.92 | 0.64 |
|  |  | **Biofilm Cells** | 3.2E05 | 1.00 |
|  |  | **Cell Colonization** | 0.03 | 1.00 |
|  |  | **Cell Dispersal** | 2.6E06 | 1.00 |
| *V. vulnificus* | **LDPE** | **Biofilm Biomass** | 2.32E02 | 1.00 |
|  |  | **Biofilm Cells** | -8.8E03 | 0.76 |
|  |  | **Cell Colonization** | 2.02E-02 | 1.00 |
|  |  | **Cell Dispersal** | 1.2E04 | 1.00 |
|  | **PP** | **Biofilm Biomass** | -0.004 | 1.00 |
|  |  | **Biofilm Cells** | -3.8E03 | 1.00 |
|  |  | **Cell Colonization** | 0.029 | 1.00 |
|  |  | **Cell Dispersal** | -6.6E05 | 1.00 |
|  | **PS** | **Biofilm Biomass** | 0.006 | 1.00 |
|  |  | **Biofilm Cells** | -4.1E03 | 1.00 |
|  |  | **Cell Colonization** | 0.02 | 1.00 |
|  |  | **Cell Dispersal** | -2.72E-04 | 1.00 |

^1^Optical density at 570 nm

^2^CFU values were log transformed

^3^Biofilm biomass and CFU values on plastic divided by total coupon surface area (24 mm^2^)

^4^Biofilm biomass and CFU values on plastic divided by total coupon surface area (14 mm^2^)

Table S25. Statistical comparison of *V. parahaemolyticus* and *V. vulnificus* biofilm biomass^1^, biofilm cell^2^, cell colonization^1^ and cell dispersal^2^ values on/from LDPE^3^, PP^3^ and PS^4^ at 37°C compared to 25°C.

|  | **Surface Type** |  | **Coefficient** | ***p*** |
| --- | --- | --- | --- | --- |
| *V. parahaemolyticus* | **LDPE** | **Biofilm Biomass** | 0.22 | 1.00 |
|  |  | **Biofilm Cells** | -7.2E02 | 1.00 |
|  |  | **Cell Colonization** | -0.13 | 1.00 |
|  |  | **Cell Dispersal** | 2.4E07 | 1.00 |
|  | **PP** | **Biofilm Biomass** | -0.02 | 1.00 |
|  |  | **Biofilm Cells** | -1.2E05 | 1.00 |
|  |  | **Cell Colonization** | -0.1 | 0.17 |
|  |  | **Cell Dispersal** | -2.5E07 | 1.00 |
|  | **PS** | **Biofilm Biomass** | 0.07 | 1.00 |
|  |  | **Biofilm Cells** | 1.9E04 | 1.00 |
|  |  | **Cell Colonization** | -0.06 | 0.29 |
|  |  | **Cell Dispersal** | -1.5E07 | 1.00 |
| *V. vulnificus* | **LDPE** | **Biofilm Biomass** | 1.47E-02 | 1.00 |
|  |  | **Biofilm Cells** | 1.3E02 | 1.00 |
|  |  | **Cell Colonization** | -1.11E-05 | 1.00 |
|  |  | **Cell Dispersal** | 2.2E06 | 1.00 |
|  | **PP** | **Biofilm Biomass** | -0.003 | 1.00 |
|  |  | **Biofilm Cells** | -7.7E01 | 1.00 |
|  |  | **Cell Colonization** | 0.005 | 1.00 |
|  |  | **Cell Dispersal** | -2.2E06 | 0.29 |
|  | **PS** | **Biofilm Biomass** | 0.03 | 1.00 |
|  |  | **Biofilm Cells** | 1.4E03 | 1.00 |
|  |  | **Cell Colonization** | 0.006 | 1.00 |
|  |  | **Cell Dispersal** | -1.4E-07 | 0.78 |

^1^Optical density at 570 nm

^2^CFU values were log transformed

^3^Biofilm biomass and CFU values on plastic divided by total coupon surface area (24 mm^2^)

^4^Biofilm biomass and CFU values on plastic divided by total coupon surface area (14 mm^2^)

Table S26. Statistical comparison of effect of pH on *V. parahaemolyticus* and *V. vulnificus* biofilm biomass^1^, biofilm cell^2^, cell colonization^1^ and cell dispersal^2^ values on/from LDPE^3^, PP^3^ and PS^4^.

|  | **Surface Type** |  | **Coefficient** | ***p*** |
| --- | --- | --- | --- | --- |
| *V. parahaemolyticus* | **LDPE** | **Biofilm Biomass** | 0.08* | <0.01 |
|  |  | **Biofilm Cells** | 2.4E04 | 1.00 |
|  |  | **Cell Colonization** | 0.005 | 1.00 |
|  |  | **Cell Dispersal** | 7.1E06 | 1.00 |
|  | **PP** | **Biofilm Biomass** | 0.03 | 0.74 |
|  |  | **Biofilm Cells** | 1.0E04 | 1.00 |
|  |  | **Cell Colonization** | 0.02* | <0.01 |
|  |  | **Cell Dispersal** | 1.3E07* | <0.05 |
|  | **PS** | **Biofilm Biomass** | 0.02 | 0.09 |
|  |  | **Biofilm Cells** | 4.6E03 | 1.00 |
|  |  | **Cell Colonization** | 0.01* | <0.05 |
|  |  | **Cell Dispersal** | 1.0E06 | 1.00 |
| *V. vulnificus* | **LDPE** | **Biofilm Biomass** | 6.34E-03 | 0.87 |
|  |  | **Biofilm Cells** | 3.0E02 | 1.00 |
|  |  | **Cell Colonization** | 8.8E-03* | <0.05 |
|  |  | **Cell Dispersal** | 7.2E04 | 1.00 |
|  | **PP** | **Biofilm Biomass** | 0.001 | 1.00 |
|  |  | **Biofilm Cells** | 4.2E01 | 1.00 |
|  |  | **Cell Colonization** | 0.008* | <0.05 |
|  |  | **Cell Dispersal** | -2.0E04 | 1.00 |
|  | **PS** | **Biofilm Biomass** | 0.008 | 0.59 |
|  |  | **Biofilm Cells** | 4.5E02 | 1.00 |
|  |  | **Cell Colonization** | 0.008 | 0.71 |
|  |  | **Cell Dispersal** | 4.06E-04 | 1.00 |

^1^Optical density at 570 nm

^2^CFU values were log transformed

^3^Biofilm biomass and CFU values on plastic divided by total coupon surface area (24 mm^2^)

^4^Biofilm biomass and CFU values on plastic divided by total coupon surface area (14 mm^2^)

*Significant increase (+) /decrease (-) in biofilm biomass/cell colonization or cell dispersal

Table S27. Statistical comparison of effect of pH on *V. parahaemolyticus* and *V. vulnificus* biofilm biomass^1^, biofilm cell^2^, cell colonization^1^ and cell dispersal^2^ values on/from LDPE^3^, PP^3^ and PS^4^ in nutrient rich media compared to nutrient starved.

|  | **Surface Type** |  | **Coefficient** | ***p*** |
| --- | --- | --- | --- | --- |
| *V. parahaemolyticus* | **LDPE** | **Biofilm Biomass** | -0.07 | 0.053 |
|  |  | **Biofilm Cells** | 3.2E04 | 1.00 |
|  |  | **Cell Colonization** | -0.007 | 1.00 |
|  |  | **Cell Dispersal** | -3.3E06 | 1.00 |
|  | **PP** | **Biofilm Biomass** | -0.02 | 1.00 |
|  |  | **Biofilm Cells** | 1.5E04 | 1.00 |
|  |  | **Cell Colonization** | -0.01 | 0.27 |
|  |  | **Cell Dispersal** | -1.1E07 | 0.37 |
|  | **PS** | **Biofilm Biomass** | -0.01 | 1.00 |
|  |  | **Biofilm Cells** | -3.0E04 | 1.00 |
|  |  | **Cell Colonization** | -0.01 | 0.23 |
|  |  | **Cell Dispersal** | -7.3E05 | 1.00 |
| *V. vulnificus* | **LDPE** | **Biofilm Biomass** | -6.41 | 1.00 |
|  |  | **Biofilm Cells** | 2.2E03 | 0.11 |
|  |  | **Cell Colonization** | -8.43 | 0.27 |
|  |  | **Cell Dispersal** | -2.6E03 | 1.00 |
|  | **PP** | **Biofilm Biomass** | -0.001 | 1.00 |
|  |  | **Biofilm Cells** | 1.3E03 | 0.41 |
|  |  | **Cell Colonization** | -0.008 | 0.28 |
|  |  | **Cell Dispersal** | 6.4E04 | 1.00 |
|  | **PS** | **Biofilm Biomass** | -0.003 | 1.00 |
|  |  | **Biofilm Cells** | 1.3E03 | 1.00 |
|  |  | **Cell Colonization** | -0.007 | 1.00 |
|  |  | **Cell Dispersal** | -2.79E-02 | 1.00 |

^1^Optical density at 570 nm

^2^CFU values were log transformed

^3^Biofilm biomass and CFU values on plastic divided by total coupon surface area (24 mm^2^)

^4^Biofilm biomass and CFU values on plastic divided by total coupon surface area (14 mm^2^)

*Significant increase (+) /decrease (-) in biofilm biomass or cell dispersal

Table S28. Statistical comparison of effect of pH on *V. parahaemolyticus* and *V. vulnificus* biomass^1^, biofilm cell^2^, cell colonization^1^ and cell dispersal^2^ values on/from LDPE^3^, PP^3^ and PS^4^ at 37°C compared to 25°C.

|  | **Surface Type** |  | **Coefficient** | ***p*** |
| --- | --- | --- | --- | --- |
| *V. parahaemolyticus* | **LDPE** | **Biofilm Biomass** | -0.04 | 0.62 |
|  |  | **Biofilm Cells** | -6.1E03 | 1.00 |
|  |  | **Cell Colonization** | 0.04 | 0.11 |
|  |  | **Cell Dispersal** | -3.2E06 | 1.00 |
|  | **PP** | **Biofilm Biomass** | -0.02 | 1.00 |
|  |  | **Biofilm Cells** | 1.8E04 | 1.00 |
|  |  | **Cell Colonization** | 0.03* | ≤0.001 |
|  |  | **Cell Dispersal** | 5.9E06 | 1.00 |
|  | **PS** | **Biofilm Biomass** | -0.02 | 0.34 |
|  |  | **Biofilm Cells** | -3.1E03 | 1.00 |
|  |  | **Cell Colonization** | 0.02* | ≤0.01 |
|  |  | **Cell Dispersal** | 5.3E06 | 0.07 |
| *V. vulnificus* | **LDPE** | **Biofilm Biomass** | -1.6E-03 | 1.00 |
|  |  | **Biofilm Cells** | -3.2E02 | 1.00 |
|  |  | **Cell Colonization** | 1.49E-04 | 1.00 |
|  |  | **Cell Dispersal** | 1.1E06 | 1.00 |
|  | **PP** | **Biofilm Biomass** | 0.001 | 1.00 |
|  |  | **Biofilm Cells** | 2.4E01 | 1.00 |
|  |  | **Cell Colonization** | 0.001 | 1.00 |
|  |  | **Cell Dispersal** | 3.7E05 | 0.051 |
|  | **PS** | **Biofilm Biomass** | -0.006 | 1.00 |
|  |  | **Biofilm Cells** | 3.1E02 | 1.00 |
|  |  | **Cell Colonization** | 0.001 | 1.00 |
|  |  | **Cell Dispersal** | 4.04E-06 | 0.06 |

^1^Optical density at 570 nm

^2^CFU values were log transformed

^3^Biofilm biomass and CFU values on plastic divided by total coupon surface area (24 mm^2^)

^4^Biofilm biomass and CFU values on plastic divided by total coupon surface area (14 mm^2^)

*Significant increase (+) /decrease (-) in biofilm biomass or cell dispersal

Table S29. Statistical comparison of *V. parahaemolyticus* and *V. vulnificus* biofilm biomass^1^, biofilm cell^2^, cell colonization^1^ and cell dispersal^2^ values on/from LDPE^3^, PP^3^ and PS^4^ at 37°C and nutrient starved media compared to 25°C and nutrient rich media.

|  | **Surface Type** |  | **Coefficient** | ***p*** |
| --- | --- | --- | --- | --- |
| *V. parahaemolyticus* | **LDPE** | **Biofilm Biomass** | -0.22 | 1.00 |
|  |  | **Biofilm Cells** | 5.1E04 | 1.00 |
|  |  | **Cell Colonization** | 0.11 | 1.00 |
|  |  | **Cell Dispersal** | -3.8E07 | 1.00 |
|  | **PP** | **Biofilm Biomass** | -0.01 | 1.00 |
|  |  | **Biofilm Cells** | 9.8E05 | 1.00 |
|  |  | **Cell Colonization** | 0.11 | 0.62 |
|  |  | **Cell Dispersal** | 2.2E07 | 1.00 |
|  | **PS** | **Biofilm Biomass** | -0.03 | 1.00 |
|  |  | **Biofilm Cells** | -3.5E05 | 1.00 |
|  |  | **Cell Colonization** | 0.07 | 0.61 |
|  |  | **Cell Dispersal** | 1.4E07 | 1.00 |
| *V. vulnificus* | **LDPE** | **Biofilm Biomass** | -6.19E-03 | 1.09 |
|  |  | **Biofilm Cells** | 6.7E03 | 1.00 |
|  |  | **Cell Colonization** | 9.02E-03 | 1.00 |
|  |  | **Cell Dispersal** | -1.9E06 | 1.00 |
|  | **PP** | **Biofilm Biomass** | 0.002 | 1.00 |
|  |  | **Biofilm Cells** | 5.7E03 | 1.00 |
|  |  | **Cell Colonization** | -0.007 | 1.00 |
|  |  | **Cell Dispersal** | 2.6E06 | 0.9 |
|  | **PS** | **Biofilm Biomass** | 0.03 | 1.00 |
|  |  | **Biofilm Cells** | -1.9E03 | 1.00 |
|  |  | **Cell Colonization** | -0.002 | 1.00 |
|  |  | **Cell Dispersal** | 1.45E-07 | 1.00 |

^1^Optical density at 570 nm

^2^CFU values were log transformed

^3^Biofilm biomass and CFU values on plastic divided by total coupon surface area (24 mm^2^)

^4^Biofilm biomass and CFU values on plastic divided by total coupon surface area (14 mm^2^)

*Significant increase (+) /decrease (-) in biofilm biomass or cell dispersal

Table S30. Statistical comparison of effect of pH on *V. parahaemolyticus* and *V. vulnificus* biofilm biomass^1^, biofilm cell^2^, cell colonization^1^ and cell dispersal^2^ values on/from LDPE^3^, PP^3^ and PS^4^ at 37°C and nutrient starved media compared to 25°C and nutrient rich media.

|  | **Surface Type** |  | **Coefficient** | ***p*** |
| --- | --- | --- | --- | --- |
| *V. parahaemolyticus* | **LDPE** | **Biofilm Biomass** | 0.04 | 1.00 |
|  |  | **Biofilm Cells** | -3.8E04 | 1.00 |
|  |  | **Cell Colonization** | -0.04 | 0.63 |
|  |  | **Cell Dispersal** | 6.8E06 | 1.00 |
|  | **PP** | **Biofilm Biomass** | 0.015 | 1.00 |
|  |  | **Biofilm Cells** | -1.9E4 | 1.00 |
|  |  | **Cell Colonization** | -0.04* | ≤0.01 |
|  |  | **Cell Dispersal** | -4.7E06 | 1.00 |
|  | **PS** | **Biofilm Biomass** | 0.009 | 1.00 |
|  |  | **Biofilm Cells** | 3.9E04 | 1.00 |
|  |  | **Cell Colonization** | -0.02* | ≤0.05 |
|  |  | **Cell Dispersal** | -5.5E06 | 0.35 |
| *V. vulnificus* | **LDPE** | **Biofilm Biomass** | -9.17E-03 | 1.00 |
|  |  | **Biofilm Cells** | -1.3E03 | 1.00 |
|  |  | **Cell Colonization** | -1.11E-03 | 1.00 |
|  |  | **Cell Dispersal** | -1.1E06 | 1.00 |
|  | **PP** | **Biofilm Biomass** | -0.002 | 1.00 |
|  |  | **Biofilm Cells** | -7.5E02 | 1.00 |
|  |  | **Cell Colonization** | -0.001 | 1.00 |
|  |  | **Cell Dispersal** | -5.0E05 | 0.31 |
|  | **PS** | **Biofilm Biomass** | 3.0E-04 | 1.00 |
|  |  | **Biofilm Cells** | -4.6E02 | 1.00 |
|  |  | **Cell Colonization** | -0.002 | 1.00 |
|  |  | **Cell Dispersal** | -4.06E-06 | 0.4 |

^1^Optical density at 570 nm

^2^CFU values were log transformed

^3^Biofilm biomass and CFU values on plastic divided by total coupon surface area (24 mm^2^)

^4^Biofilm biomass and CFU values on plastic divided by total coupon surface area (14 mm^2^)

*Significant increase (+) /decrease (-) in biofilm biomass or cell dispersal

Table S31. Effect of media composition on overall mean biofilm biomass^1^ by *V. parahaemolyticus* and *V. vulnificus*.

|  |  |  |  |  |  |  |
| --- | --- | --- | --- | --- | --- | --- |
| **Species** | **SGF (25°C) (± SD)** | **SGF (37°C) (± SD)** | **SIF (25°C)**  **(± SD)** | **SIF (37°C)**  **(± SD)** | **HPLM (25°C)**  **(± SD)** | **HPLM (37°C)**  **(± SD)** |
| *V. parahaemolyticus* | 0.947  0.296 | 1.106  0.144 | 1.822    0.627 | 1.785  0.339 | 2.980  0.325 | 2.889  0.488 |
| *V. vulnificus* | 0.088  0.059 | 0.137  0.107 | 0.091  0.091 | 0.178  0.111 | 0.491  0.040 | 0.580  0.146 |

^1^Optical density at 570 nm

(± SD) Standard deviation

Table S32. Effect of media composition on mean overall final^1^ *V. parahaemolyticus* and *V. vulnificus* cell dispersal^2^ values.

| **Species** | **SGF (25°C) (± SD)** | **SGF (37°C) (± SD)** | **SIF (25°C)**  **(± SD)** | **SIF (37°C)**  **(± SD)** | **HPLM (25°C)**  **(± SD)** | **HPLM (37°C)**  **(± SD)** |
| --- | --- | --- | --- | --- | --- | --- |
| *V. parahaemolyticus* | 0.002  0.001 | 0.000  0.000 | 0.000  0.000 | 0.000  0.000 | 0.013  0.012 | 0.008  0.008 |
| *V. vulnificus* | 0.000  0.000 | 0.000  0.000 | 0.000  0.000 | 0.000  0.000 | 0.030  0.000 | 0.059  0.004 |

^1^Original starting values were subtracted from final values after exposure.

^2^Optical density at 600 nm

(± SD) Standard deviation

Table S33. Statistical comparison of *V. parahaemolyticus* and *V. vulnificus* overall biofilm biomass^1^ and cell dispersal^2^ values in different simulated human medias at 37°C compared to 25°C.

|  | **Media Type** |  | **Coefficient** | ***p*** |
| --- | --- | --- | --- | --- |
| *V. parahaemolyticus* | **SGF** | **Biofilm Biomass** | -0.06 | 1.00 |
|  |  | **Cell Dispersal** | -0.02 | 1.00 |
|  | **SIF** | **Biofilm Biomass** | -0.5 | 1.00 |
|  |  | **Cell Dispersal** | -0.02 | 1.00 |
|  | **HPLM** | **Biofilm Biomass** | -0.12 | 1.00 |
|  |  | **Cell Dispersal** | -0.03 | 1.00 |
| *V. vulnificus* | **SGF** | **Biofilm Biomass** | -0.05 | 1.00 |
|  |  | **Cell Dispersal** | -0.04 | 1.00 |
|  | **SIF** | **Biofilm Biomass** | -0.05 | 1.00 |
|  |  | **Cell Dispersal** | -0.04 | 1.00 |
|  | **HPLM** | **Biofilm Biomass** | 0.11 | 1.00 |
|  |  | **Cell Dispersal** | -0.01 | 1.00 |

^1^Optical density at 570 nm

^2^Optical density at 600 nm

*Significant increase (+) /decrease (-) in biofilm biomass or cell dispersal

Table S34. Statistical comparison of *V. parahaemolyticus* and *V. vulnificus* overall biofilm biomass^1^ and cell dispersal^2^ values in different simulated human medias compared to respective MSYE with similar pH.

|  | **Media Type** |  | **Coefficient** | ***p*** |
| --- | --- | --- | --- | --- |
| *V. parahaemolyticus* | **SGF** | **Biofilm Biomass** | -0.47 | 1.00 |
|  |  | **Cell Dispersal** | -0.04 | 1.00 |
|  | **SIF** | **Biofilm Biomass** | -0.75 | 1.00 |
|  |  | **Cell Dispersal** | -0.04 | 1.00 |
|  | **HPLM** | **Biofilm Biomass** | 1.46 | 0.35 |
|  |  | **Cell Dispersal** | -0.03 | 1.00 |
| *V. vulnificus* | **SGF** | **Biofilm Biomass** | -0.08 | 1.00 |
|  |  | **Cell Dispersal** | -0.03 | 1.00 |
|  | **SIF** | **Biofilm Biomass** | -0.11 | 1.00 |
|  |  | **Cell Dispersal** | -0.03 | 1.00 |
|  | **HPLM** | **Biofilm Biomass** | 0.37* | ≤0.05 |
|  |  | **Cell Dispersal** | -0.001 | 1.00 |

^1^Optical density at 570 nm

^2^Optical density at 600 nm

*Significant increase (+) /decrease (-) in biofilm biomass or cell dispersal

Table S35. Statistical comparison of *V. parahaemolyticus* and *V. vulnificus* overall biofilm biomass^1^ and cell dispersal^2^ values in different simulated human medias at 37°C compared to MSYE and 25°C.

|  | **Media Type** |  | **Coefficient** | ***p*** |
| --- | --- | --- | --- | --- |
| *V. parahaemolyticus* | **SGF** | **Biofilm Biomass** | -0.06 | 1.00 |
|  |  | **Cell Dispersal** | -0.03 | 1.00 |
|  | **SIF** | **Biofilm Biomass** | -0.51 | 1.00 |
|  |  | **Cell Dispersal** | -0.02 | 1.00 |
|  | **HPLM** | **Biofilm Biomass** | -0.13 | 1.00 |
|  |  | **Cell Dispersal** | -0.03 | 1.00 |
| *V. vulnificus* | **SGF** | **Biofilm Biomass** | -0.06 | 1.00 |
|  |  | **Cell Dispersal** | -0.04 | 1.00 |
|  | **SIF** | **Biofilm Biomass** | -0.06 | 1.00 |
|  |  | **Cell Dispersal** | -0.04 | 1.00 |
|  | **HPLM** | **Biofilm Biomass** | 0.01 | 1.00 |
|  |  | **Cell Dispersal** | -0.01 | 1.00 |

^1^Optical density at 570 nm

^2^Optical density at 600 nm

Table S36. Statistical comparison of *V. vulnificus* overall biofilm biomass^1^ and cell dispersal^2^ values in different simulated human medias compared to *V. parahaemolyticus*.

|  | **Media Type** |  | **Coefficient** | ***p*** |
| --- | --- | --- | --- | --- |
| *V. vulnificus* | **SGF** | **Biofilm Biomass** | -1.4* | ≤0.01 |
|  |  | **Cell Dispersal** | -0.01 | 1.00 |
|  | **SIF** | **Biofilm Biomass** | -1.4* | ≤0.01 |
|  |  | **Cell Dispersal** | -0.01 | 1.00 |
|  | **HPLM** | **Biofilm Biomass** | -1.4* | ≤0.01 |
|  |  | **Cell Dispersal** | -0.02 | 1.00 |

^1^Optical density at 570 nm

^2^Optical density at 600 nm

*Significant increase (+) /decrease (-) in biofilm biomass or cell dispersal

Table S37. Statistical comparison of *V. vulnificus* overall biofilm biomass^1^ and cell dispersal^2^ values in different simulated human medias at 37 °C compared to *V. parahaemolyticus* and 25°C.

|  | **Media Type** |  | **Coefficient** | ***p*** |
| --- | --- | --- | --- | --- |
| *V. vulnificus* | **SGF** | **Biofilm Biomass** | 0.04 | 1.00 |
|  |  | **Cell Dispersal** | 0.02 | 1.00 |
|  | **SIF** | **Biofilm Biomass** | 0.04 | 1.00 |
|  |  | **Cell Dispersal** | 0.02 | 1.00 |
|  | **HPLM** | **Biofilm Biomass** | 0.04 | 1.00 |
|  |  | **Cell Dispersal** | 0.02 | 1.00 |

^1^Optical density at 570 nm

^2^Optical density at 600 nm

*Significant increase (+) /decrease (-) in biofilm biomass or cell dispersal

Table S38. Statistical comparison of *V. vulnificus* overall biofilm biomass^1^ and cell dispersal^2^ values in different simulated human medias compared to MSYE and *V. parahaemolyticus*.

|  | **Media Type** |  | **Coefficient** | ***p*** |
| --- | --- | --- | --- | --- |
| *V. vulnificus* | **SGF** | **Biofilm Biomass** | 0.4 | 1.00 |
|  |  | **Cell Dispersal** | 0.01 | 1.00 |
|  | **SIF** | **Biofilm Biomass** | 0.6 | 1.00 |
|  |  | **Cell Dispersal** | 0.01 | 1.00 |
|  | **HPLM** | **Biofilm Biomass** | -1.1 | 0.52 |
|  |  | **Cell Dispersal** | 0.03 | 1.00 |

^1^Optical density at 570 nm

^2^Optical density at 600 nm

*Significant increase (+) /decrease (-) in biofilm biomass or cell dispersal

Table S39. Statistical comparison of *V. vulnificus* overall biofilm biomass^1^ and cell dispersal^2^ values in different simulated human medias at 37°C compared to MSYE, 25°C and *V. parahaemolyticus*.

|  | **Media Type** |  | **Coefficient** | ***p*** |
| --- | --- | --- | --- | --- |
| *V. vulnificus* | **SGF** | **Biofilm Biomass** | 2.5E-04 | 1.00 |
|  |  | **Cell Dispersal** | -0.02 | 1.00 |
|  | **SIF** | **Biofilm Biomass** | 0.45 | 1.00 |
|  |  | **Cell Dispersal** | -0.02 | 1.00 |
|  | **HPLM** | **Biofilm Biomass** | 0.14 | 1.00 |
|  |  | **Cell Dispersal** | 0.01 | 1.00 |

^1^Optical density at 570 nm

^2^Optical density at 600 nm

*Significant increase (+) /decrease (-) in biofilm biomass or cell dispersal

Table S40. Effect of media composition on *V. parahaemolyticus* and *V. vulnificus* overall biofilm metabolic activity^1^.

| **Species** | **Time (mins)** | **SGF** | **SIF** | **HPLM** |
| --- | --- | --- | --- | --- |
| *V. parahaemolyticus* | 0 | 69553 | 80945 | 52938 |
|  | 15 | 1 | 3943 | 332720 |
|  | 30 | 1 | 5538 | 404464 |
|  | 45 | 1 | 9409 | 318552 |
|  | 60 | 24 | 13741 | 244572 |
|  | 75 | 3 | 17750 | 208055 |
|  | 90 | 82 | 20823 | 169183 |
|  | 105 | 88 | 23614 | 147350 |
|  | 120 | 40 | 25607 | 126688 |
|  | 135 | 174 | 31055 | 112723 |
|  | 150 | 77 | 30834 | 98846 |
|  | 165 | 362 | 35176 | 89673 |
|  | 180 | 89 | 34803 | 82599 |
|  | 195 | 429 | 40118 | 75411 |
|  | 210 | 420 | 40754 | 69570 |
|  | 225 | 459 | 43518 | 64180 |
|  | 240 | 951 | 41949 | 57442 |
| *V. vulnificus* | 0 | 610318 | 250145 | 69480 |
|  | 15 | 1584 | 13677 | 186077 |
|  | 30 | 1 | 11518 | 243303 |
|  | 45 | 1 | 11692 | 323263 |
|  | 60 | 1 | 12774 | 317020 |
|  | 75 | 1 | 13360 | 259164 |
|  | 90 | 1 | 14338 | 225203 |
|  | 105 | 1 | 15633 | 206583 |
|  | 120 | 1 | 12691 | 186356 |
|  | 135 | 1 | 15726 | 172002 |
|  | 150 | 1 | 13942 | 149915 |
|  | 165 | 1 | 22481 | 136072 |
|  | 180 | 1 | 13753 | 122840 |
|  | 195 | 1 | 17789 | 111703 |
|  | 210 | 1 | 17098 | 103603 |
|  | 225 | 1 | 17161 | 97785 |
|  | 240 | 1 | 14964 | 91554 |
|  |  |  |  |  |

^1^RFU values were divided by corresponding optical density at 600 nm values for normalization.

Table S41. Effect of media composition on mean biofilm biomass^1^ on LDPE^2^ by *V. parahaemolyticus* and *V. vulnificus*.

|  |  |  |  |  |  |  |
| --- | --- | --- | --- | --- | --- | --- |
| **Species** | **SGF (25°C) (± SD)** | **SGF (37°C) (± SD)** | **SIF (25°C)**  **(± SD)** | **SIF (37°C)**  **(± SD)** | **HPLM (25°C)**  **(± SD)** | **HPLM (37°C)**  **(± SD)** |
| *V. parahaemolyticus* | 0.0218  0.0077 | 0.0091  0.0036 | 0.0220  0.0085 | 0.0169  0.0059 | 0.0158  0.0077 | 0.0057  0.0071 |
| *V. vulnificus* | 0.0005  0.0000 | 0.0004  0.0001 | 0.0003  0.0001 | 0.0001  0.0001 | 0.0028  0.0017 | 0.0056  0.0016 |

^1^Optical density at 570 nm

^2^Biomass values divided by total microplastic surface area (24 mm^2^)

(± SD) Standard deviation

Table S42. Effect of media composition on mean CFUs^1^ on LDPE^2^ by *V. parahaemolyticus* and *V. vulnificus*.

| \| **Species** \| **SGF (25°C) (± SD)** \| **SGF (37°C) (± SD)** \| **SIF (25°C)**  **(± SD)** \| **SIF (37°C)**  **(± SD)** \| **HPLM (25°C)**  **(± SD)** \| **HPLM (37°C)**  **(± SD)** \| \| --- \| --- \| --- \| --- \| --- \| --- \| --- \| \| *V. parahaemolyticus* \| 0.0000  0.0000 \| 0.0000  0.0000 \| 4.6892  4.4200 \| 0.0000  0.0000 \| 5.8985  5.3655 \| 5.4851  4.3129 \| \| *V. vulnificus* \| 0.0000  0.0000 \| 0.0000  0.0000 \| 0.0000  0.0000 \| 0.0000  0.0000 \| 4.1680  3.6705 \| 3.8669  3.1653 \| |
| --- | --- | --- | --- | --- | --- | --- | --- | --- | --- | --- | --- | --- | --- | --- | --- | --- | --- | --- | --- | --- | --- |

^1^CFU values were log transformed

^2^CFU values divided by total coupon surface area (24 mm^2^)

(± SD) Standard deviation

# Table S43. Effect of media composition on mean cell colonization^1^ on microplates from LDPE by *V. parahaemolyticus* and *V. vulnificus*.

| **Species** | **SGF (25°C) (± SD)** | **SGF (37°C) (± SD)** | **SIF (25°C)**  **(± SD)** | **SIF (37°C)**  **(± SD)** | **HPLM (25°C)**  **(± SD)** | **HPLM (37°C)**  **(± SD)** |
| --- | --- | --- | --- | --- | --- | --- |
| *V. parahaemolyticus* | 0.0756  0.0344 | 0.0591  0.0090 | 0.0293  0.0103 | 0.0549  0.0154 | 0.0682  0.0083 | 0.0369  0.0228 |
| *V. vulnificus* | 0.0009  0.0016 | 0.0252  0.0112 | 0.0000  0.0000 | 0.0220  0.0043 | 0.1455  0.1023 | 0.2182  0.0739 |

^1^Optical density at 570 nm

(± SD) Standard deviation

Table S44. Effect of media composition on mean CFUs^1^ dispersed from LDPE by *V. parahaemolyticus* and *V. vulnificus*.

| **Species** | **SGF (25°C) (± SD)** | **SGF (37°C) (± SD)** | **SIF (25°C)**  **(± SD)** | **SIF (37°C)**  **(± SD)** | **HPLM (25°C)**  **(± SD)** | **HPLM (37°C)**  **(± SD)** |
| --- | --- | --- | --- | --- | --- | --- |
| *V. parahaemolyticus* | 0.0000  0.0000 | 0.0000  0.0000 | 5.0374  4.1799 | 0.0000  0.0000 | 7.4260  6.6065 | 7.5984  6.5455 |
| *V. vulnificus* | 0.0000  0.0000 | 0.0000  0.0000 | 0.0000  0.0000 | 0.0000  0.0000 | 5.8902  5.3334 | 5.9853  5.6065 |

^1^CFU values were log transformed

(± SD) Standard deviation

Table S45. Effect of media composition on mean biofilm biomass^1^ on PP^2^ by *V. parahaemolyticus* and *V. vulnificus*.

| \|  \|  \|  \|  \|  \|  \|  \| \| --- \| --- \| --- \| --- \| --- \| --- \| --- \| \| **Species** \| **SGF (25°C) (± SD)** \| **SGF (37°C) (± SD)** \| **SIF (25°C)**  **(± SD)** \| **SIF (37°C)**  **(± SD)** \| **HPLM (25°C)**  **(± SD)** \| **HPLM (37°C)**  **(± SD)** \| \| *V. parahaemolyticus* \| 0.0130  0.0025 \| 0.0058  0.0008 \| 0.0237  0.0048 \| 0.0086  0.0012 \| 0.0201  0.0038 \| 0.0102  0.0086 \| \| *V. vulnificus* \| 0.0001  0.0002 \| 0.0002  0.0001 \| 0.0000  0.0000 \| 0.0000  0.0000 \| 0.0006  0.0005 \| 0.0018  0.0004 \| |
| --- | --- | --- | --- | --- | --- | --- | --- | --- | --- | --- | --- | --- | --- | --- | --- | --- | --- | --- | --- | --- | --- | --- | --- | --- | --- | --- | --- | --- |

^1^Optical density at 570 nm

^2^Biomass values divided by total microplastic surface area (24 mm^2^)

(± SD) Standard deviation

Table S46. Effect of media composition on mean CFUs^1^ on PP^2^ by *V. parahaemolyticus* and *V. vulnificus*.

| **Species** | **SGF (25°C) (± SD)** | **SGF (37°C) (± SD)** | **SIF (25°C)**  **(± SD)** | **SIF (37°C)**  **(± SD)** | **HPLM (25°C)**  **(± SD)** | **HPLM (37°C)**  **(± SD)** |
| --- | --- | --- | --- | --- | --- | --- |
| *V. parahaemolyticus* | 0.0000  0.0000 | 0.0000  0.0000 | 3.6572  2.7997 | 0.0000  0.0000 | 5.3245  4.5749 | 5.3930  4.0206 |
| *V. vulnificus* | 0.0000  0.0000 | 0.0000  0.0000 | 0.0000  0.0000 | 0.0000  0.0000 | 3.2033  2.4432 | 3.3705  2.6205 |

^1^CFU values were log transformed

^2^CFU values divided by total coupon surface area (24 mm^2^)

(± SD) Standard deviation

# Table S47. Effect of media composition on mean cell colonization^1^ on microplates from PP by *V. parahaemolyticus* and *V. vulnificus*.

| **Species** | **SGF (25°C) (± SD)** | **SGF (37°C) (± SD)** | **SIF (25°C)**  **(± SD)** | **SIF (37°C)**  **(± SD)** | **HPLM (25°C)**  **(± SD)** | **HPLM (37°C)**  **(± SD)** |
| --- | --- | --- | --- | --- | --- | --- |
| *V. parahaemolyticus* | 0.0476  0.0039 | 0.0378  0.0162 | 0.0340  0.0033 | 0.0444  0.0045 | 0.0482  0.0173 | 0.0381  0.0258 |
| *V. vulnificus* | 0.0000  0.0000 | 0.0225  0.0069 | 0.0000  0.0000 | 0.0482  0.0501 | 0.0158  0.0075 | 0.0436  0.0080 |

^1^Optical density at 570 nm

(± SD) Standard deviation

Table S48. Effect of media composition on mean CFUs^1^ dispersed from PP by *V. parahaemolyticus* and *V. vulnificus*.

| **Species** | **SGF (25°C) (± SD)** | **SGF (37°C) (± SD)** | **SIF (25°C)**  **(± SD)** | **SIF (37°C)**  **(± SD)** | **HPLM (25°C)**  **(± SD)** | **HPLM (37°C)**  **(± SD)** |
| --- | --- | --- | --- | --- | --- | --- |
| *V. parahaemolyticus* | 0.0000  0.0000 | 0.0000  0.0000 | 6.9395  6.1288 | 0.0000  0.0000 | 7.2710  6.1840 | 7.4819  6.7410 |
| *V. vulnificus* | 0.0000  0.0000 | 0.0000  0.0000 | 0.0000  0.0000 | 0.0000  0.0000 | 4.6056  3.9755 | 5.0334  3.9842 |

^1^CFU values were log transformed

(± SD) Standard deviation

Table S49. Effect of media composition on mean biofilm biomass^1^ on PS^2^ by *V. parahaemolyticus* and *V. vulnificus*.

| **Species** | **SGF (25°C) (± SD)** | **SGF (37°C) (± SD)** | **SIF (25°C)**  **(± SD)** | **SIF (37°C)**  **(± SD)** | **HPLM (25°C)**  **(± SD)** | **HPLM (37°C)**  **(± SD)** |
| --- | --- | --- | --- | --- | --- | --- |
| *V. parahaemolyticus* | 0.0087    0.0024 | 0.0132  0.0016 | 0.0065  0.0015 | 0.0129  0.0026 | 0.0256  0.0119 | 0.0234  0.0072 |
| *V. vulnificus* | 0.0065  0.0028 | 0.0014  0.0021 | 0.0083  0.0012 | 0.0124  0.0025 | 0.0021  0.0025 | 0.0139  0.0070 |

^1^Optical density at 570 nm

^2^Biomass values divided by total microplastic surface area (14 mm^2^)

(± SD) Standard deviation

Table S50. Effect of media composition on mean CFUs^1^ on PS^2^ by *V. parahaemolyticus* and *V. vulnificus*.

| **Species** | **SGF (25°C) (± SD)** | **SGF (37°C) (± SD)** | **SIF (25°C)**  **(± SD)** | **SIF (37°C)**  **(± SD)** | **HPLM (25°C)**  **(± SD)** | **HPLM (37°C)**  **(± SD)** |
| --- | --- | --- | --- | --- | --- | --- |
| *V. parahaemolyticus* | 0.0000  0.0000 | 0.0000  0.0000 | 2.1010  1.8713 | 0.0000  0.0000 | 4.3405  3.5557 | 4.6271  3.3143 |
| *V. vulnificus* | 0.0000  0.0000 | 0.0000  0.0000 | 1.6555  1.4604 | 0.0000  0.0000 | 3.9809  3.1819 | 5.9985  5.6387 |

^1^CFU values were log transformed.

^2^CFU values divided by total coupon surface area (14 mm^2^)

(± SD) Standard deviation

# Table S51. Effect of media composition on mean cell colonization^1^ on microplates from PS by *V. parahaemolyticus* and *V. vulnificus*.

| **Species** | **SGF (25°C) (± SD)** | **SGF (37°C) (± SD)** | **SIF (25°C)**  **(± SD)** | **SIF (37°C)**  **(± SD)** | **HPLM (25°C)**  **(± SD)** | **HPLM (37°C)**  **(± SD)** |
| --- | --- | --- | --- | --- | --- | --- |
| *V. parahaemolyticus* | 0.0000  0.0000 | 0.0250  0.0072 | 0.0071  0.0063 | 0.0412  0.0082 | 0.0401  0.0327 | 0.0882  0.0320 |
| *V. vulnificus* | 0.0010  0.0017 | 0.0297  0.0119 | 0.0013  0.0022 | 0.0497  0.0190 | 0.0298  0.0262 | 0.1950  0.1344 |

^1^Optical density at 570 nm

(± SD) Standard deviation

Table S52. Effect of media composition on mean CFUs^1^ dispersed from PS by *V. parahaemolyticus* and *V. vulnificus*.

| \| **Species** \| **SGF (25°C) (± SD)** \| **SGF (37°C) (± SD)** \| **SIF (25°C)**  **(± SD)** \| **SIF (37°C)**  **(± SD)** \| **HPLM (25°C)**  **(± SD)** \| **HPLM (37°C)**  **(± SD)** \| \| --- \| --- \| --- \| --- \| --- \| --- \| --- \| \| *V. parahaemolyticus* \| 0.0000    0.0000 \| 0.0000  0.0000 \| 5.5141  4.8466 \| 0.0000  0.0000 \| 6.1760  5.8406 \| 6.0000  5.7157 \| \| *V. vulnificus* \| 0.0000  0.0000 \| 0.0000    0.0000 \| 0.0000  0.0000 \| 0.0000  0.0000 \| 5.2648  4.4998 \| 5.9378  5.4912 \| |
| --- | --- | --- | --- | --- | --- | --- | --- | --- | --- | --- | --- | --- | --- | --- | --- | --- | --- | --- | --- | --- | --- |

^1^CFU values were log transformed

(± SD) Standard deviation

Table S53. Statistical comparison of *V. parahaemolyticus* and *V. vulnificus* biofilm biomass^1^, biofilm cell^2^, cell colonization^1^ and cell dispersal^2^ values on/from LDPE^3^, PP^3^ and PS^4^ in different simulated human medias compared to respective MSYE with similar pH.

|  | **Surface Type** | **Media Type** |  | **Coefficient** | ***p*** |
| --- | --- | --- | --- | --- | --- |
| *V. parahaemolyticus* | **LDPE** | **SGF** | **Biofilm Biomass** | 0.28 | 0.92 |
|  |  |  | **Biofilm Cells** | -1.6E05 | 1.00 |
|  |  |  | **Cell Colonization** | -0.1 | 1.00 |
|  |  |  | **Cell Dispersal** | -4.0E07 | 1.00 |
|  | **PP** | **SGF** | **Biofilm Biomass** | 0.11 | 1.00 |
|  |  |  | **Biofilm Cells** | -3.4E04 | 1.00 |
|  |  |  | **Cell Colonization** | -0.02 | 1.00 |
|  |  |  | **Cell Dispersal** | -4.2E07 | 1.00 |
|  | **PS** | **SGF** | **Biofilm Biomass** | 0.07 | 0.3 |
|  |  |  | **Biofilm Cells** | -5.2E03 | 1.00 |
|  |  |  | **Cell Colonization** | -0.04 | 1.00 |
|  |  |  | **Cell Dispersal** | -4.2E06 | 1.00 |
|  | **LDPE** | **SIF** | **Biofilm Biomass** | 0.28 | 0.88 |
|  |  |  | **Biofilm Cells** | -1.1E05 | 1.00 |
|  |  |  | **Cell Colonization** | -0.14 | 1.00 |
|  |  |  | **Cell Dispersal** | -4.0E07 | 1.00 |
|  | **PP** | **SIF** | **Biofilm Biomass** | 0.37 | 0.12 |
|  |  |  | **Biofilm Cells** | -3.0E4 | 1.00 |
|  |  |  | **Cell Colonization** | -0.04 | 1.00 |
|  |  |  | **Cell Dispersal** | -3.3E07 | 1.00 |
|  | **PS** | **SIF** | **Biofilm Biomass** | 0.04 | 1.00 |
|  |  |  | **Biofilm Cells** | -5.1E03 | 1.00 |
|  |  |  | **Cell Colonization** | -0.03 | 1.00 |
|  |  |  | **Cell Dispersal** | -3.9E06 | 1.00 |
|  | **LDPE** | **HPLM** | **Biofilm Biomass** | 0.13 | 1.00 |
|  |  |  | **Biofilm Cells** | 6.3E05 | 0.22 |
|  |  |  | **Cell Colonization** | -0.12 | 1.00 |
|  |  |  | **Cell Dispersal** | -1.3E07 | 1.00 |
|  | **PP** | **HPLM** | **Biofilm Biomass** | 0.27 | 0.27 |
|  |  |  | **Biofilm Cells** | 1.7E05 | 1.00 |
|  |  |  | **Cell Colonization** | -0.02 | 1.00 |
|  |  |  | **Cell Dispersal** | -2.4E07 | 1.00 |
|  | **PS** | **HPLM** | **Biofilm Biomass** | 0.31* | ≤0.01 |
|  |  |  | **Biofilm Cells** | 1.6E04 | 0.35 |
|  |  |  | **Cell Colonization** | 2.0E-03 | 1.00 |
|  |  |  | **Cell Dispersal** | -2.7E06 | 1.00 |
| *V. vulnificus* | **LDPE** | **SGF** | **Biofilm Biomass** | -0.02 | 1.00 |
|  |  |  | **Biofilm Cells** | -1.5E02 | 1.00 |
|  |  |  | **Cell Colonization** | -0.03 | 1.00 |
|  |  |  | **Cell Dispersal** | -1.6E05 | 1.00 |
|  | **PP** | **SGF** | **Biofilm Biomass** | -0.007 | 1.00 |
|  |  |  | **Biofilm Cells** | -2.39E02 | 1.00 |
|  |  |  | **Cell Colonization** | -0.02 | 1.00 |
|  |  |  | **Cell Dispersal** | -2.8E05 | 1.00 |
|  | **PS** | **SGF** | **Biofilm Biomass** | 0.06* | ≤0.01 |
|  |  |  | **Biofilm Cells** | -1.6E03 | 1.00 |
|  |  |  | **Cell Colonization** | -0.02 | 1.00 |
|  |  |  | **Cell Dispersal** | -1.3E05 | 1.00 |
|  | **LDPE** | **SIF** | **Biofilm Biomass** | -0.02 | 1.00 |
|  |  |  | **Biofilm Cells** | -1.5E02 | 1.00 |
|  |  |  | **Cell Colonization** | -0.03 | 1.00 |
|  |  |  | **Cell Dispersal** | -1.6E05 | 1.00 |
|  | **PP** | **SIF** | **Biofilm Biomass** | -0.01 | 1.00 |
|  |  |  | **Biofilm Cells** | -2.39E02 | 1.00 |
|  |  |  | **Cell Colonization** | -0.02 | 1.00 |
|  |  |  | **Cell Dispersal** | -2.8E05 | 1.00 |
|  | **PS** | **SIF** | **Biofilm Biomass** | 0.09* | ≤0.01 |
|  |  |  | **Biofilm Cells** | -1.6E03 | 1.00 |
|  |  |  | **Cell Colonization** | -0.02 | 1.00 |
|  |  |  | **Cell Dispersal** | -1.5E05 | 1.00 |
|  | **LDPE** | **HPLM** | **Biofilm Biomass** | 0.04 | 1.00 |
|  |  |  | **Biofilm Cells** | 1.3E04* | ≤0.01 |
|  |  |  | **Cell Colonization** | 0.11 | 0.15 |
|  |  |  | **Cell Dispersal** | 6.1E05 | 1.00 |
|  | **PP** | **HPLM** | **Biofilm Biomass** | 0.006 | 1.00 |
|  |  |  | **Biofilm Cells** | 1.3E03 | 0.76 |
|  |  |  | **Cell Colonization** | -0.003 | 1.00 |
|  |  |  | **Cell Dispersal** | -2.5E05 | 1.00 |
|  | **PS** | **HPLM** | **Biofilm Biomass** | 0.05* | ≤0.05 |
|  |  |  | **Biofilm Cells** | 7.8E03 | 1.00 |
|  |  |  | **Cell Colonization** | 0.01 | 1.00 |
|  |  |  | **Cell Dispersal** | 3.4E04 | 1.00 |

^1^Optical density at 570 nm

^2^CFU values were log transformed

^3^Biofilm biomass and CFU values on plastic divided by total coupon surface area (24 mm^2^)

^4^Biofilm biomass and CFU values on plastic divided by total coupon surface area (14 mm^2^)

*Significant increase (+) /decrease (-) in biofilm biomass or cell dispersal

Table S54. Statistical overall comparison of *V. parahaemolyticus* and *V. vulnificus* biofilm biomass^1^, biofilm cell^2^, cell colonization^1^ and cell dispersal^2^ values on/from LDPE^3^, PP^3^ and PS^4^ at 37°C compared to 25°C.

|  | **Surface Type** |  | **Coefficient** | ***p*** |
| --- | --- | --- | --- | --- |
| *V. parahaemolyticus* | **LDPE** | **Biofilm Biomass** | 0.03 | 1.00 |
|  |  | **Biofilm Cells** | -7.9E04 | 1.00 |
|  |  | **Cell Colonization** | 0.09 | 1.00 |
|  |  | **Cell Dispersal** | -2.3E07 | 1.00 |
|  | **PP** | **Biofilm Biomass** | -0.03 | 1.00 |
|  |  | **Biofilm Cells** | 1.2E05 | 1.00 |
|  |  | **Cell Colonization** | 0.08 | 1.00 |
|  |  | **Cell Dispersal** | 9.7E06 | 1.00 |
|  | **PS** | **Biofilm Biomass** | -0.03 | 1.00 |
|  |  | **Biofilm Cells** | 6.7E02 | 1.00 |
|  |  | **Cell Colonization** | 0.05 | 1.00 |
|  |  | **Cell Dispersal** | 1.7E07 | 1.00 |
| *V. vulnificus* | **LDPE** | **Biofilm Biomass** | 8.0E-03 | 1.00 |
|  |  | **Biofilm Cells** | -1.2E03 | 1.00 |
|  |  | **Cell Colonization** | -2.0E-03 | 1.00 |
|  |  | **Cell Dispersal** | 3.1E06 | 1.00 |
|  | **PP** | **Biofilm Biomass** | 0.01 | 1.00 |
|  |  | **Biofilm Cells** | 1.83E02 | 1.00 |
|  |  | **Cell Colonization** | 0.02 | 1.00 |
|  |  | **Cell Dispersal** | 1.3E06 | 1.00 |
|  | **PS** | **Biofilm Biomass** | -0.004 | 1.00 |
|  |  | **Biofilm Cells** | 1.7E03 | 1.00 |
|  |  | **Cell Colonization** | 0.01 | 1.00 |
|  |  | **Cell Dispersal** | 6.3E06 | 1.00 |

^1^Optical density at 570 nm

^2^CFU values were log transformed

^3^Biofilm biomass and CFU values on plastic divided by total coupon surface area (24 mm^2^)

^4^Biofilm biomass and CFU values on plastic divided by total coupon surface area (14 mm^2^)

*Significant increase (+) /decrease (-) in biofilm biomass or cell dispersal

Table S55. Statistical comparison of *V. parahaemolyticus* and *V. vulnificus* biofilm biomass^1^, biofilm cell^2^, cell colonization^1^ and cell dispersal^2^ values on/from LDPE^3^, PP^3^ and PS^4^ in different simulated human medias at 37°C compared to MSYE and 25°C.

|  | **Surface Type** | **Media Type** |  | **Coefficient** | ***p*** |
| --- | --- | --- | --- | --- | --- |
| *V. parahaemolyticus* | **LDPE** | **SGF** | **Biofilm Biomass** | -0.33 | 1.00 |
|  |  |  | **Biofilm Cells** | 7.9E05 | 1.00 |
|  |  |  | **Cell Colonization** | -0.11 | 1.00 |
|  |  |  | **Cell Dispersal** | 2.3E07 | 1.00 |
|  | **PP** | **SGF** | **Biofilm Biomass** | -0.12 | 1.00 |
|  |  |  | **Biofilm Cells** | -1.2E05 | 1.00 |
|  |  |  | **Cell Colonization** | -0.09 | 1.00 |
|  |  |  | **Cell Dispersal** | -9.7E06 | 1.00 |
|  | **PS** | **SGF** | **Biofilm Biomass** | 0.09 | 0.4 |
|  |  |  | **Biofilm Cells** | -6.7E02 | 1.00 |
|  |  |  | **Cell Colonization** | -0.02 | 1.00 |
|  |  |  | **Cell Dispersal** | -1.7E07 | 1.00 |
|  | **LDPE** | **SIF** | **Biofilm Biomass** | -0.15 | 1.00 |
|  |  |  | **Biofilm Cells** | 3.0E05 | 1.00 |
|  |  |  | **Cell Colonization** | -0.07 | 1.00 |
|  |  |  | **Cell Dispersal** | 2.3E07 | 1.00 |
|  | **PP** | **SIF** | **Biofilm Biomass** | -0.31 | 0.53 |
|  |  |  | **Biofilm Cells** | -1.2E05 | 1.00 |
|  |  |  | **Cell Colonization** | -0.07 | 1.00 |
|  |  |  | **Cell Dispersal** | -1.8E07 | 1.00 |
|  | **PS** | **SIF** | **Biofilm Biomass** | 0.12 | 0.19 |
|  |  |  | **Biofilm Cells** | -8.0E02 | 1.00 |
|  |  |  | **Cell Colonization** | -0.02 | 1.00 |
|  |  |  | **Cell Dispersal** | -1.8E07 | 1.00 |
|  | **LDPE** | **HPLM** | **Biofilm Biomass** | -0.27 | 1.00 |
|  |  |  | **Biofilm Cells** | -4.1E05 | 1.00 |
|  |  |  | **Cell Colonization** | -0.12 | 1.00 |
|  |  |  | **Cell Dispersal** | 3.6E07 | 1.00 |
|  | **PP** | **HPLM** | **Biofilm Biomass** | -0.18 | 1.00 |
|  |  |  | **Biofilm Cells** | -8.3E04 | 1.00 |
|  |  |  | **Cell Colonization** | -0.09 | 1.00 |
|  |  |  | **Cell Dispersal** | 1.9E06 | 1.00 |
|  | **PS** | **HPLM** | **Biofilm Biomass** | 1.0E-03 | 1.00 |
|  |  |  | **Biofilm Cells** | 1.9E04 | 0.57 |
|  |  |  | **Cell Colonization** | -2.0E-03 | 1.00 |
|  |  |  | **Cell Dispersal** | -1.8E07 | 1.00 |
| *V. vulnificus* | **LDPE** | **SGF** | **Biofilm Biomass** | -0.01 | 1.00 |
|  |  |  | **Biofilm Cells** | 1.2E03 | 1.00 |
|  |  |  | **Cell Colonization** | 0.02 | 1.00 |
|  |  |  | **Cell Dispersal** | -3.1E06 | 1.00 |
|  | **PP** | **SGF** | **Biofilm Biomass** | -0.01 | 1.00 |
|  |  |  | **Biofilm Cells** | -1.8E02 | 1.00 |
|  |  |  | **Cell Colonization** | 2.0E-04 | 1.00 |
|  |  |  | **Cell Dispersal** | -1.3E06 | 1.00 |
|  | **PS** | **SGF** | **Biofilm Biomass** | 0.04 | 0.11 |
|  |  |  | **Biofilm Cells** | -1.7E03 | 1.00 |
|  |  |  | **Cell Colonization** | 0.01 | 1.00 |
|  |  |  | **Cell Dispersal** | -6.3E06 | 1.00 |
|  | **LDPE** | **SIF** | **Biofilm Biomass** | -0.01 | 1.00 |
|  |  |  | **Biofilm Cells** | 1.2E03 | 1.00 |
|  |  |  | **Cell Colonization** | 0.02 | 1.00 |
|  |  |  | **Cell Dispersal** | -3.1E06 | 1.00 |
|  | **PP** | **SIF** | **Biofilm Biomass** | -9.0E-03 | 1.00 |
|  |  |  | **Biofilm Cells** | -1.8E02 | 1.00 |
|  |  |  | **Cell Colonization** | 0.02 | 1.00 |
|  |  |  | **Cell Dispersal** | -1.3E06 | 1.00 |
|  | **PS** | **SIF** | **Biofilm Biomass** | 0.06* | ≤0.05 |
|  |  |  | **Biofilm Cells** | -1.7E03 | 1.00 |
|  |  |  | **Cell Colonization** | 0.03 | 1.00 |
|  |  |  | **Cell Dispersal** | -6.3E06 | 1.00 |
|  | **LDPE** | **HPLM** | **Biofilm Biomass** | 0.06 | 1.00 |
|  |  |  | **Biofilm Cells** | -6.1E03 | 0.17 |
|  |  |  | **Cell Colonization** | 0.07 | 1.00 |
|  |  |  | **Cell Dispersal** | -2.9E06 | 1.00 |
|  | **PP** | **HPLM** | **Biofilm Biomass** | 0.04 | 1.00 |
|  |  |  | **Biofilm Cells** | 5.6E02 | 1.00 |
|  |  |  | **Cell Colonization** | 5.00E-03 | 1.00 |
|  |  |  | **Cell Dispersal** | -1.2E06 | 1.00 |
|  | **PS** | **HPLM** | **Biofilm Biomass** | 0.16* | ≤0.001 |
|  |  |  | **Biofilm Cells** | 5.9E04* | ≤0.01 |
|  |  |  | **Cell Colonization** | 0.15 | 0.19 |
|  |  |  | **Cell Dispersal** | -5.7E06 | 1.00 |

^1^Optical density at 570 nm

^2^CFU values were log transformed

^3^Biofilm biomass and CFU values on plastic divided by total coupon surface area (24 mm^2^)

^4^Biofilm biomass and CFU values on plastic divided by total coupon surface area (14 mm^2^)

*Significant increase (+) /decrease (-) in biofilm biomass or cell dispersal

Table S56. Statistical comparison of *V. vulnificus* biofilm biomass^1^, biofilm cell^2^, cell colonization^1^ and cell dispersal^2^ values on/from LDPE^3^, PP^3^ and PS^4^ in different simulated human medias compared to *V. parahaemolyticus*.

|  | **Surface Type** | **Media Type** |  | **Coefficient** | ***p*** |
| --- | --- | --- | --- | --- | --- |
| *V. vulnificus* | **LDPE** | **SGF** | **Biofilm Biomass** | -0.21 | 0.12 |
|  |  |  | **Biofilm Cells** | -1.5E05 | 1.00 |
|  |  |  | **Cell Colonization** | -0.14 | 0.29 |
|  |  |  | **Cell Dispersal** | -4.0E07 | 0.28 |
|  | **PP** | **SGF** | **Biofilm Biomass** | -0.19* | ≤0.05 |
|  |  |  | **Biofilm Cells** | -3.4E04 | 1.00 |
|  |  |  | **Cell Colonization** | -0.05 | 1.00 |
|  |  |  | **Cell Dispersal** | -4.2E07 | 1.00 |
|  | **PS** | **SGF** | **Biofilm Biomass** | -0.019 | 1.00 |
|  |  |  | **Biofilm Cells** | -5.3E03 | 1.00 |
|  |  |  | **Cell Colonization** | -0.02 | 1.00 |
|  |  |  | **Cell Dispersal** | -4.1E06 | 1.00 |
|  | **LDPE** | **SIF** | **Biofilm Biomass** | -0.21 | 0.12 |
|  |  |  | **Biofilm Cells** | -1.3E05 | 1.00 |
|  |  |  | **Cell Colonization** | -0.14 | 0.29 |
|  |  |  | **Cell Dispersal** | -4.0E07 | 0.28 |
|  | **PP** | **SIF** | **Biofilm Biomass** | -0.19* | ≤0.05 |
|  |  |  | **Biofilm Cells** | -3.4E04 | 1.00 |
|  |  |  | **Cell Colonization** | -0.05 | 1.00 |
|  |  |  | **Cell Dispersal** | -4.2E07 | 1.00 |
|  | **PS** | **SIF** | **Biofilm Biomass** | -0.02 | 1.00 |
|  |  |  | **Biofilm Cells** | -3.3E03 | 1.00 |
|  |  |  | **Cell Colonization** | -0.02 | 1.00 |
|  |  |  | **Cell Dispersal** | -4.1E06 | 1.00 |
|  | **LDPE** | **HPLM** | **Biofilm Biomass** | -0.21 | 0.12 |
|  |  |  | **Biofilm Cells** | -1.3E05 | 1.00 |
|  |  |  | **Cell Colonization** | -0.14 | 0.28 |
|  |  |  | **Cell Dispersal** | -4.0E07 | 0.28 |
|  | **PP** | **HPLM** | **Biofilm Biomass** | -0.19* | ≤0.05 |
|  |  |  | **Biofilm Cells** | -3.4E04 | 1.00 |
|  |  |  | **Cell Colonization** | -0.05 | 1.00 |
|  |  |  | **Cell Dispersal** | -4.2E07 | 1.00 |
|  | **PS** | **HPLM** | **Biofilm Biomass** | -0.01 | 1.00 |
|  |  |  | **Biofilm Cells** | -3.5E3 | 1.00 |
|  |  |  | **Cell Colonization** | -0.02 | 1.00 |
|  |  |  | **Cell Dispersal** | -4.1E06 | 1.00 |

^1^Optical density at 570 nm

^2^CFU values were log transformed

^3^Biofilm biomass and CFU values on plastic divided by total coupon surface area (24 mm^2^)

^4^Biofilm biomass and CFU values on plastic divided by total coupon surface area (14 mm^2^)

*Significant increase (+) /decrease (-) in biofilm biomass or cell dispersal

Table S57. Statistical comparison of *V.* biofilm biomass^1^, biofilm cell^2^, cell colonization^1^ and cell dispersal^2^ values on/from LDPE^3^, PP^3^ and PS^4^ at 37 °C compared to *V. parahaemolyticus* and 25°C.

|  | **Surface Type** | **Media Type** |  | **Coefficient** | ***p*** |
| --- | --- | --- | --- | --- | --- |
| *V. vulnificus* | **LDPE** | **SGF** | **Biofilm Biomass** | -0.02 | 1.00 |
|  |  |  | **Biofilm Cells** | 7.6E04 | 1.00 |
|  |  |  | **Cell Colonization** | -0.09 | 1.00 |
|  |  |  | **Cell Dispersal** | 2.6E07 | 1.00 |
|  | **PP** | **SGF** | **Biofilm Biomass** | 0.06 | 1.00 |
|  |  |  | **Biofilm Cells** | -1.1E05 | 1.00 |
|  |  |  | **Cell Colonization** | -0.06 | 1.00 |
|  |  |  | **Cell Dispersal** | -8.4E06 | 1.00 |
|  | **PS** | **SGF** | **Biofilm Biomass** | 0.03 | 1.00 |
|  |  |  | **Biofilm Cells** | 1.1E03 | 1.00 |
|  |  |  | **Cell Colonization** | -0.03 | 1.00 |
|  |  |  | **Cell Dispersal** | -1.1E07 | 1.00 |
|  | **LDPE** | **SIF** | **Biofilm Biomass** | -0.02 | 1.00 |
|  |  |  | **Biofilm Cells** | 7.8E05 | 1.00 |
|  |  |  | **Cell Colonization** | -0.09 | 1.00 |
|  |  |  | **Cell Dispersal** | 2.6E07 | 1.00 |
|  | **PP** | **SIF** | **Biofilm Biomass** | 0.06 | 1.00 |
|  |  |  | **Biofilm Cells** | -1.2E05 | 1.00 |
|  |  |  | **Cell Colonization** | -0.06 | 1.00 |
|  |  |  | **Cell Dispersal** | -8.4E06 | 1.00 |
|  | **PS** | **SIF** | **Biofilm Biomass** | 0.03 | 1.00 |
|  |  |  | **Biofilm Cells** | 1.1E03 | 1.00 |
|  |  |  | **Cell Colonization** | -0.03 | 1.00 |
|  |  |  | **Cell Dispersal** | -1.1E07 | 1.00 |
|  | **LDPE** | **HPLM** | **Biofilm Biomass** | -0.018 | 1.00 |
|  |  |  | **Biofilm Cells** | 7.8E04 | 1.00 |
|  |  |  | **Cell Colonization** | -0.09 | 1.00 |
|  |  |  | **Cell Dispersal** | 2.6E07 | 1.00 |
|  | **PP** | **HPLM** | **Biofilm Biomass** | 0.06 | 0.12 |
|  |  |  | **Biofilm Cells** | -1.1E05 | 1.00 |
|  |  |  | **Cell Colonization** | -0.06 | 1.00 |
|  |  |  | **Cell Dispersal** | -8.4E06 | 1.00 |
|  | **PS** | **HPLM** | **Biofilm Biomass** | 0.03 | 1.00 |
|  |  |  | **Biofilm Cells** | 1.0E03 | 1.00 |
|  |  |  | **Cell Colonization** | -0.03 | 1.00 |
|  |  |  | **Cell Dispersal** | -1.1E07 | 1.00 |

^1^Optical density at 570 nm

^2^CFU values were log transformed

^3^Biofilm biomass and CFU values on plastic divided by total coupon surface area (24 mm^2^)

^4^Biofilm biomass and CFU values on plastic divided by total coupon surface area (14 mm^2^)

*Significant increase (+) /decrease (-) in biofilm biomass or cell dispersal

Table S58. Statistical comparison of *V. vulnificus* biofilm biomass^1^, biofilm cell^2^, cell colonization^1^ and cell dispersal^2^ values on/from LDPE^3^, PP^3^ and PS^4^ in different simulated human medias compared to MSYE and *V. parahaemolyticus*.

|  | **Surface Type** | **Media Type** |  | **Coefficient** | ***p*** |
| --- | --- | --- | --- | --- | --- |
| *V. vulnificus* | **LDPE** | **SGF** | **Biofilm Biomass** | -0.3 | 0.56 |
|  |  |  | **Biofilm Cells** | 1.5E05 | 1.00 |
|  |  |  | **Cell Colonization** | 0.06 | 1.00 |
|  |  |  | **Cell Dispersal** | 4.0E07 | 1.00 |
|  | **PP** | **SGF** | **Biofilm Biomass** | -0.11 | 1.00 |
|  |  |  | **Biofilm Cells** | 3.4E04 | 1.00 |
|  |  |  | **Cell Colonization** | 2.0E-03 | 1.00 |
|  |  |  | **Cell Dispersal** | 4.2E07 | 1.00 |
|  | **PS** | **SGF** | **Biofilm Biomass** | -0.01 | 1.00 |
|  |  |  | **Biofilm Cells** | 3.5E03 | 1.00 |
|  |  |  | **Cell Colonization** | 0.02 | 1.00 |
|  |  |  | **Cell Dispersal** | 4.1E06 | 1.00 |
|  | **LDPE** | **SIF** | **Biofilm Biomass** | -0.3 | 0.52 |
|  |  |  | **Biofilm Cells** | 1.1E05 | 1.00 |
|  |  |  | **Cell Colonization** | 0.11 | 1.00 |
|  |  |  | **Cell Dispersal** | 4.0E07 | 1.00 |
|  | **PP** | **SIF** | **Biofilm Biomass** | -0.4* | ≤0.05 |
|  |  |  | **Biofilm Cells** | 3.0E04 | 1.00 |
|  |  |  | **Cell Colonization** | 0.01 | 1.00 |
|  |  |  | **Cell Dispersal** | 3.3E07 | 1.00 |
|  | **PS** | **SIF** | **Biofilm Biomass** | 0.04 | 0.93 |
|  |  |  | **Biofilm Cells** | 3.4E03 | 1.00 |
|  |  |  | **Cell Colonization** | 0.01 | 1.00 |
|  |  |  | **Cell Dispersal** | 3.7E06 | 1.00 |
|  | **LDPE** | **HPLM** | **Biofilm Biomass** | -0.09 | 1.00 |
|  |  |  | **Biofilm Cells** | -6.2E05 | 0.08 |
|  |  |  | **Cell Colonization** | 0.21 | 0.71 |
|  |  |  | **Cell Dispersal** | 1.4E07 | 1.00 |
|  | **PP** | **HPLM** | **Biofilm Biomass** | -0.03 | 0.12 |
|  |  |  | **Biofilm Cells** | -1.7E05 | 1.00 |
|  |  |  | **Cell Colonization** | 0.02 | 1.00 |
|  |  |  | **Cell Dispersal** | 2.3E07 | 1.00 |
|  | **PS** | **HPLM** | **Biofilm Biomass** | -0.26* | ≤0.001 |
|  |  |  | **Biofilm Cells** | -8.8E03 | 1.00 |
|  |  |  | **Cell Colonization** | 0.01 | 1.00 |
|  |  |  | **Cell Dispersal** | 2.8E06 | 1.00 |

^1^Optical density at 570 nm

^2^CFU values were log transformed

^3^Biofilm biomass and CFU values on plastic divided by total coupon surface area (24 mm^2^)

^4^Biofilm biomass and CFU values on plastic divided by total coupon surface area (14 mm^2^)

*Significant increase (+) /decrease (-) in biofilm biomass or cell dispersal

Table S59. Statistical comparison of *V. vulnificus* biofilm biomass^1^, biofilm cell^2^, cell colonization^1^ and cell dispersal^2^ values on/from LDPE^3^, PP^3^ and PS^4^ in different simulated human medias at 37°C compared to MSYE, 25°C and *V. parahaemolyticus*.

|  | **Surface Type** | **Media Type** |  | **Coefficient** | ***p*** |
| --- | --- | --- | --- | --- | --- |
| *V. vulnificus* | **LDPE** | **SGF** | **Biofilm Biomass** | 0.31 | 1.00 |
|  |  |  | **Biofilm Cells** | -7.9E04 | 1.00 |
|  |  |  | **Cell Colonization** | 0.13 | 1.00 |
|  |  |  | **Cell Dispersal** | -2.7E07 | 1.00 |
|  | **PP** | **SGF** | **Biofilm Biomass** | 0.11 | 1.00 |
|  |  |  | **Biofilm Cells** | 1.2E05 | 1.00 |
|  |  |  | **Cell Colonization** | 0.09 | 1.00 |
|  |  |  | **Cell Dispersal** | 8.4E06 | 1.00 |
|  | **PS** | **SGF** | **Biofilm Biomass** | -0.03 | 1.00 |
|  |  |  | **Biofilm Cells** | -1.1E03 | 1.00 |
|  |  |  | **Cell Colonization** | 0.04 | 1.00 |
|  |  |  | **Cell Dispersal** | 1.1E07 | 1.00 |
|  | **LDPE** | **SIF** | **Biofilm Biomass** | 0.13 | 1.00 |
|  |  |  | **Biofilm Cells** | -2.9E04 | 1.00 |
|  |  |  | **Cell Colonization** | 0.09 | 1.00 |
|  |  |  | **Cell Dispersal** | -2.7E07 | 1.00 |
|  | **PP** | **SIF** | **Biofilm Biomass** | 0.3 | 0.34 |
|  |  |  | **Biofilm Cells** | 1.2E05 | 1.00 |
|  |  |  | **Cell Colonization** | 0.1 | 1.00 |
|  |  |  | **Cell Dispersal** | 1.7E07 | 1.00 |
|  | **PS** | **SIF** | **Biofilm Biomass** | -0.03 | 1.00 |
|  |  |  | **Biofilm Cells** | -9.3E02 | 1.00 |
|  |  |  | **Cell Colonization** | 0.05 | 1.00 |
|  |  |  | **Cell Dispersal** | 1.2E07 | 1.00 |
|  | **LDPE** | **HPLM** | **Biofilm Biomass** | 0.32 | 1.00 |
|  |  |  | **Biofilm Cells** | 4.0E05 | 1.00 |
|  |  |  | **Cell Colonization** | 0.19 | 1.00 |
|  |  |  | **Cell Dispersal** | -4.0E07 | 1.00 |
|  | **PP** | **HPLM** | **Biofilm Biomass** | 0.22 | 0.94 |
|  |  |  | **Biofilm Cells** | 8.4E04 | 1.00 |
|  |  |  | **Cell Colonization** | 0.09 | 1.00 |
|  |  |  | **Cell Dispersal** | -3.2E06 | 1.00 |
|  | **PS** | **HPLM** | **Biofilm Biomass** | 0.16* | ≤0.05 |
|  |  |  | **Biofilm Cells** | 4.0E04* | ≤0.05 |
|  |  |  | **Cell Colonization** | 0.15 | 1.00 |
|  |  |  | **Cell Dispersal** | 1.2E07 | 1.00 |

^1^Optical density at 570 nm

^2^CFU values were log transformed

^3^Biofilm biomass and CFU values on plastic divided by total coupon surface area (24 mm^2^)

^4^Biofilm biomass and CFU values on plastic divided by total coupon surface area (14 mm^2^)

*Significant increase (+) /decrease (-) in biofilm biomass or cell dispersal.

Table S60. Effect of media composition on *V. parahaemolyticus* and *V. vulnificus* biofilm and cell dispersal estimated c-di-GMP concentrations (ng/µL) at 37°C.

| **Species** | **MSYE (Biofilm)**  **(± SD)** | **MSYE (Dispersal)**  **(± SD)** | **SIF**  **(Biofilm)**  **(± SD)** | **SIF**  **(Dispersal)**  **(± SD)** | **HPLM (Biofilm)**  **(± SD)** | **HPLM (Dispersal)**  **(± SD)** |
| --- | --- | --- | --- | --- | --- | --- |
| *V. parahaemolyticus* | 0.15  0.24 | 0.00  0.00 | 4.92  2.64 | 2.78  2.88 | 0.06  0.15 | 0.00  0.00 |
| *V. vulnificus* | 0.44  0.40 | 0.00  0.00 | 6.99  3.76 | 7.61  3.92 | 0.86  0.22 | 0.07  0.17 |

(± SD) Standard deviation

Table S61. Statistical comparison of *V. parahaemolyticus* and *V. vulnificus* c-di-GMP concentrations within biofilms and within dispersal cell states in different medias.

| **Species** | **Cell State** | **Media Comparison** | ***p*** |
| --- | --- | --- | --- |
| *V. parahaemolyticus* | Biofilm | SIF vs MSYE* | ≤0.01 |
|  |  | SIF vs HPLM* | ≤0.01 |
|  |  | MSYE vs HPLM | 0.86 |
|  | Dispersal | SIF vs MSYE | 0.08 |
|  |  | SIF vs HPLM | 0.08 |
|  |  | MSYE vs HPLM | 1.00 |
| *V. vulnificus* | Biofilm | SIF vs MSYE* | ≤0.01 |
|  |  | SIF vs HPLM* | ≤0.01 |
|  |  | MSYE vs HPLM | 0.09 |
|  | Dispersal | SIF vs MSYE* | ≤0.01 |
|  |  | SIF vs HPLM* | ≤0.01 |
|  |  | MSYE vs HPLM | 0.12 |

*Significant difference in c-di-GMP concentrations between medias

Table S62. Statistical comparison of *V. parahaemolyticus* and *V. vulnificus* c-di-GMP concentrations between biofilm and dispersal cell states within different medias.

| **Species** | **Media** | ***p*** |
| --- | --- | --- |
| *V. parahaemolyticus* | MSYE | 0.28 |
|  | SIF | 0.41 |
|  | HPLM | 0.68 |
| *V. vulnificus* | MSYE* | ≤0.05 |
|  | SIF | 1.00 |
|  | HPLM* | ≤0.001 |

*Significant difference in c-di-GMP concentrations between biofilm and dispersal cell states in this media

# Supplementary Figures and Tables


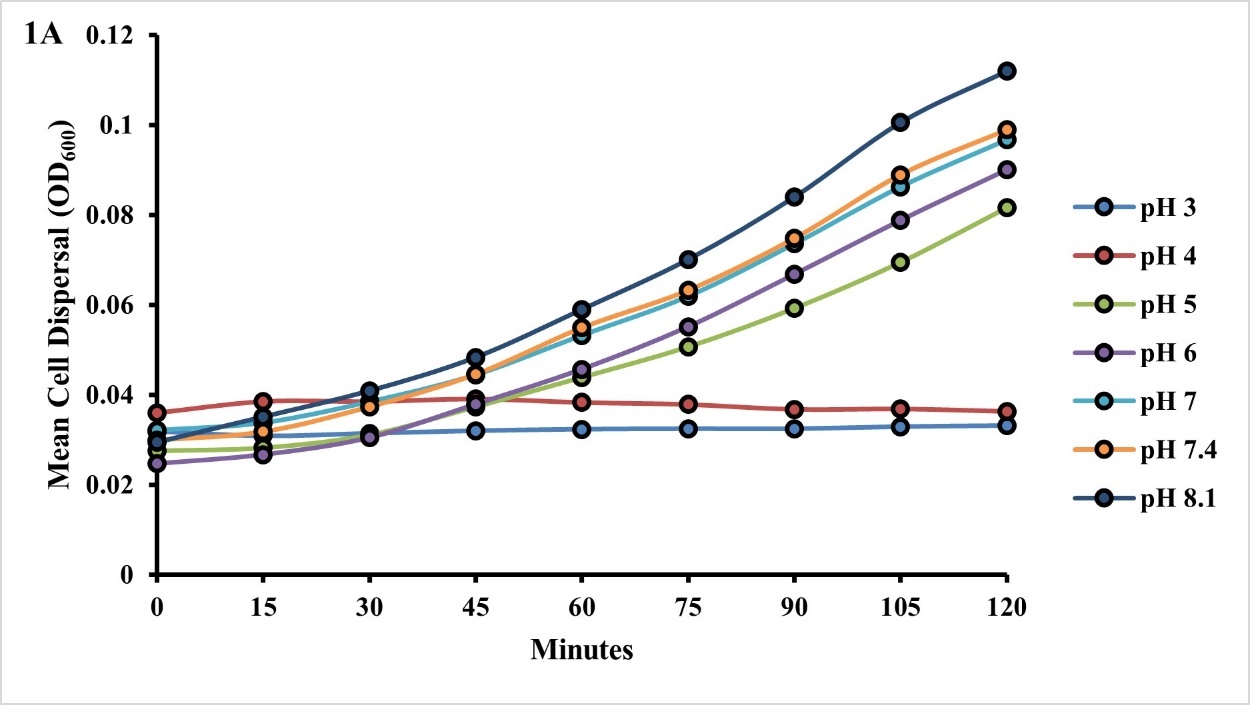


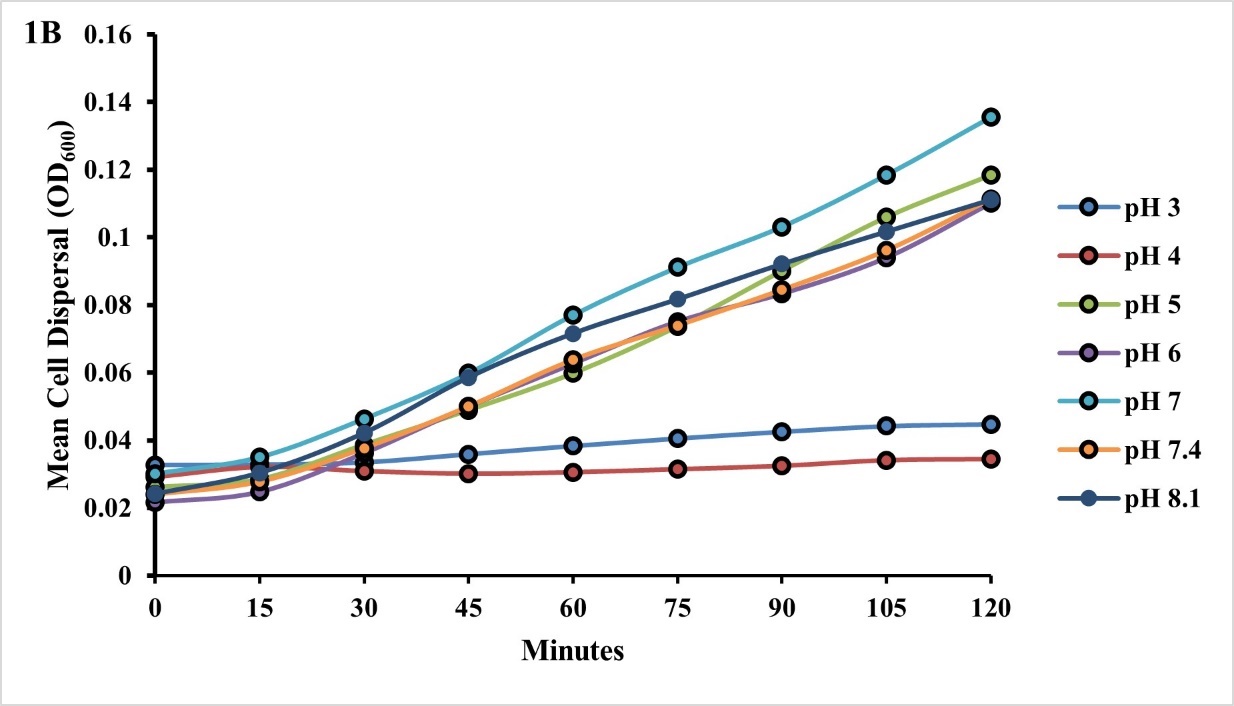


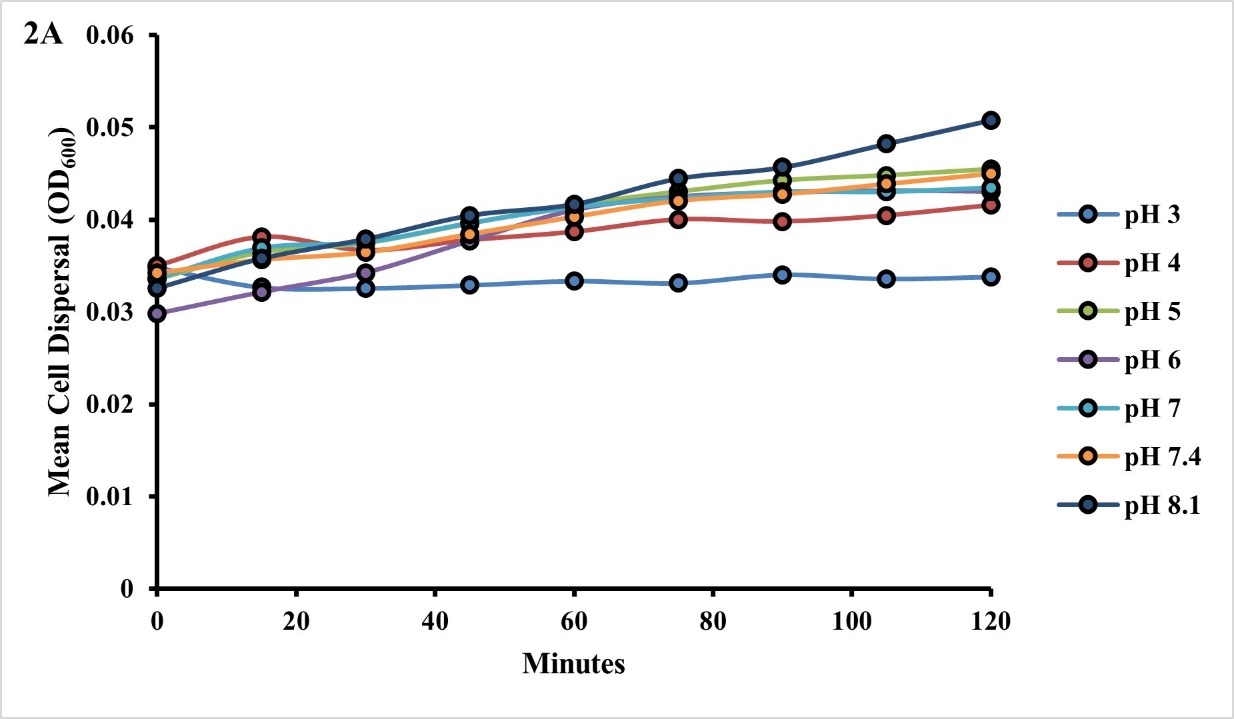


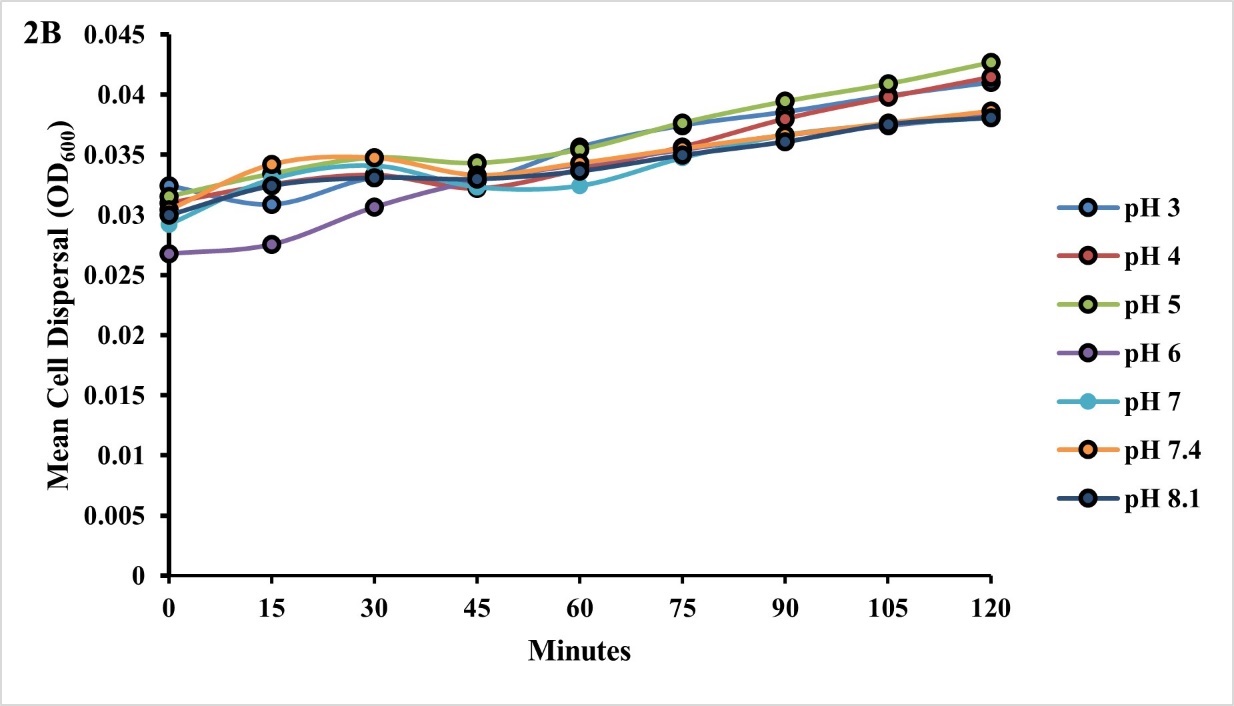


Figure S1. *V. parahaemolyticus* and *V. vulnificus* cell dispersal (OD_600_) over time at different pH and temperature in nutrient rich media. Comparison of cell dispersal (means of all biological triplicates and three independent experiments) after 2- hour exposure between *V. parahaemolyticus* at 25 °C (**1A**) and 37°C (**1B**) and *V. vulnificus* at 25 °C (**2A**) and 37°C (**2B**).

Table S63. *V. parahaemolyticus* and *V. vulnificus* mean cell dispersal^1^ values over time at different pH and temperature in nutrient rich media.

| **Species** | **Temp** | **Time (mins)** | **pH 3** | **pH 4** | **pH 5** | **pH 6** | **pH 7** | **pH 7.4** | **pH 8.1** |
| --- | --- | --- | --- | --- | --- | --- | --- | --- | --- |
| *V. parahaemolyticus* | 25°C | 0 | 0.0319 | 0.0360 | 0.0276 | 0.0247 | 0.0321 | 0.0299 | 0.0294 |
|  | 25°C | 15 | 0.0309 | 0.0386 | 0.0282 | 0.0267 | 0.0338 | 0.0318 | 0.0351 |
|  | 25°C | 30 | 0.0314 | 0.0386 | 0.0311 | 0.0304 | 0.0384 | 0.0373 | 0.0409 |
|  | 25°C | 45 | 0.0320 | 0.0391 | 0.0373 | 0.0379 | 0.0444 | 0.0446 | 0.0483 |
|  | 25°C | 60 | 0.0323 | 0.0383 | 0.0439 | 0.0457 | 0.0532 | 0.0549 | 0.0590 |
|  | 25°C | 75 | 0.0324 | 0.0379 | 0.0507 | 0.0551 | 0.0619 | 0.0632 | 0.0701 |
|  | 25°C | 90 | 0.0324 | 0.0368 | 0.0592 | 0.0668 | 0.0736 | 0.0748 | 0.0840 |
|  | 25°C | 105 | 0.0329 | 0.0369 | 0.0694 | 0.0788 | 0.0862 | 0.0889 | 0.1006 |
|  | 25°C | 120 | 0.0331 | 0.0363 | 0.0816 | 0.0900 | 0.0967 | 0.0989 | 0.1120 |
| *V. parahaemolyticus* | 37°C | 0 | 0.0327 | 0.0291 | 0.0261 | 0.0217 | 0.0301 | 0.0240 | 0.0241 |
|  | 37°C | 15 | 0.0328 | 0.0321 | 0.0284 | 0.0248 | 0.0350 | 0.0278 | 0.0303 |
|  | 37°C | 30 | 0.0334 | 0.0309 | 0.0387 | 0.0362 | 0.0463 | 0.0377 | 0.0421 |
|  | 37°C | 45 | 0.0359 | 0.0301 | 0.0489 | 0.0500 | 0.0598 | 0.0500 | 0.0586 |
|  | 37°C | 60 | 0.0383 | 0.0306 | 0.0598 | 0.0627 | 0.0770 | 0.0638 | 0.0716 |
|  | 37°C | 75 | 0.0406 | 0.0314 | 0.0737 | 0.0751 | 0.0911 | 0.0739 | 0.0817 |
|  | 37°C | 90 | 0.0426 | 0.0324 | 0.0901 | 0.0833 | 0.1031 | 0.0844 | 0.0921 |
|  | 37°C | 105 | 0.0442 | 0.0341 | 0.1060 | 0.0940 | 0.1184 | 0.0961 | 0.1017 |
|  | 37°C | 120 | 0.0448 | 0.0344 | 0.1184 | 0.1101 | 0.1356 | 0.1112 | 0.1111 |
| *V. vulnificus* | 25°C | 0 | 0.0063 | 0.0091 | 0.0091 | 0.0118 | 0.0082 | 0.0082 | 0.0083 |
|  | 25°C | 15 | 0.0066 | 0.0086 | 0.0091 | 0.0120 | 0.0081 | 0.0091 | 0.0081 |
|  | 25°C | 30 | 0.0061 | 0.0086 | 0.0114 | 0.0140 | 0.0097 | 0.0102 | 0.0093 |
|  | 25°C | 45 | 0.0060 | 0.0081 | 0.0130 | 0.0179 | 0.0121 | 0.0131 | 0.0117 |
|  | 25°C | 60 | 0.0064 | 0.0081 | 0.0164 | 0.0240 | 0.0169 | 0.0173 | 0.0162 |
|  | 25°C | 75 | 0.0071 | 0.0079 | 0.0216 | 0.0313 | 0.0240 | 0.0262 | 0.0229 |
|  | 25°C | 90 | 0.0070 | 0.0078 | 0.0286 | 0.0392 | 0.0341 | 0.0358 | 0.0300 |
|  | 25°C | 105 | 0.0068 | 0.0077 | 0.0353 | 0.0488 | 0.0467 | 0.0499 | 0.0401 |
|  | 25°C | 120 | 0.0064 | 0.0078 | 0.0439 | 0.0602 | 0.0560 | 0.0573 | 0.0537 |
| *V. vulnificus* | 37°C | 0 | 0.0048 | 0.0057 | 0.0070 | 0.0086 | 0.0058 | 0.0078 | 0.0067 |
|  | 37°C | 15 | 0.0049 | 0.0058 | 0.0082 | 0.0113 | 0.0072 | 0.0097 | 0.0082 |
|  | 37°C | 30 | 0.0060 | 0.0056 | 0.0133 | 0.0190 | 0.0127 | 0.0171 | 0.0152 |
|  | 37°C | 45 | 0.0067 | 0.0051 | 0.0204 | 0.0327 | 0.0277 | 0.0346 | 0.0302 |
|  | 37°C | 60 | 0.0093 | 0.0052 | 0.0307 | 0.0474 | 0.0524 | 0.0569 | 0.0513 |
|  | 37°C | 75 | 0.0114 | 0.0054 | 0.0472 | 0.0658 | 0.0753 | 0.0789 | 0.0679 |
|  | 37°C | 90 | 0.0123 | 0.0056 | 0.0677 | 0.0813 | 0.0901 | 0.0923 | 0.0750 |
|  | 37°C | 105 | 0.0116 | 0.0060 | 0.0819 | 0.0941 | 0.1008 | 0.1033 | 0.0816 |
|  | 37°C | 120 | 0.0119 | 0.0062 | 0.0999 | 0.1093 | 0.1131 | 0.1189 | 0.0881 |

^1^Optical density at 600 nm


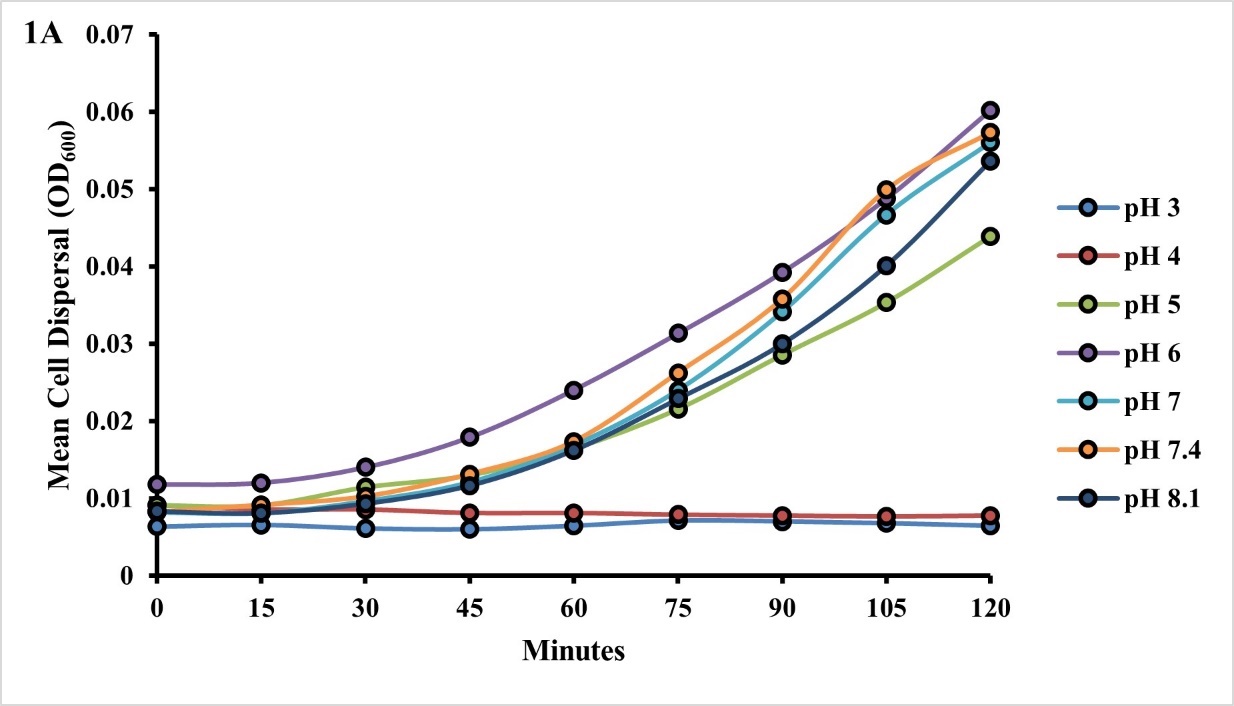


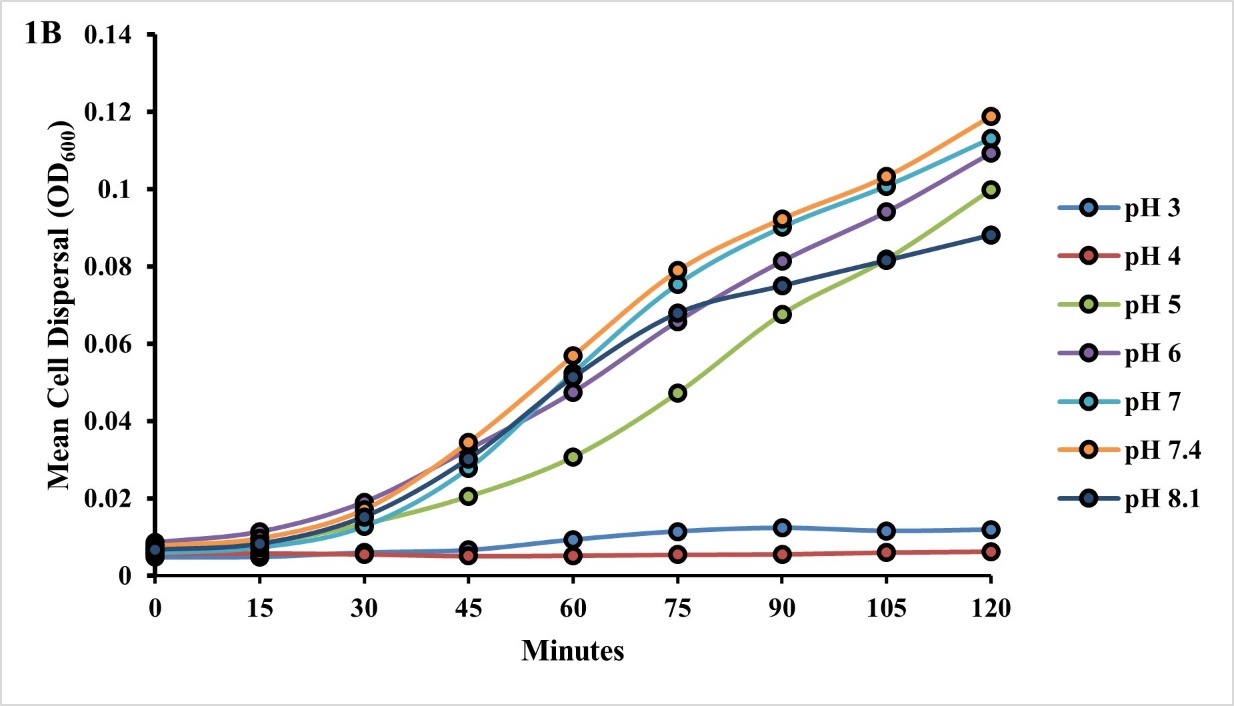


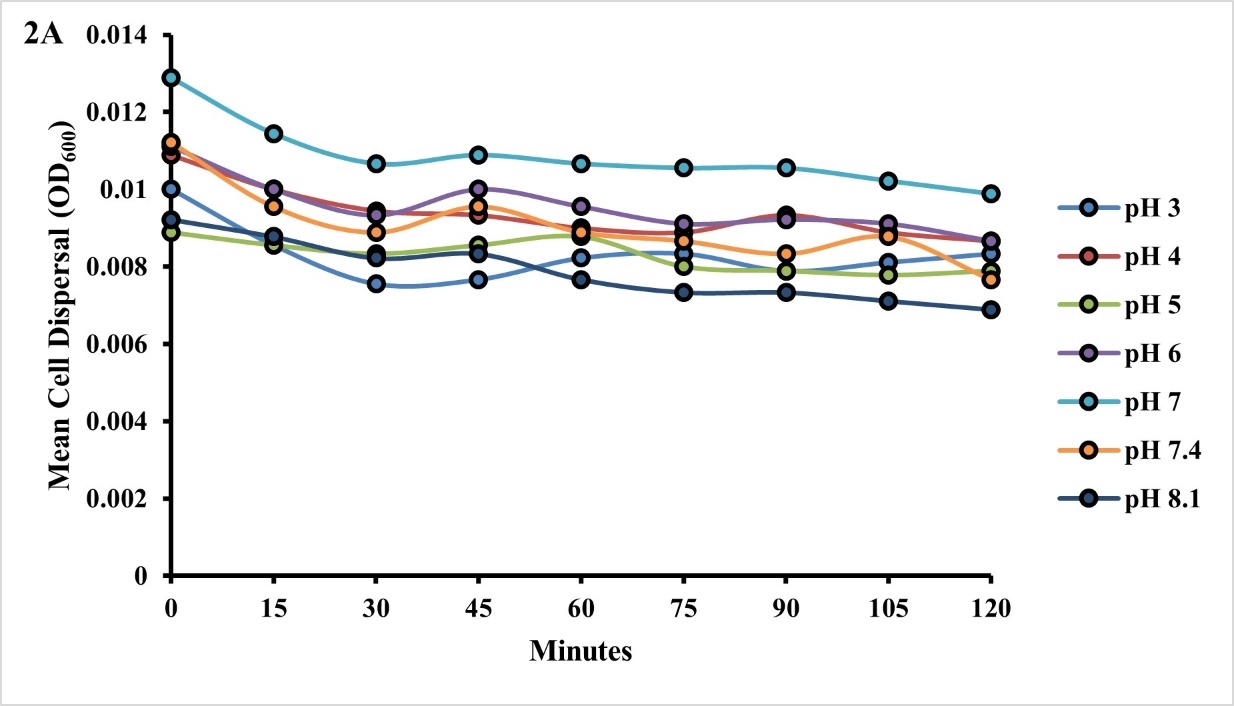


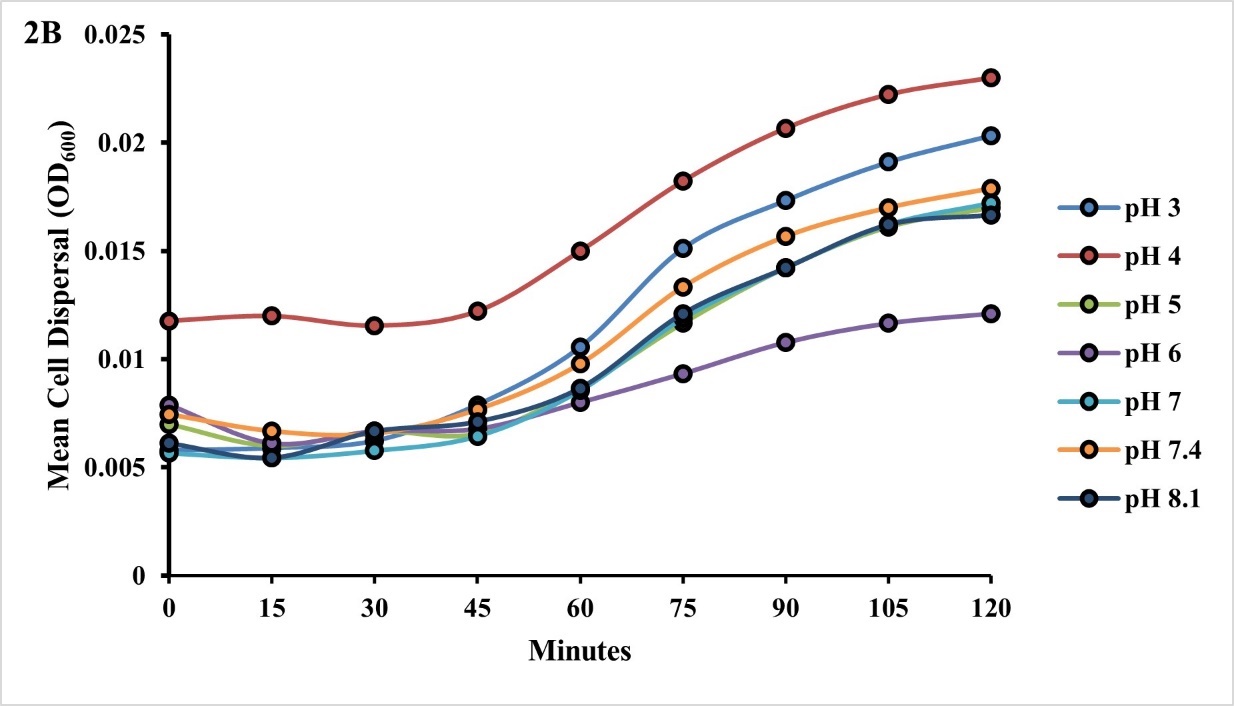


Figure S2. *V. parahaemolyticus* and *V. vulnificus* cell dispersal (OD_600_) over time at different pH and temperature in nutrient starved media. Comparison of cell dispersal (means of all biological triplicates and three independent experiments) after 2- hour exposure between *V. parahaemolyticus* at 25 °C (**1A**) and 37°C (**1B**) and *V. vulnificus* at 25 °C (**2A**) and 37°C (**2B**).

Table S64. *V. parahaemolyticus* and *V. vulnificus* mean cell dispersal^1^ values over time at different pH and temperature in nutrient starved media.

| **Species** | **Temp** | **Time (mins)** | **pH 3** | **pH 4** | **pH 5** | **pH 6** | **pH 7** | **pH 7.4** | **pH 8.1** |
| --- | --- | --- | --- | --- | --- | --- | --- | --- | --- |
| *V. parahaemolyticus* | 25°C | 0 | 0.0348 | 0.0350 | 0.0336 | 0.0298 | 0.0337 | 0.0342 | 0.0326 |
|  | 25°C | 15 | 0.0327 | 0.0381 | 0.0364 | 0.0321 | 0.0369 | 0.0356 | 0.0358 |
|  | 25°C | 30 | 0.0326 | 0.0367 | 0.0374 | 0.0342 | 0.0376 | 0.0364 | 0.0379 |
|  | 25°C | 45 | 0.0329 | 0.0378 | 0.0396 | 0.0377 | 0.0396 | 0.0384 | 0.0404 |
|  | 25°C | 60 | 0.0333 | 0.0387 | 0.0416 | 0.0410 | 0.0413 | 0.0403 | 0.0417 |
|  | 25°C | 75 | 0.0331 | 0.0400 | 0.0430 | 0.0424 | 0.0423 | 0.0420 | 0.0444 |
|  | 25°C | 90 | 0.0340 | 0.0398 | 0.0442 | 0.0429 | 0.0430 | 0.0427 | 0.0457 |
|  | 25°C | 105 | 0.0336 | 0.0404 | 0.0448 | 0.0431 | 0.0430 | 0.0439 | 0.0482 |
|  | 25°C | 120 | 0.0338 | 0.0416 | 0.0454 | 0.0430 | 0.0434 | 0.0450 | 0.0508 |
| *V. parahaemolyticus* | 37°C | 0 | 0.0324 | 0.0310 | 0.0316 | 0.0268 | 0.0292 | 0.0304 | 0.0300 |
|  | 37°C | 15 | 0.0309 | 0.0326 | 0.0334 | 0.0276 | 0.0330 | 0.0342 | 0.0324 |
|  | 37°C | 30 | 0.0331 | 0.0333 | 0.0348 | 0.0307 | 0.0341 | 0.0348 | 0.0331 |
|  | 37°C | 45 | 0.0329 | 0.0322 | 0.0343 | 0.0329 | 0.0323 | 0.0333 | 0.0330 |
|  | 37°C | 60 | 0.0357 | 0.0338 | 0.0354 | 0.0342 | 0.0324 | 0.0343 | 0.0337 |
|  | 37°C | 75 | 0.0374 | 0.0357 | 0.0377 | 0.0354 | 0.0348 | 0.0356 | 0.0350 |
|  | 37°C | 90 | 0.0386 | 0.0380 | 0.0394 | 0.0367 | 0.0367 | 0.0367 | 0.0361 |
|  | 37°C | 105 | 0.0399 | 0.0398 | 0.0409 | 0.0374 | 0.0376 | 0.0377 | 0.0376 |
|  | 37°C | 120 | 0.0410 | 0.0414 | 0.0427 | 0.0383 | 0.0381 | 0.0387 | 0.0381 |
| *V. vulnificus* | 25°C | 0 | 0.0100 | 0.0109 | 0.0089 | 0.0111 | 0.0129 | 0.0112 | 0.0092 |
|  | 25°C | 15 | 0.0086 | 0.0100 | 0.0086 | 0.0100 | 0.0114 | 0.0096 | 0.0088 |
|  | 25°C | 30 | 0.0076 | 0.0094 | 0.0083 | 0.0093 | 0.0107 | 0.0089 | 0.0082 |
|  | 25°C | 45 | 0.0077 | 0.0093 | 0.0086 | 0.0100 | 0.0109 | 0.0096 | 0.0083 |
|  | 25°C | 60 | 0.0082 | 0.0090 | 0.0088 | 0.0096 | 0.0107 | 0.0089 | 0.0077 |
|  | 25°C | 75 | 0.0083 | 0.0089 | 0.0080 | 0.0091 | 0.0106 | 0.0087 | 0.0073 |
|  | 25°C | 90 | 0.0079 | 0.0093 | 0.0079 | 0.0092 | 0.0106 | 0.0083 | 0.0073 |
|  | 25°C | 105 | 0.0081 | 0.0089 | 0.0078 | 0.0091 | 0.0102 | 0.0088 | 0.0071 |
|  | 25°C | 120 | 0.0083 | 0.0087 | 0.0079 | 0.0087 | 0.0099 | 0.0077 | 0.0069 |
| *V. vulnificus* | 37°C | 0 | 0.0058 | 0.0118 | 0.0070 | 0.0079 | 0.0057 | 0.0074 | 0.0061 |
|  | 37°C | 15 | 0.0059 | 0.0120 | 0.0060 | 0.0061 | 0.0054 | 0.0067 | 0.0054 |
|  | 37°C | 30 | 0.0062 | 0.0116 | 0.0067 | 0.0067 | 0.0058 | 0.0066 | 0.0067 |
|  | 37°C | 45 | 0.0079 | 0.0122 | 0.0066 | 0.0068 | 0.0064 | 0.0077 | 0.0071 |
|  | 37°C | 60 | 0.0106 | 0.0150 | 0.0087 | 0.0080 | 0.0086 | 0.0098 | 0.0087 |
|  | 37°C | 75 | 0.0151 | 0.0182 | 0.0117 | 0.0093 | 0.0119 | 0.0133 | 0.0121 |
|  | 37°C | 90 | 0.0173 | 0.0207 | 0.0142 | 0.0108 | 0.0142 | 0.0157 | 0.0142 |
|  | 37°C | 105 | 0.0191 | 0.0222 | 0.0161 | 0.0117 | 0.0162 | 0.0170 | 0.0162 |
|  | 37°C | 120 | 0.0203 | 0.0230 | 0.0170 | 0.0121 | 0.0172 | 0.0179 | 0.0167 |

^1^Optical density at 600 nm


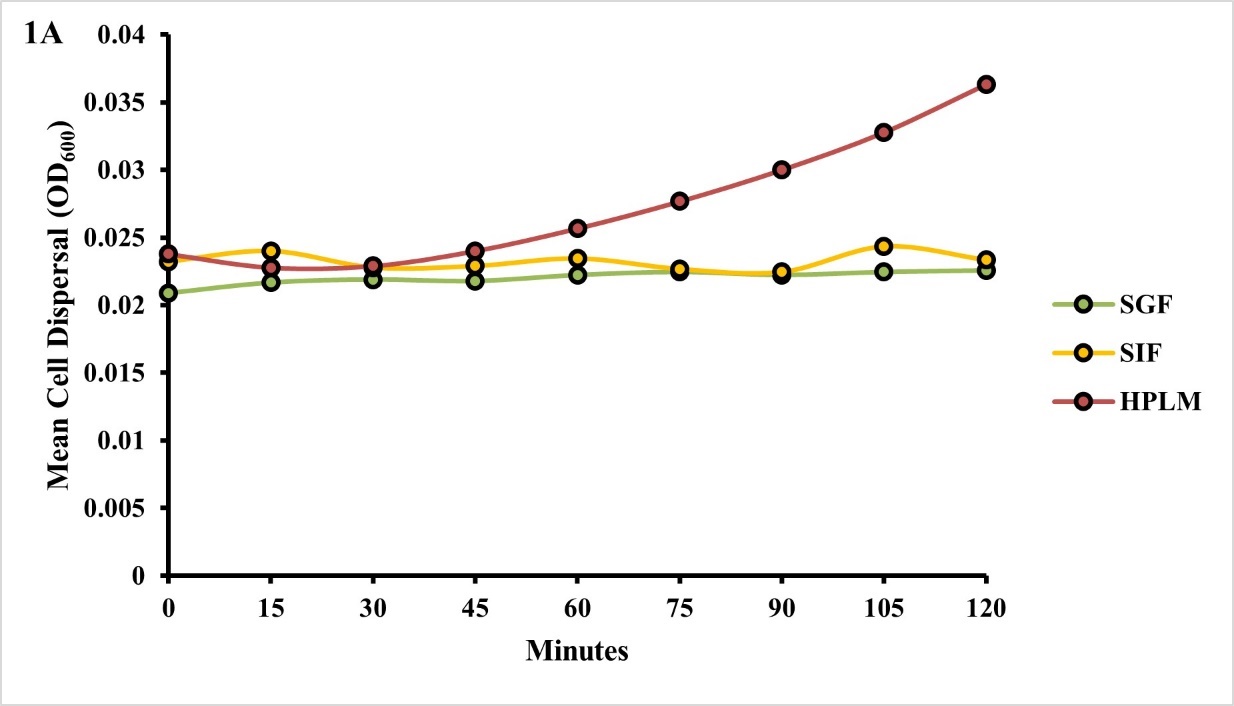

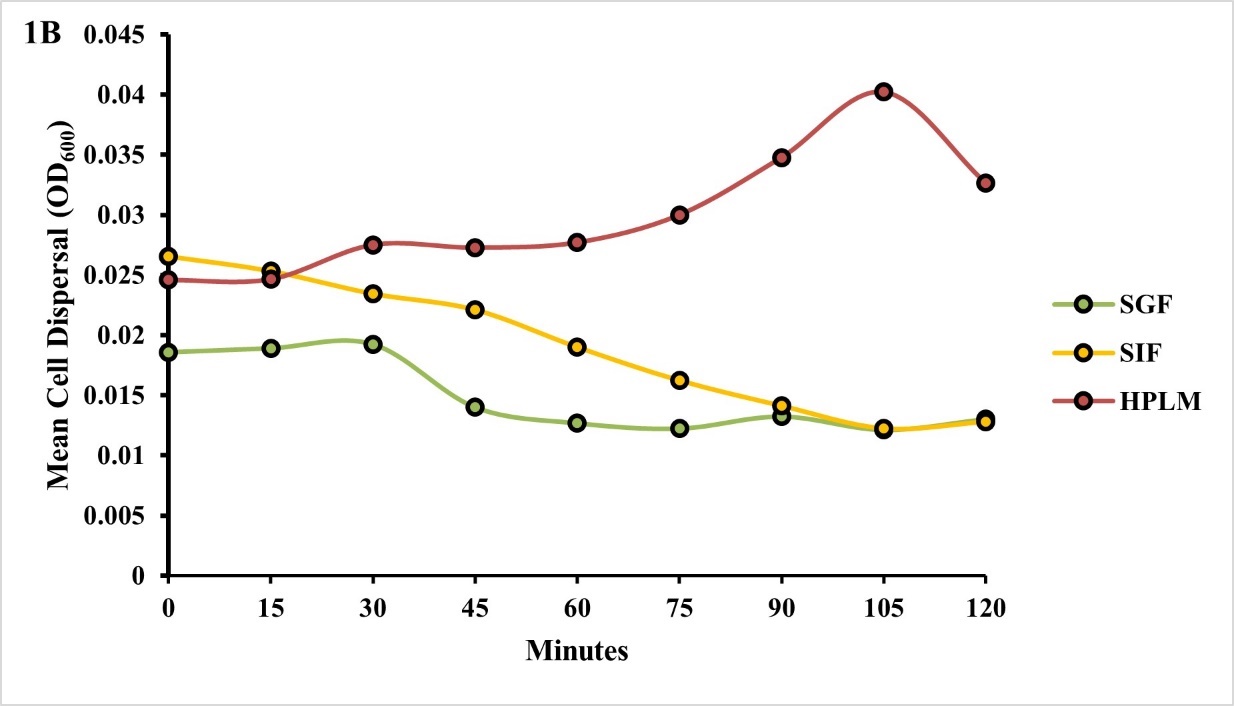


Figure S3. *V. parahaemolyticus* cell dispersal (OD_600_) over time at different temperature in simulated human media. Comparison of cell dispersal (means of all biological triplicates and three independent experiments) after 2- hour exposure between *V. parahaemolyticus* at 25 °C (**1A**) and 37°C (**1B**).

Table S65. *V. parahaemolyticus* mean cell dispersal^1^ values over time at different temperature in simulated human media.

| **Species** | **Temperature** | **Time (mins)** | **SGF** | **SIF** | **HPLM** |
| --- | --- | --- | --- | --- | --- |
| *V. parahaemolyticus* | 25°C | 0 | 0.0209 | 0.0232 | 0.0238 |
|  | 25°C | 15 | 0.0217 | 0.0240 | 0.0228 |
|  | 25°C | 30 | 0.0219 | 0.0228 | 0.0229 |
|  | 25°C | 45 | 0.0218 | 0.0229 | 0.0240 |
|  | 25°C | 60 | 0.0222 | 0.0234 | 0.0257 |
|  | 25°C | 75 | 0.0224 | 0.0227 | 0.0277 |
|  | 25°C | 90 | 0.0222 | 0.0224 | 0.0300 |
|  | 25°C | 105 | 0.0224 | 0.0243 | 0.0328 |
|  | 25°C | 120 | 0.0226 | 0.0233 | 0.0363 |
| *V. parahaemolyticus* | 37°C | 0 | 0.0186 | 0.0266 | 0.0246 |
|  | 37°C | 15 | 0.0189 | 0.0253 | 0.0247 |
|  | 37°C | 30 | 0.0192 | 0.0234 | 0.0275 |
|  | 37°C | 45 | 0.0140 | 0.0221 | 0.0273 |
|  | 37°C | 60 | 0.0127 | 0.0190 | 0.0277 |
|  | 37°C | 75 | 0.0122 | 0.0162 | 0.0300 |
|  | 37°C | 90 | 0.0132 | 0.0141 | 0.0348 |
|  | 37°C | 105 | 0.0121 | 0.0122 | 0.0402 |
|  | 37°C | 120 | 0.0130 | 0.0128 | 0.0327 |

^1^Optical density at 600 nm


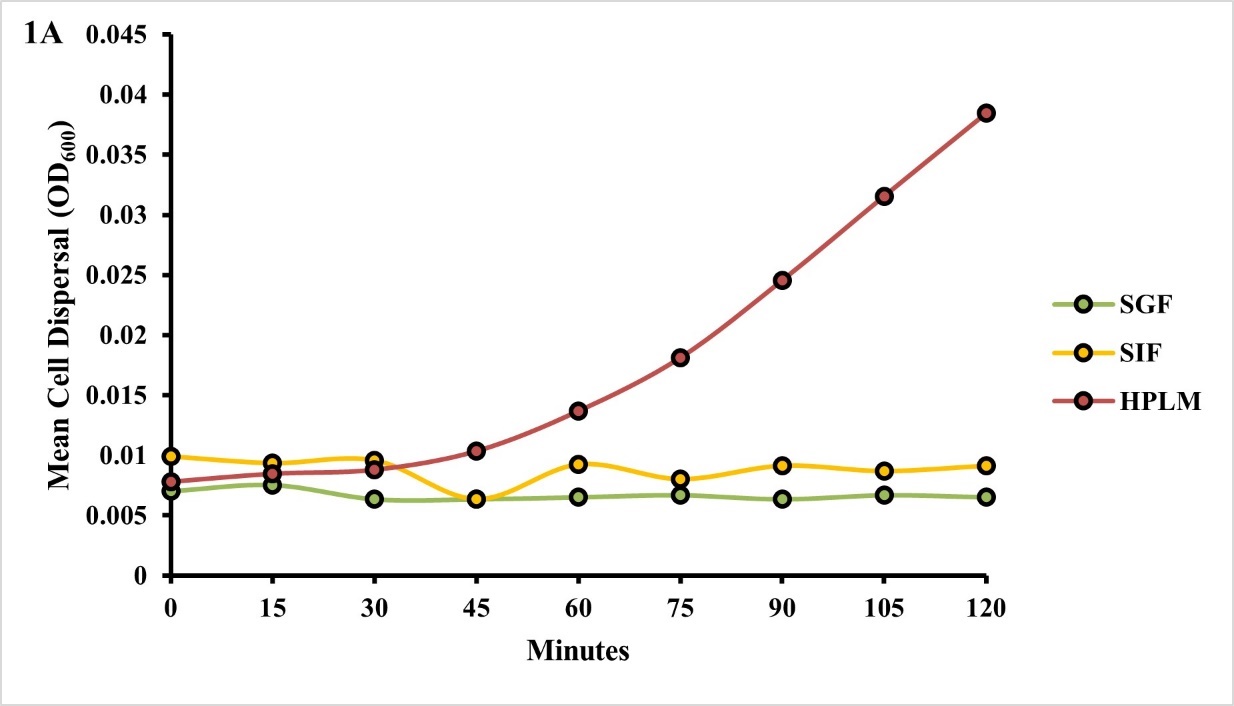

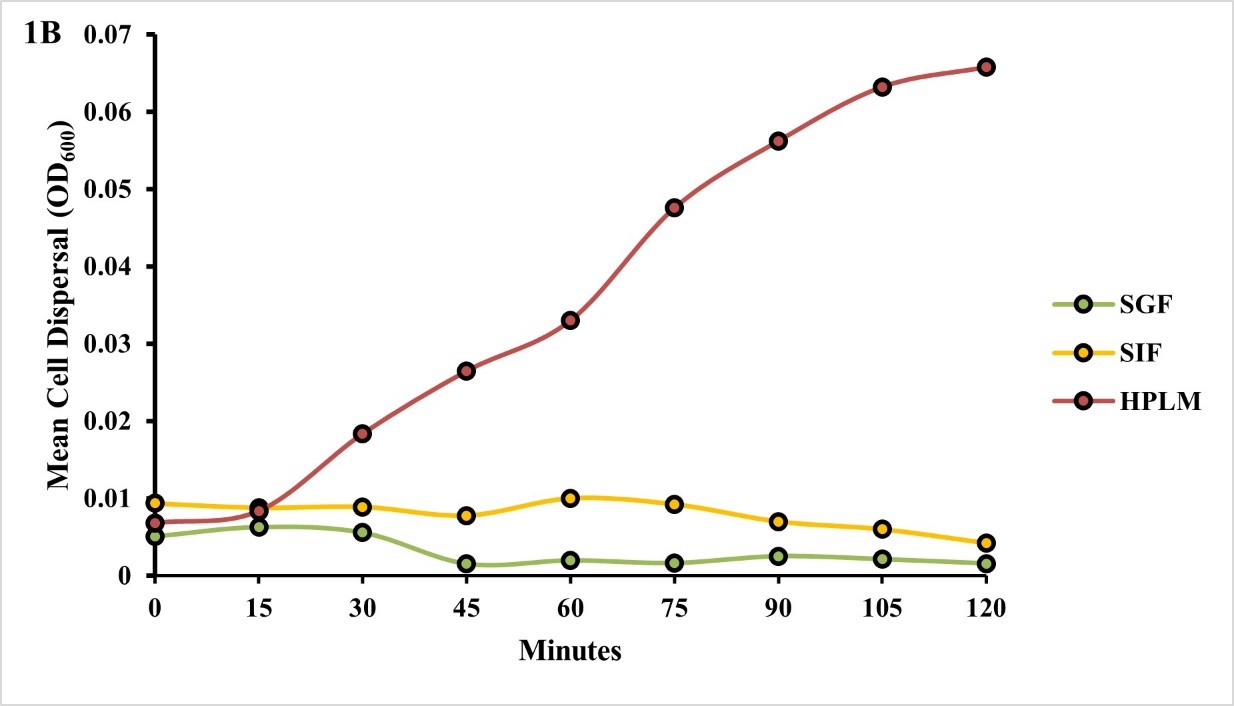


Figure S4. *V. vulnificus* cell dispersal (OD_600_) over time at different temperature in simulated human media. Comparison of cell dispersal (means of all biological triplicates and three independent experiments) after 2- hour exposure between *V. vulnificus* at 25 °C (**1A**) and 37°C (**1B**).

Table S66. *V. vulnificus* mean cell dispersal^1^ values over time at different temperatures in simulated human media.

| **Species** | **Temperature** | **Time (mins)** | **SGF** | **SIF** | **HPLM** |
| --- | --- | --- | --- | --- | --- |
| *V. vulnificus* | 25°C | 0 | 0.0070 | 0.0099 | 0.0078 |
|  | 25°C | 15 | 0.0075 | 0.0093 | 0.0084 |
|  | 25°C | 30 | 0.0063 | 0.0096 | 0.0088 |
|  | 25°C | 45 | 0.0063 | 0.0063 | 0.0103 |
|  | 25°C | 60 | 0.0065 | 0.0092 | 0.0137 |
|  | 25°C | 75 | 0.0067 | 0.0080 | 0.0181 |
|  | 25°C | 90 | 0.0063 | 0.0091 | 0.0246 |
|  | 25°C | 105 | 0.0067 | 0.0087 | 0.0316 |
|  | 25°C | 120 | 0.0065 | 0.0091 | 0.0384 |
| *V. vulnificus* | 37°C | 0 | 0.0051 | 0.0093 | 0.0068 |
|  | 37°C | 15 | 0.0063 | 0.0088 | 0.0083 |
|  | 37°C | 30 | 0.0056 | 0.0089 | 0.0183 |
|  | 37°C | 45 | 0.0015 | 0.0078 | 0.0264 |
|  | 37°C | 60 | 0.0019 | 0.0100 | 0.0330 |
|  | 37°C | 75 | 0.0016 | 0.0092 | 0.0476 |
|  | 37°C | 90 | 0.0025 | 0.0070 | 0.0562 |
|  | 37°C | 105 | 0.0021 | 0.0060 | 0.0632 |
|  | 37°C | 120 | 0.0016 | 0.0042 | 0.0658 |

^1^Optical density at 600 nm
